# Targeted sulfur(VI) fluoride exchange-mediated covalent modification of a tyrosine residue in the catalytic pocket of tyrosyl-DNA phosphodiesterase 1

**DOI:** 10.1038/s42004-024-01298-w

**Published:** 2024-09-16

**Authors:** Xue Zhi Zhao, Idris A. Barakat, George T. Lountos, Wenjie Wang, Keli Agama, Md Rasel Al Mahmud, Kiall F. Suazo, Thorkell Andresson, Yves Pommier, Terrence R. Burke

**Affiliations:** 1 Chemical Biology Laboratory, Center for Cancer Research, National Cancer Institute, National Institutes of Health, Frederick, MD USA; 2 Basic Science Program, Frederick National Laboratory for Cancer Research, Frederick, MD USA; 3 Developmental Therapeutics Branch & Laboratory of Molecular Pharmacology, Center for Cancer Research, National Cancer Institute, National Institutes of Health, Bethesda, MD USA; 4 Protein Characterization Laboratory, Cancer Research Technology Program, Frederick National Laboratory for Cancer Research, Frederick, MD USA

**Keywords:** X-ray crystallography, Bioconjugate chemistry, Structure-based drug design

## Abstract

Developing effective inhibitors of the DNA repair enzyme tyrosyl-DNA phosphodiesterase 1 (TDP1) has been challenging because of the enzyme shallow catalytic pocket and non-specific substrate binding interactions. Recently, we discovered a quinolone-binding hot spot in TDP1’s active site proximal to the evolutionary conserved Y204 and F259 residues that position DNA. Sulfur (VI) fluoride exchange (SuFEx) is a biocompatible click chemistry reaction that enables acylation of protein residues, including tyrosine. Selective protein modifications can provide insights into the biological roles of proteins and inform ligand design. As we report herein, we used SuFEx chemistries to prepare covalent TDP1-bound binders showing site-specific covalent bonds with Y204. Our work presents the first application of SuFEx chemistries to TDP1 ligands. It validates the ability to covalently modify specific TDP1 residues by designed targeting and adds to the chemical biology resource toolbox for studying TDP1.

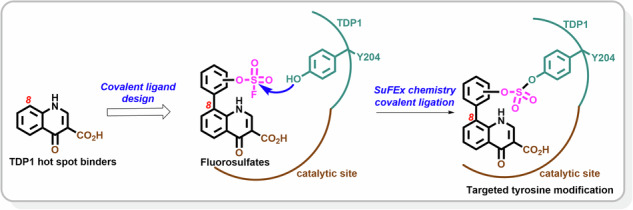

## Abstract (web summary)

Developing effective inhibitors of the DNA repair enzyme tyrosyl-DNA phosphodiesterase 1 (TDP1) is challenging because of the enzyme’s shallow catalytic pocket and non-specific substrate binding interactions. Here, the authors use Sulfur (VI) fluoride exchange chemistries to prepare covalent TDP1-bound binders showing site-specific covalent bonds with the Y204 residue that position DNA.

## Introduction

**Fig. 1:**
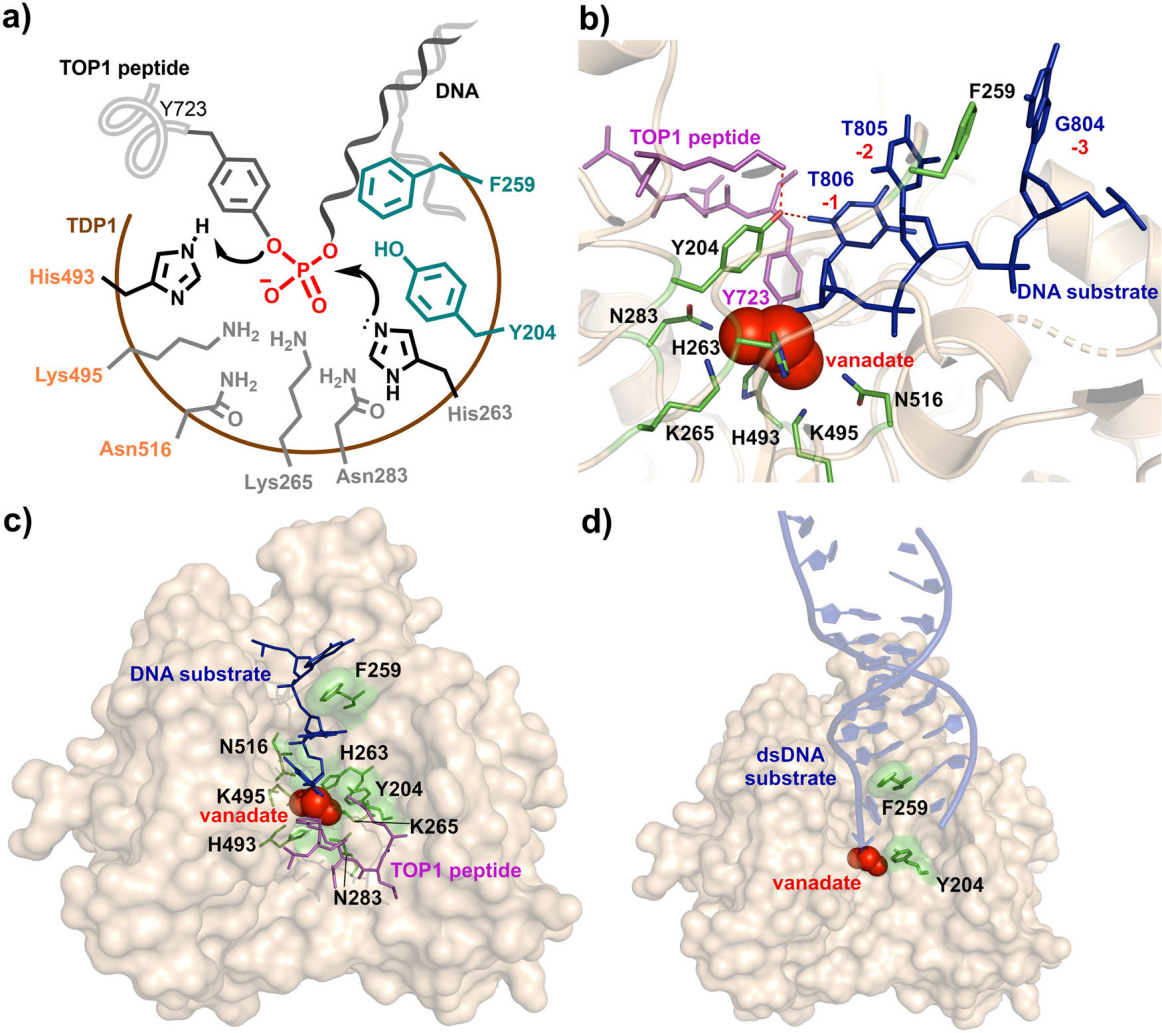
Structures of TDP1 and its catalytic site. **a** Schematic illustration of phosphodiester substrate and TDP1 catalytic site HKN motifs (black) and residues Y204 and F259 (cyan). **b** Crystal structure of TDP1(Δ1-148) bound with the vanadate (red spheres) complex of a TOP1-derived peptide (magenta sticks) and DNA substrate (blue sticks) with HKN motifs and Y204 and F259 highlighted (green sticks) (PDB code: 1NOP). **c** TDP1 tri-complex substrate mimetic with vanadate (red spheres), TOP-derived peptide (magenta sticks) and single-strand DNA substrate (blue sticks) and the catalytic HKN motifs of TDP1 (green sticks) (PDB code: 1NOP). **d** Crystal structure of TDP1(Δ1-148) complexed with double-stranded DNA (dsDNA) (blue strands) (PDB code: 5NWA). Y204 and F259 are depicted as green sticks. The position of the transition state mimetic vanadate (red spheres) is modeled based on superposition from the coordinates (PDB code: 1NOP).

Tyrosyl-DNA phosphodiesterase 1 (TDP1) is a 68-kDa enzyme that serves important roles in DNA repair by hydrolyzing phosphodiester bonds between terminal DNA-phosphates (primarily at 3’-DNA ends) and a broad range of chemical groups generated by endogenous and exogenous DNA damage^1–4^. One of the most studied functions of TDP1 is to excise stalled covalent complexes between the topoisomerase type IB enzyme (TOP1) and DNA, which represents critical mediators of response to anticancer TOP1 inhibitors^5^. TDP1 cleaves covalent complexes (ccs) formed between the TOP1 catalytic Tyr723 residue and 3’-phosphate groups of DNA (TOP1ccs) (Fig. 1a)^6^. Because hydrolysis of these TOP1ccs by TDP1 limits the effectiveness of TOP1 inhibitors^5,7,8^, chemical inhibition of TDP1 may potentiate the activity of TOP1 inhibitors in anticancer therapy. Indeed, genetic inactivation of TDP1 blocks the degradation of therapeutically critical TOP1-DNA adducts and synergizes with clinical TOP1 inhibitors^7–9^. In addition, inactivation of TDP1 is well tolerated in preclinical models^7^ and this has made development of TDP1 inhibitors an important research and medicinal chemistry objective^2,8,10,11^.

Although TDP1 is a potentially legitimate target for anticancer therapy in combination with TOP1 inhibitors, alkylating agents and radiation therapy^1–4^, there are no TDP1 inhibitors currently in clinical trials. This is due at least in part to the challenges posed by the open and shallow TDP1 catalytic site^6,12–16^. The nature of the catalytic machinery was revealed 20 years ago by crystal structure determination of the C-terminal phospholipase D domain of TDP1 (Δ1-148) bound to a TOP1-derived peptide, DNA substrate and a vanadate transition state mimetic (PDB code: 1NOP) (Fig. 1b, c)^6,12,17^. The primary catalytic apparatus consists of a pair of conserved histidine, lysine and asparagine residues (HKN motifs: H263/K265/N283 and H493/K495/N516) that form a pocket within which targets 3’-deoxyribose esters bind (Fig. 1)^6,17^. TDP1 catalysis proceeds by a two-step acid/base nucleophilic catalytic mechanism. The first nucleophilic attack is carried out by the conserved H263 residue imidazole nitrogen on the TOP1 Tyr723–3’-DNA phosphate moiety to release tyrosine and form a covalent phosphonamide TDP1–DNA complex (Fig. 1a). A second nucleophilic attack is then carried out by a water molecule activated by the H493 residue to release TDP1 and regenerate the active site^3,6,17–19^. In cells, TDP1 hydrolyzes phosphodiester bonds that originate from the TOP1-derived polypeptides covalently linked to nicked dsDNA molecules^20–22^. Several residues have been identified as being important for shaping the catalytic groove that accommodates the DNA substrate. These include Y204, which is evolutionarily conserved within the TDP1 active site, where it participates in the specific recognition and processing of DNA lesions^23^. Together with W590, a groove is formed above the HKN motifs near the catalytically targeted residues with Y204 forming two hydrogen bonds with the terminal (−1) nucleobase of the DNA substrate.

The binding of the DNA component has been informed recently by crystal structures of a double-stranded 12-mer DNA substrate bound to TDP1 (Δ1-148) (Fig. 1d)^12^. A conserved F259 is required for efficient DNA processing in biochemical assays as it assists in π-π stacking with the terminal (-2 and -3) nucleobases of the DNA substrate^12,23^. A hydrophobic wedge containing F259 disrupts base pairing, separates the DNA strands, and positions the DNA substrate for catalytic processing by the dual HKN motifs of TDP1^12,23^. The double-stranded duplex is bifurcated through intercalation and disruption of Watson-Crick base pairing by the aromatic ring of the F259 residue (Fig. 1d). Three nucleobases of the 3’-strand are directed down the narrow DNA binding channel to deliver the 3’-phosphoadduct to the catalytic pocket^12^. The unpaired complementary DNA strand is situated along a separate track of positive charge on the protein surface. The peptide portion of the protein-DNA substrate results from denaturation and proteolysis of the original TOP1 protein component to yield smaller sequences that are suitable for TDP1 catalysis^20–22,24^. Modeling of the TOP1 C-terminal residues 720-765 within the TDP1 peptide substrate cleft provides a sense of the potential extent of these binding interactions^17^. Mutations of Y204 or W590 to phenylalanine have limited or no impact on 3’-end substrate recognition and binding^23^.

The chemical biology toolbox for studying TDP1 could benefit from the ability to selectively modify specific residues. Given the strategic location of Y204 within TDP1’s catalytic pocket, we sought to develop ligands targeting this residue. Sulfonyl fluorides exhibit a distinctive balance of reactivity, stability and resistance to hydrolysis in biological conditions and are easily prepared using sulfur (VI) fluoride exchange (SuFEx)^25^. These electrophilic warheads are biocompatible and able to react with multiple natural amino acid residues, including tyrosine^26,27^. Therefore, these functional groups have been broadly applied in chemical biology, drug discovery, and biotherapeutics^28,29^. Aryl fluorosulfates have even greater hydrolytic stability and higher target selectivity, which has made them advantageous over sulfonyl fluorides for proteome labeling and “inverse drug discovery”^30^. These warheads are particularly valuable for the preferential labeling of tyrosine residues in protein pockets. In our current work, we developed a series of substituted quinolones with sulfonyl fluorides and phenylfluorosulfates tethered at the 8-position of the quinolones that were designed to specifically bind to Y204.

## Ligand design

### Quinolones as a platform for introducing sulfonyl fluoride functionality

**Fig. 2:**
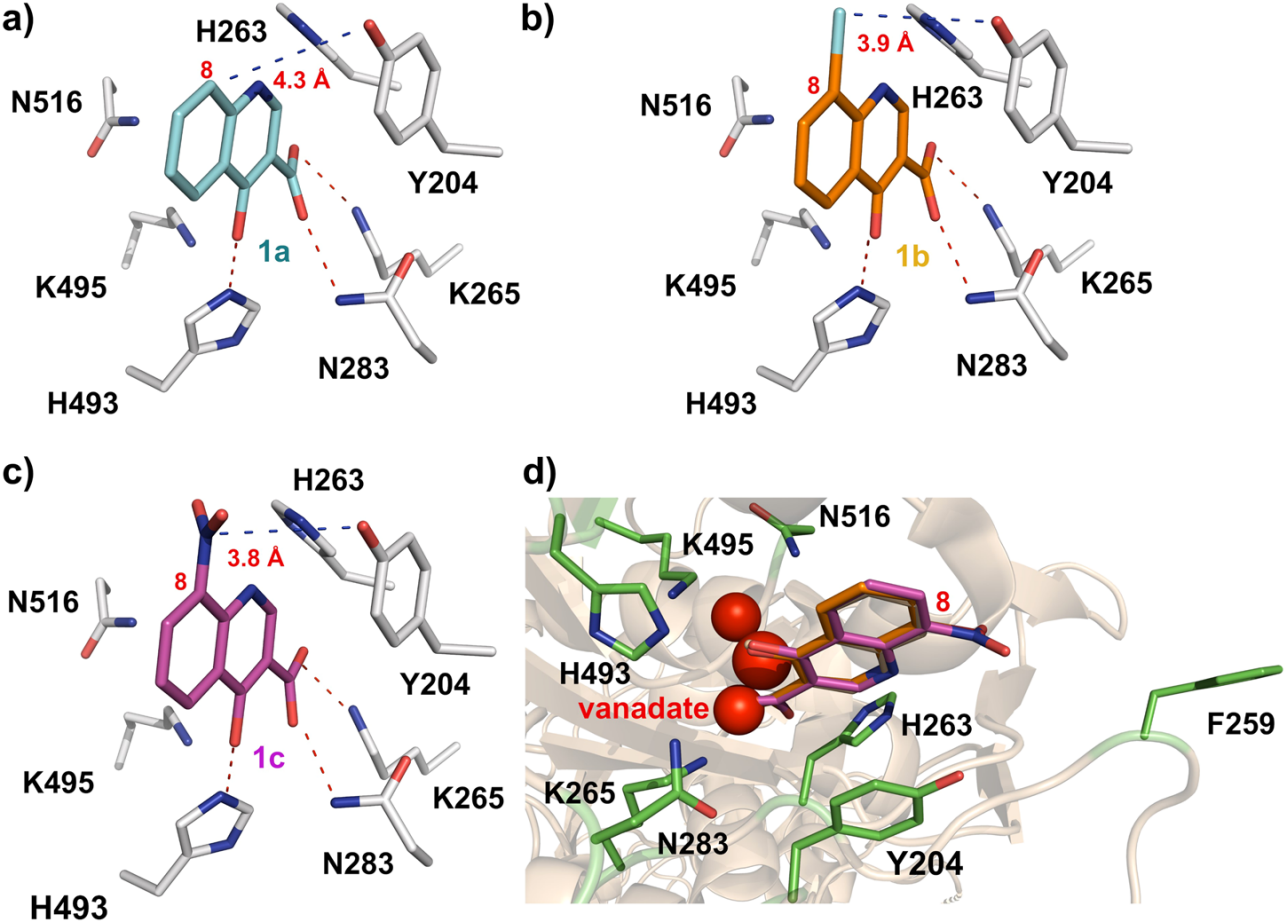
Structures of TDP1 bound to quinolones 1a – c. **a** Crystal structure of TDP1 bound to quinolone **1a** (carbon atoms in cyan) with the catalytic HKN motif highlighted (carbon atoms in gray) (PDB code: 6DIM). The hydroxyl of the Y204 residue has an approximate distance of 4.3 Å to the 8-position of compound **1a**. **b** Crystal structure of TDP1 bound to 8-bromo quinolone **1b** (carbon atoms in orange) with the catalytic HKN motif highlighted (PDB code: 6DJH). The hydroxyl of the Y204 residue has an approximate distance of 3.9 Å to the 8-bromo of compound **1b**. **c** Crystal structure of TDP1 bound to 8-nitro quinolone **1c** (carbon atoms in magenta) with the catalytic HKN motif highlighted (green) (PDB code: 6MJ5). The hydroxyl of the Y204 residue has an approximate distance of 3.8 Å to the 8-nitro of compound **1c**. **d** Superimposed structures of TDP1 bound to quinolones **1a** – **c** with the catalytic HKN motif residues, the residues of Y204 and F259 are highlighted (carbon atoms in green). The position of the vanadate (red spheres) is modeled based on superposition from crystal structure coordinates (PDB code: 1NOP).

**Fig. 3:**
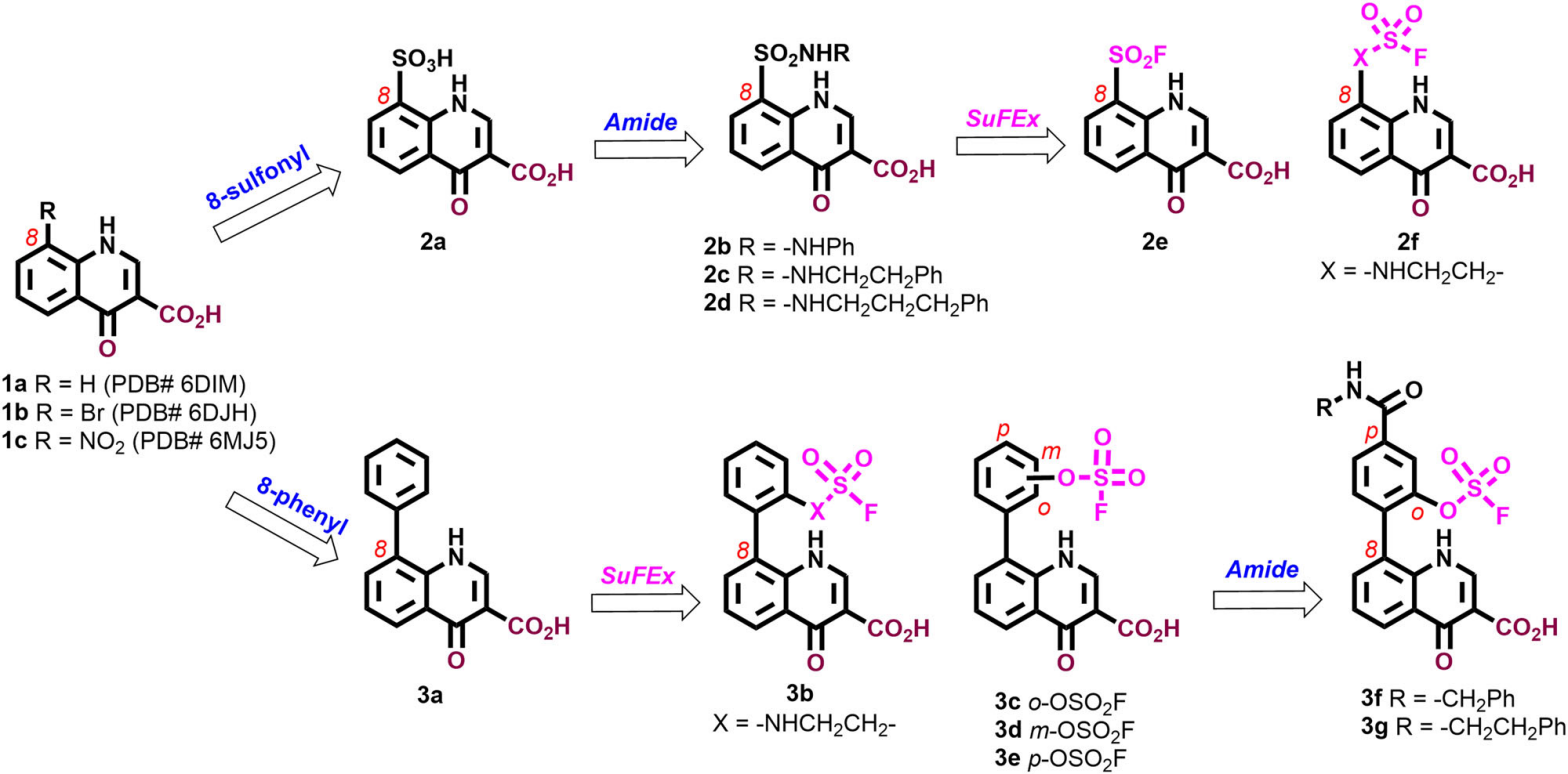
Design evolution of the quinolone derivatives 2a – f and 3a – g. The catalytic site binding groups including carbonyl and carboxylic acid are highlighted in brown. Covalent functional groups in precursors including fluorosulfonyl or fluorosulfate groups are highlighted in neon purple.

Quinolones represent a class of broad-spectrum antibiotics that are widely used for treating a variety of infections. These compounds inhibit bacterial DNA synthesis by disrupting type II topoisomerases, DNA gyrase and topoisomerase IV^31–37^. Previously, we employed a crystallographic fragment cocktail screen of more than 600 small molecule fragments to discover quinolones, such as **1a – c**, that bind within the TDP1 catalytic site (Fig. 2)^13^. The 4-oxygen and 3-carboxylic acid moieties of the quinolines interact with the catalytic HKN motifs and replicate aspects of substrate phosphate by forming several direct hydrogen bonds with the key catalytic residues. The carboxylate moieties reproduce aspects of the transition state mimetic vanadate bound to the TDP1 catalytic pocket. Carboxylate and hydroxyl oxygens of the quinolone ligands overlap with oxygen atoms of the vanadate molecule, making direct hydrogen bonds with the side chains of N283 and K265. The structure of quinolone **1a** bound to the TDP1 catalytic pocket shows that the 8-position carbon is in close proximity (4.3 Å) to the phenolic hydroxyl of the Y204 side chain (PDB code: 6DIM; Fig. 2a)^13^. Structures of related quinolones bound to TDP1 also reveal close proximity of substituents to the Y204 phenolic hydroxyl, including the 8-bromo group of **1b** (3.9 Å, PDB code: 6DJH; Fig. 2b) and the 8-nitro group of **1c** (3.8 Å, PDB code: 6MJ5; Fig. 2c)^13^. This suggests that the quinolone 8-position is ideally positioned to specifically target Y204 for covalent bond formation. The F259 residue, which serves as a hydrophobic wedge to split the double strand DNA substrate “zipper”, is also located in close proximity ( ~ 11 Å) to the 8-position of the TDP1-bound quinolones **1a – c** (Fig. 2d). Based on these considerations, we designed a range of quinolone analogs bearing SuFEx functionality at the 8-position (Fig. 3).

We prepared two series of 8-substituted quinolone analogs to explore potential binding interactions within and proximal to the catalytic pocket (Fig. 3 and Supplementary Methods in Supplementary Information). These included 8-sulfonyl quinolone analogs (**2a – f**) and 8-phenyl quinolone analogs (**3a – g**) (Fig. 3). The 8-sulfonic acid (**2a**) provided access to a series of 8-sulfonylamides tethering phenyl rings by means of various length alkyl chains (**2b – d**). We also prepared the 8-fluorosulfonyl-containing analog (**2e**) and the 8-fluorosulfonylethylamino-containing analog (**2f**). The tethered aryl groups in **2b – d** were intended to engage hydrophobic interactions with the key phenyl ring of the F259, which has been shown to play important roles in TDP1 function by positioning the DNA substrate in its binding channel^12,23^. We also examined a parallel series of 8-phenyl-containing analogs (**3a – g**) as the 8-phenyl ring appeared to potentially afford contact with the Y204 hydroxyl. A range of compounds could be readily synthesized using Suzuki coupling reactions^38^ including the parent 8-phenyl analog (**3a**) as well as a series of isomeric 8-phenyl analogs substituted with 2-flurorosulfonylethylamino, and 2-, 3- and 4-fluorophosphate groups (compounds **3b,** **3c,** **3d** and **3e**, respectively). As described below, we were able to solve the X-ray crystal structure of the 2-fluorosulfate substituted analog (**3c**) covalently bound within the TDP1 catalytic pocket. Based on this latter structure, we introduced a carboxamide group at the 4-position of the *ortho*-phenylfluorosulfate moiety and tethered aromatic substituents from the *para*-amides (**3f** and **3g**) to engage hydrophobic interactions with the phenyl ring of the F259 residue.

## Results and discussion

### X-ray crystallography

We had previously reported the structures of the parent quinolones **1a** – **c** bound within the TDP1 catalytic pocket (Fig. 2)^13^. In the present work, we solved the crystal structures of TDP1 in complex with the six additional quinolone derivatives **2a,** **2e,** **3a,** **3b,** **3c**, and **3e** separately. We were also able to collect X-ray diffraction data of TDP1 crystals soaked with two additional compounds **2f** and **3f** (crystallographic data collection and refinement statistics are presented in Table S1 in Supplementary Information). Compound **2e** contains a sulfonylfluoride moiety and compounds **2f** and **3b** contain ((2-fluorosulfonyl)ethyl)aminophenyl groups, while compounds **3c,** **3e**, and **3f** contain phenyl fluorosulfate groups. Although we observed electron density indicating the covalent binding of the quinoline core scaffold to Y204, we did not observe electron density for the amide R groups in compound **3f**. This suggests that these parts of moieties are disordered and likely adopt multiple conformations.

**Fig. 4:**
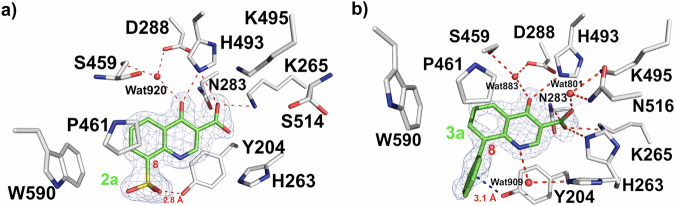
Structures of TDP1 bound to quinolones 2a (PDB code: 6DJG) and 3a (PDB code: 6MYZ). **a** Crystal structure of TDP1 bound to quinolone **2a** (carbon atoms in green) with the catalytic pocket highlighted (carbon atoms in gray). The fit of **2a** to the 2*F*_o_-*F*_c_ electron density map (1.88 Å resolution, blue mesh, contoured at 1.0 σ level) is shown. The hydroxyl of the Y204 residue has an approximate distance of 2.8 Å to the 8-sufonyl of compound **2a**. **b** Crystal structure of TDP1 bound to quinolone **3a** (carbon atoms in green) with the catalytic pocket highlighted (carbon atoms in gray). The hydroxyl of the Y204 residue has an approximate distance of 3.1 Å to the 8-phenyl of compound **3a**. The fit of **3a** to the 2*F*_o_-*F*_c_ electron density map (1.66 Å resolution, blue mesh, contoured at 1.0 σ level) is shown.

Compounds **2a** and **3a** lack chemically reactive functionality that would be necessary to interact covalently with the protein (Fig. 4). The crystal structures of TDP1 in complex with these ligands indicate that they are situated within the catalytic site in similar binding modes, with their hydroxyls and carboxylic acids forming H-bonds with catalytic residues. The crystal structures of TDP1 in complex with compounds **2a** and **3a** were determined at 1.88 Å (PDB code: 6DJG) and 1.66 Å resolution (PDB code: 6MYZ), respectively. Their binding mode reveals that the core structure retains the same binding interactions as the parent molecules **1a – c**, while the 8-substituent extends into the DNA binding region of TDP1^13^. The 8-sulfonic acid group in **2a** and the 8-phenyl moiety in **3a** project toward the DNA substrate-binding area. The 8-sulfonic acid moiety of compound **2a** is hydrogen bonded to the phenolic oxygen atom of Y204 via one of the sulfate oxygen atoms (2.8 Å) and is located within 3.5 Å of the W590 side chain (Fig. 4a). The 8-phenyl moiety of compound **3a** is surrounded by and positioned near residues Y204 (3.1 Å), P461 (3.9 Å), and W590 (4.5 Å) (Fig. 4b). When the structures are superimposed onto the structure of TDP1 bound to a TOP1 derived peptide and ssDNA, the 8-phenyl and 8-sulfonic acid groups occupy the binding site of the DNA substrate to the T805 nucleobase on TDP1 (Fig. 1b).

**Fig. 5:**
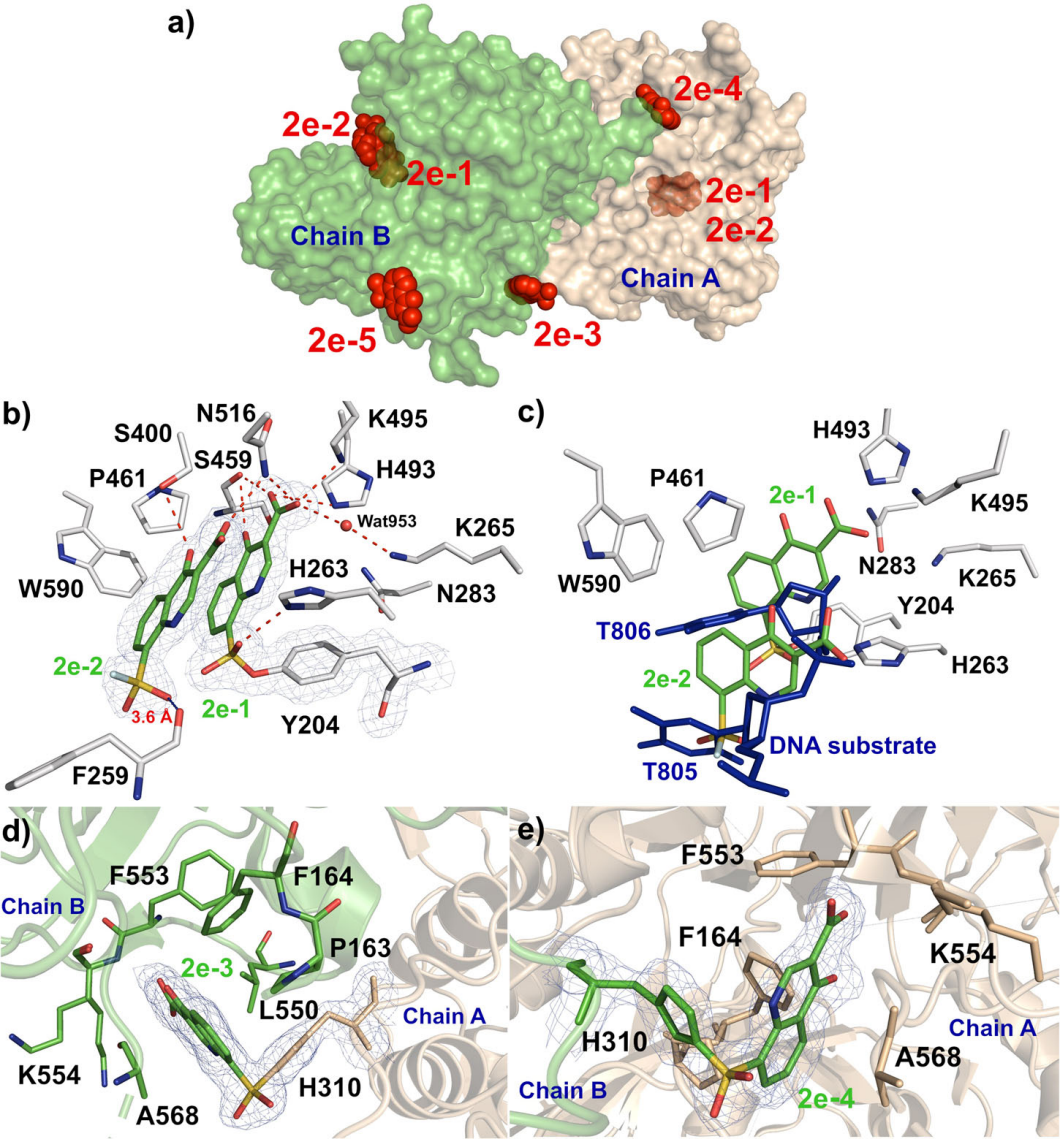
Structures of TDP1 bound to the 8-fluorosulfonyl quinolone 2e molecules (PDB code: 8UV1). **a** Illustration showing identified binding sites of **2e** molecules (red spheres) to TDP1 at the catalytic site (for **2e-1, 2e-2**) and crystallographic dimer packing interface (for **2e-3, 2e-4, 2e-5**). **b** Structure of molecules of quinolone **2e-1** (carbon atoms in green) covalently bound to Y204 and interactions with the catalytic pocket. One molecule of quinolone **2e-2** (carbon atoms in green) π-π stacks with the covalently bound **2e-1**. Fit of the compounds to the 2*F*_o_-*F*_c_ electron density map (1.83 Å resolution, blue mesh, contoured at 1.0 σ level) is shown. **c** Structure of the TDP1 catalytic site bound to molecules of quinolone **2e-1** and **2e-2** overlaid with the coordinates of TDP1 complexed with DNA substrate (blue sticks, PDB code: 1NOP). **d** Structure of quinolone **2e-3** (green sticks) covalently bound to the crystal packing interface surface residue H310 on chain A (wheat). **e** Structure of the TDP1 crystallographic packing interface showing the quinolone **2e-4** (green sticks) covalently bound to the residue H310 on chain B (green).

Because we observed that the sulfate moiety of **2a** is located within a hydrogen bonding distance of the phenolic hydroxyl oxygen of Y204, we converted the 8-sulfonic acid of **2a** to the sulfonyl fluoride **2e** postulating that within this distance a covalent bond could form with the Y204 phenolic hydroxyl (Fig. 3). A 1.83 Å resolution crystal structure of TDP1 in complex with several molecules of **2e** was determined (PDB code: 8UV1, Fig. 5a). The locations of the binding spots of these molecules are shown in Fig. 5a. Unambiguous electron density confirmed the covalent binding of the **2e-1** sulfonyl fluoride to the side chain of Y204 (Fig. 5b). The carboxylic acid head group of the quinolone hydrogen bonds to the catalytic residues H493, K495, and the backbone carbonyl oxygen atom of S459. An oxygen atom of the sulfonyl group also hydrogen bonds to the side chain of H263. Due to the formation of the covalent bond, the bicyclic quinolone plane of **2e-1** undergoes a tilt of ~40° angle when compared with **1a**, which lacks the 8-sulfonyl fluoride group, while the bicyclic rings of **2a** retain the same plane as **1a**. Additionally, the oxygen atom on the 4-position of the quinolone scaffold forms a hydrogen bond with the backbone carbonyl oxygen of S459. A second **2e-2** molecule was also observed to bind in the catalytic pocket, where it π-π stacks with the first covalently bound molecule of **2e-1** and is located within 3.6 Å of the backbone main chain of residue F259 (Fig. 5b, c). This second molecule of **2e-2** did not covalently bind to the active site and thus was unreactive. In addition to the π-π stacks against the covalently bound **2e-1**, this second molecule binds to the active site via hydrogen bonds by one of the carboxylic acid hydrogen atoms to the side chain of N516 and the carbonyl oxygen at the 4-position of the quinolone hydrogen bonds to the side chain of S400. Interestingly, when the structure of TDP1 in complex with **2e-1** and **2e-2** is superimposed onto the coordinates of TDP1 bound to a DNA substrate (PDB code: 1NOP), the second molecule of **2e-2** overlaps with the binding site of thymidine 805 of the bound DNA substrate, while the covalently bound **2e-1** molecule overlaps with the binding site of thymidine 806 of the bound DNA substrate (Fig. 5c). We observed an additional covalent binding site of **2e-3** at the crystallographic TDP1 dimer surface where it forms a covalent bond with the H310 residue in Chain A (wheat sticks, Fig. 5d). The quinolone scaffold is inserted into a pocket formed by packing of Chain A against Chain B where it packs against residues A568, K554, F553, F164, L550, and P163 of Chain B (green sticks, Fig. 5d). Similarly, **2e-4** is observed to be covalently bound to H310 of Chain B (green sticks, Fig. 5e) and interacts with a pocket formed by packing against residues from Chain A (wheat sticks, Fig. 5e). As the structure of TDP1 complexed with compound **2e** was obtained by soaking TDP1 crystals with the compound, this may potentially be due to crystallographic artifacts. Also, of note, compound **2e** lacks an aryl extension at the 8-position which may provide specificity determinants for arylsulfates (Fig. 3). Another, unreacted molecule of **2e-5** was also inserted in a small pocket formed by crystallographic packing symmetry mates of Chain B formed by packing against residues P176 and K177 of Chain B against residues P217 and R220 in a crystallographic symmetry mate of Chain B. The observation of these additional binding sites, although potentially crystallographic artifacts, could also emphasize the importance of the 8-arylsulfonate moiety in imparting specificity during covalent ligation.

**Fig. 6:**
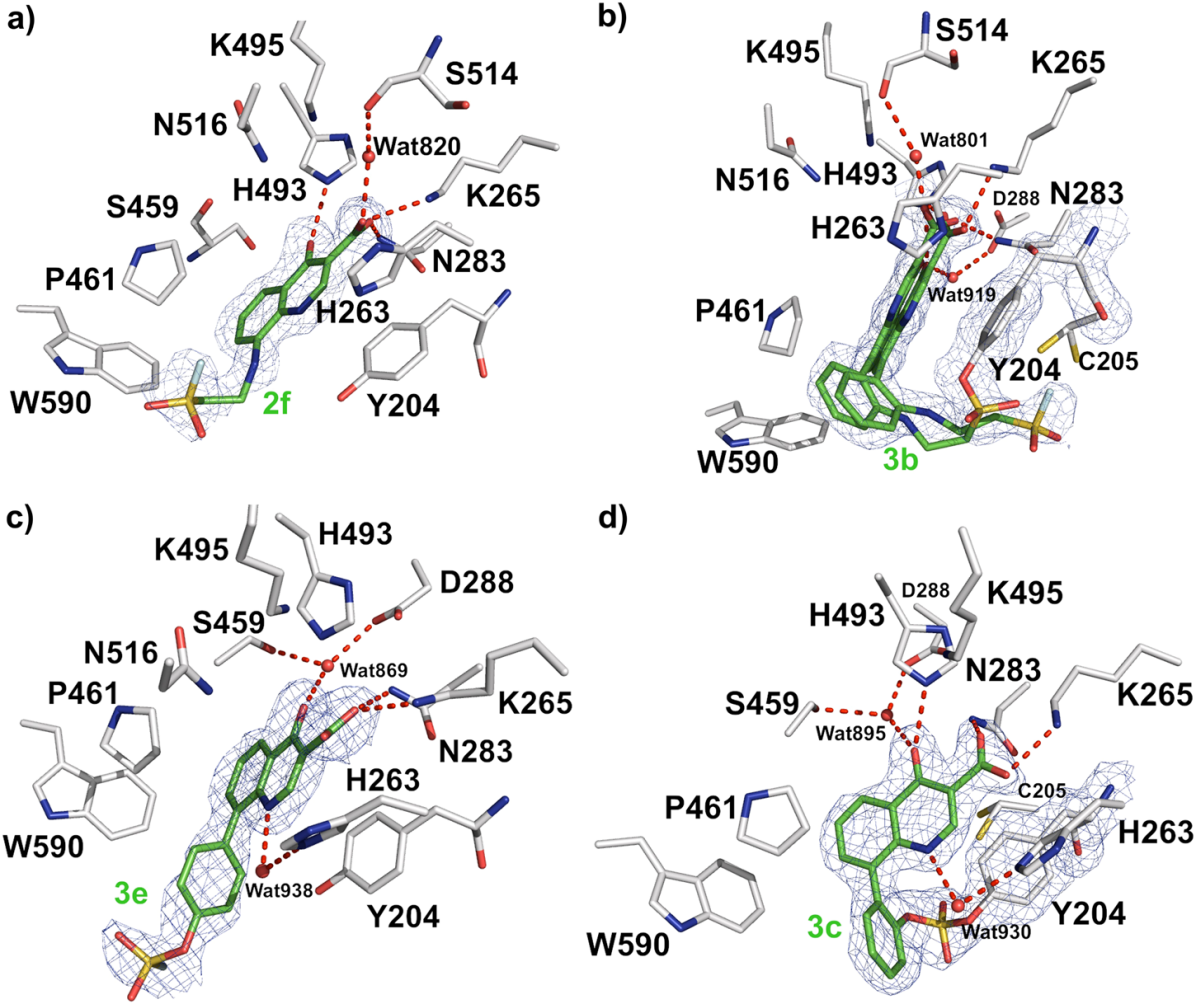
Structures of quinolones 2f (PDB code: 8UZV), 3e (PDB code: 8V0B), 3b (PDB code: 8V0C) and 3c (PDB code: 8UZZ) bound at the TDP1 catalytic site. **a** Crystal structure of quinolone **2f** (carbon atoms in green) bound to TDP1’s catalytic pocket (carbon atoms in gray). The fit of **2f** to the 2*F*_o_-*F*_c_ electron density map (1.85 Å resolution, blue mesh, contoured at 1.0 σ level) is shown. **b** Crystal structure of quinolone **3b** covalently bound to residue Y204 adjacent to the TDP1 catalytic site. An unreacted molecule **3b** was also observed. The fit of **3b** to the 2*F*_o_-*F*_c_ electron density map (1.62 Å resolution, blue mesh, contoured at 1.0 σ level) is shown. **c** Crystal structure of quinolone **3e** bound to TDP1’s catalytic pocket. The fit of **3e** to the 2*F*_o_-*F*_c_ electron density map (1.65 Å resolution, blue mesh, contoured at 1.0 σ level) is shown. **d** Crystal structure of quinolone **3c** covalently bound to the residue Y204 with a sulfate bond at the TDP1 catalytic site. The fit of **3c** to the 2*F*_o_-*F*_c_ electron density map (1.93 Å resolution, blue mesh, contoured at 1.0 σ level) is shown.

A 1.85 Å resolution data set was collected from a TDP1 crystal soaked with compound **2f**, which contains a flexible 8-ethylamino moiety topped with a sulfonyl fluoride at the terminal end (PDB code: 8UZV, Fig. 6a). The electron density map confirms that a single **2f** molecule binds to the active site; however, the sulfonyl fluoride does not form a covalent bond to Y204 and appears to be unreactive in this binding mode. No other binding sites for **2f** were observed. Indeed, the electron density map shows that flexible 8-ethylamino moiety swings away from Y204 resulting in the sulfonyl fluoride being approximately 6.0 Å moved away from the phenolic oxygen of Y204. Therefore, the sulfonyl fluoride is not within appropriate distance to form the covalent bond. The quinolone carboxylic acid head group forms hydrogen bonds to the side chains of K265 and N283 in addition to a water-mediated (Wat 820) hydrogen bond to the backbone carbonyl oxygen of S514. The flexible tail of 8-sulfonylfluoride-ethylamino projects into the DNA binding groove. The quinolone of **2f** superimposes well with the bicyclic quinolone of **2a** (Fig. 4a).

The addition of an 8-phenyl group between the linker 8-ethylamino and quinolone in compounds **2f** (Fig. 6a) resulted in the formation of a covalent bond between quinolone **3b** with the phenolic hydroxyl on Y204 (Fig. 6b). The 1.62 Å resolution 2*F*_o_-*F*_c_ electron density map of TDP1 in complex with **3b** (PDB code: 8V0C) reveals two occupancies of **3b** in the active site (Fig. 6b). We could observe and confirm the covalent attachment of **3b** to Y204. Additional electron density suggests a population of unreacted **3b** present at the active site as well. For the covalently linked **3b**, the quinolone carboxylic acid headgroup forms hydrogen bonds with the side chains of K265 and N283. The oxygen atom of the carbonyl group at the 4-position is involved in a water-mediated (Wat 919) hydrogen bond with the D288 side chain and is also within hydrogen bonding distance to the side chain of H493. For the unreacted **3b**, the observed binding mode shows that the quinolone carboxylic acid headgroup undergoes a slight tilt and forms a direct hydrogen bond with the side chains of N283 and a water-mediated (Wat 801) hydrogen bond to the backbone carbonyl oxygen of S514. The water-mediated hydrogen bonding network between the carbonyl group at the 4-position of the quinolone forms a water-mediated (Wat 919) interaction with D288. The fluorosulfate moiety of the unreacted **3b** is positioned approximately 3.9 Å from the phenolic oxygen atoms of the Y204 side chain and within 3.6 Å of C205. However, we did not observe any electron density to suggest modification of C205.

The 8-phenyl group of **3e** (Fig. 6c) superimposes well with the 8-phenyl group of **3a** (Fig. 4b). However, compound **3e** has not formed any covalent bond with the residues of TDP1 (Fig. 6c). No other binding sites for **3e** were observed. Altering the location of the fluorosulfate from the *para*-position (**3e**) to *ortho*-position (**3c**) on the 8-phenyl ring, results in the formation of the covalent bond of **3c** with the phenolic hydroxyl on Y204 in the crystal structure (Fig. 6d). Fitting of compound **3c** to the electron density shows that the quinolone carboxylic headgroup forms direct hydrogen bonds with the side chains of K265 and N283. The carbonyl oxygen atom at the quinolone 4-position participates in a water-mediated (Wat 895) hydrogen bond network with the side chains of D288 and S459 and is also within hydrogen bonding distance to the side chain of H493.

**Fig. 7:**
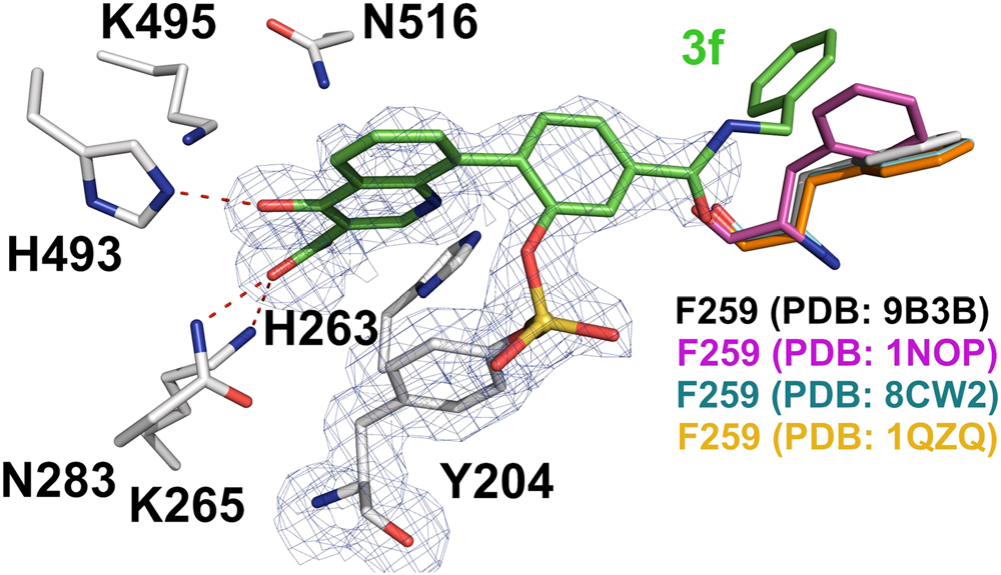
Structure of quinolone 3f covalently bound to TDP1 (PDB code: 9B3B). Structure of TDP1 in complex with compound **3f** (carbon atoms in green). The fit of **3f** to the 2*F*_o_-*F*_c_ electron density map (1.62 Å resolution, blue mesh, contoured at 1.0 σ level) is shown. The position of the terminal phenyl ring of **3f** is modeled. The residue F259 rotamers are highlighted in different colors in the superimposed structures of the TDP1-**3f** model complex (PDB code: 9B3B, F259 in gray) and the coordinates of TDP1 are shown with bound DNA substrate (PDB code: 1NOP, F259 in magenta), without bound substrate (PDB: 1QZQ; F259 in orange) and with an imidazopyridine inhibitor (PDB code: 8CW2; F259 in cyan).

We also collected X-ray diffraction data from TDP1 crystals soaked with compound **3f** (PDB code: 9B3B, Fig. 7). Although, the electron density maps confirm that **3f** binds in the active site via a covalent bond with the side chain of Y204, we did not observe any electron density past the *para*-amide linker which suggests that the phenyl end in **3f** is disordered. We were unable to experimentally determine the binding mode of the full molecule; however, since we observed unambiguous electron density that allowed us to confidently fit the position of the quinolone and aryl scaffold to the electron density as well as the covalent linkage to Y204, we used the parameter files for compound **3f** obtained with the eLBOW utility in Phenix to manually model possible conformation of the extended amide -R moiety with Coot (Fig. 7). In the modeled position of the phenyl ring, the aryl ring of **3f** is within approximately 4.0 Å of the F259 side chain (Fig. 7). Viewing the structure of the TDP1-**3f** model complex superimposed onto the coordinates of TDP1 with bound DNA substrate (PDB code: 1NOP), we observed that main difference is in the conformation of the side chain rotamer of F259 (magenta sticks, PDB code: 1NOP, Fig. 7). Residue F259 (magenta sticks) from DNA substrate bound TDP1 (PDB code: 1NOP) has a T-shape π-π stack pose with the end phenyl of **3f**. Comparing the phenyl poses of residue F259 in DNA substrate bound TDP1 (magenta, PDB code: 1NOP) and our small molecule bound TDP1 (F259 in gray, PDB code: 9B3B) shows a ~ 48° tilt angle shift. The position of F259 in the structure of TDP1 in the absence of ligand (F259 in orange, PDB code: 1QZQ), and of TDP1 in complex with an imidazopyridine inhibitor (F259 in cyan, PDB code: 8CW2) also closely aligns with the observed rotamer of F259 in the TDP1-**3f** complex (F259 in gray, PDB code: 9B3B). The structural difference of F259 between substrate and small molecules might be related to the high substrate barrier^39,40^.

### Mass spectral analysis of sites of protein covalent modification

**Fig. 8:**
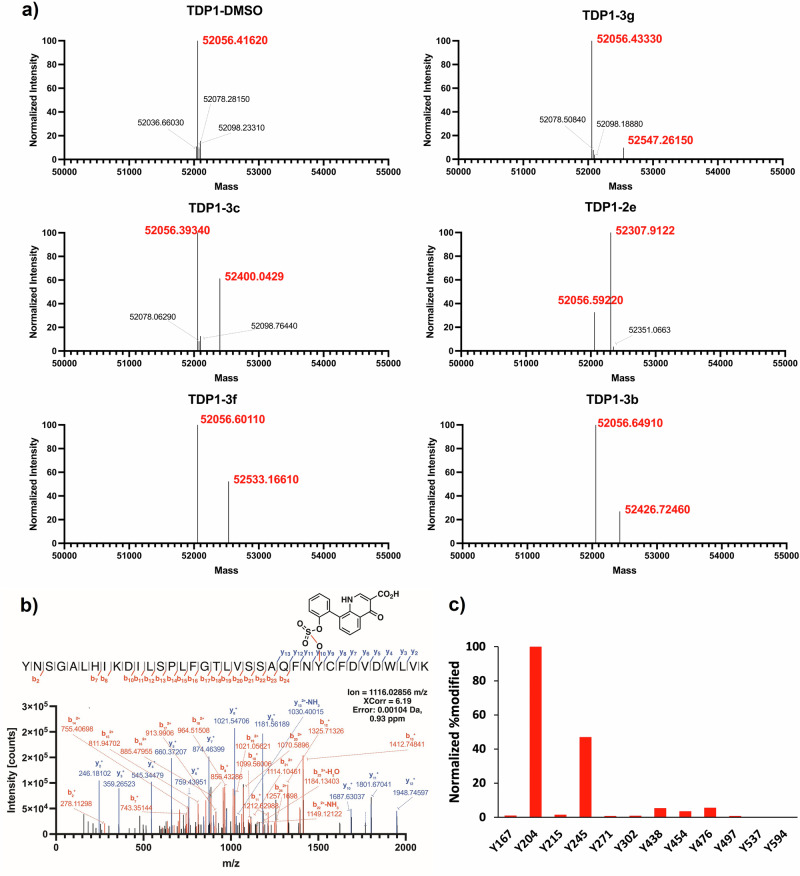
LC-MS analysis of tyrosine modification on TDP1. **a** ReSpect deconvolution of the MS1 scans from TDP1(148-608) incubated with DMSO and the compounds **2e,** **3b,** **3c,** **3f**, and **3g** at a ratio of 1:65 (enzyme/compound) (the significant species shown in red). **b** A representative MS/MS spectrum of covalently modified Y204 with **3c** through the displacement of fluorine. **c** The normalized %modification of **3c** on tyrosine residues of TDP1 calculated from the sum of grouped abundances of the peptides. Y204 harbored the highest grouped abundances based on Label-Free Quantitation (LFQ) values and was used to normalize the %modification.

To confirm TDP1 covalent modification by X-ray crystallography, we examined the intact mass of TDP1(148-608) exposed to select inhibitors (Fig. 8). We incubated the TDP1(148-608) with sulfonylflorides **2e,** **3b**, and fluorosulfates **3c,** **3f**, and **3g** overnight and the resulting reaction mixtures were subjected to intact LC-MS analysis on an Exactive Plus EMR mass spectrometer. The deconvoluted mass of TDP1(148-608) for the DMSO control was determined to be at 52056.41620 Da (Fig. 8a), which deviates 2.06 Da from the expected mass (52058.48 Da). A single covalent modification with the expected loss of fluorine was observed for **2e** (+251.32, calc. +251.22 Da), **3b** (+370.08 Da, calc. +370.38 Da), **3c** (+343.65 Da, calc. +343.31 Da), **3f** (+476.57 Da, calc. +475.46 Da), and **3g** (+490.83 Da, calc. +490.48 Da) as shown in Fig. 8a. At this enzyme to compound ratio, the parent enzyme dominates over the irreversibly covalent modified species. However, increasing the amount of the compound does not result in full conversion to the singly modified protein or generation of multiply modified enzyme. This may reflect poor binding affinity of the ligands, which would be consistent with the relatively low inhibitory potency of these compounds observed in our in vitro assays (Table 1).

**Table 1:** Evaluation of quinolones using a gel-based TDP1 fluorescence assay in vitro


Compound	-R	TDP1 IC_50_ (mM)^*i*^
**1a**	-H	7.2^*ii*^
**2a**	-SO_3_H	4.61 ± 0.35^*iii*^
**2b**	-SO_2_NHPh	--^*iv*^
**2c**	-SO_2_NHCH_2_CH_2_Ph	--^*iv*^
**2d**	-SO_2_CH_2_CH_2_CH_2_Ph	--^*iv*^
**2e**	-SO_2_F	3.98 ± 0.42^*iii*^
**2f**	-NHCH_2_CH_2_SO_2_F	0.56 ± 0.07^*iii*^
**3a**	-Ph	5.17 ± 0.14^*iii*^
**3b**	-Ph-2’-NHCH_2_CH_2_SO_2_F	--^*iv*^
**3c**	-Ph-2’-OSO_2_F	1.99 ± 0.18^*iii*^
**3d**	-Ph-3’-OSO_2_F	0.69 ± 0.04^*iii*^
**3e**	-Ph-4’-OSO_2_F	0.98 ± 0.11^*iii*^
**3f**	-Ph-(2’-OSO_2_F-4’-CONHBn)	0.19 ± 0.04^*iii*^
**3g**	-Ph-(2’-OSO_2_F-4’-CONHCH_2_CH_2_Ph)	0.41 ± 0.07^*iii*^
**3f** (3 hr)^*v*^	-Ph-(2’-OSO_2_F-4’-CONHBn)	0.10 ± 0.02
**3g** (3 hr)^*v*^	-Ph-(2’-OSO_2_F-4’-CONHCH_2_CH_2_Ph)	0.27 ± 0.03
**3f** (5 min., TDP1^Y204F^)^*vi*^	-Ph-(2’-OSO_2_F-4’-CONHBn)	6.10 ± 0.51
**3g** (5 min., TDP1^Y204F^)^*vi*^	-Ph-(2’-OSO_2_F-4’-CONHCH_2_CH_2_Ph)	4.69 ± 1.26

^*i*^ Compounds **2b – f** and **3a – e** were evaluated with two replicates and compounds **3f** and **3g** were evaluated with three replicates in gel-based TDP1 fluorescence assay in vitro (Fig. S1). ^*ii*^Reported data^13^; ^*iii*^Pre-incubation time 5 min with wild-type (WT) TDP1; ^*iv*^No activity up to the highest tested concentration of 9 mM; ^*v*^Pre-incubation time 3 h with WT TDP1; ^*vi*^Pre-incubation time 5 min with mutant TDP1^Y204F^.

As fluorosulfate **3c** was shown to be covalently bound to TDP1 in an X-ray crystal structure (PDB code: 8UZZ, Fig. 6d) and represents a key intermediate for our series of covalent analogs, we employed mass analysis to determine covalent TDP1 modifications by fluorosulfate **3c**. As detailed in the Experimental Section, we incubated compound **3c** with TDP1(148-608) overnight and digested the resulting protein with trypsin followed by LC-MS bottom-up label-free analysis using an orbitrap mass spectrometer. By database searching for **3c** modifications on tyrosine residues, we detected with high confidence modification at the Y204 position (Fig. 8b and Supplementary Data 1). This supports the X-ray crystal structure, indicating that the intentionally targeted active site Y204 was covalently modified by **3c**.

As evidenced by crystal structures, multiple tyrosine residues can be modified by our compounds. Accordingly, we analyzed peptides resulting from protein digestion to identify potential covalent modifications by label-free quantitation proteomics data analysis. We took the sum of the grouped abundances of the peptides containing the **3c**-Y modifications and their **3c**-free counterparts and measured the normalized %modification with respect to the most abundant modification Y204 (Fig. 8c and and Supplementary Data 1). While the active site Y204 was to be the main site of modification, Y245 appeared to be an additional site of covalent modification. This is probably due to the surface-exposed position of Y245, which could make it readily accessible. Over extended exposure, compound **3c** accesses the active site Y204 and forms a thermodynamically stable covalent modification. Since **3c** may be considered to be a prototypical structural representative of the series, these data support the covalent modifications observed from the X-ray crystal structures particularly on Y204.

### Biological evaluation

**Fig. 9:**
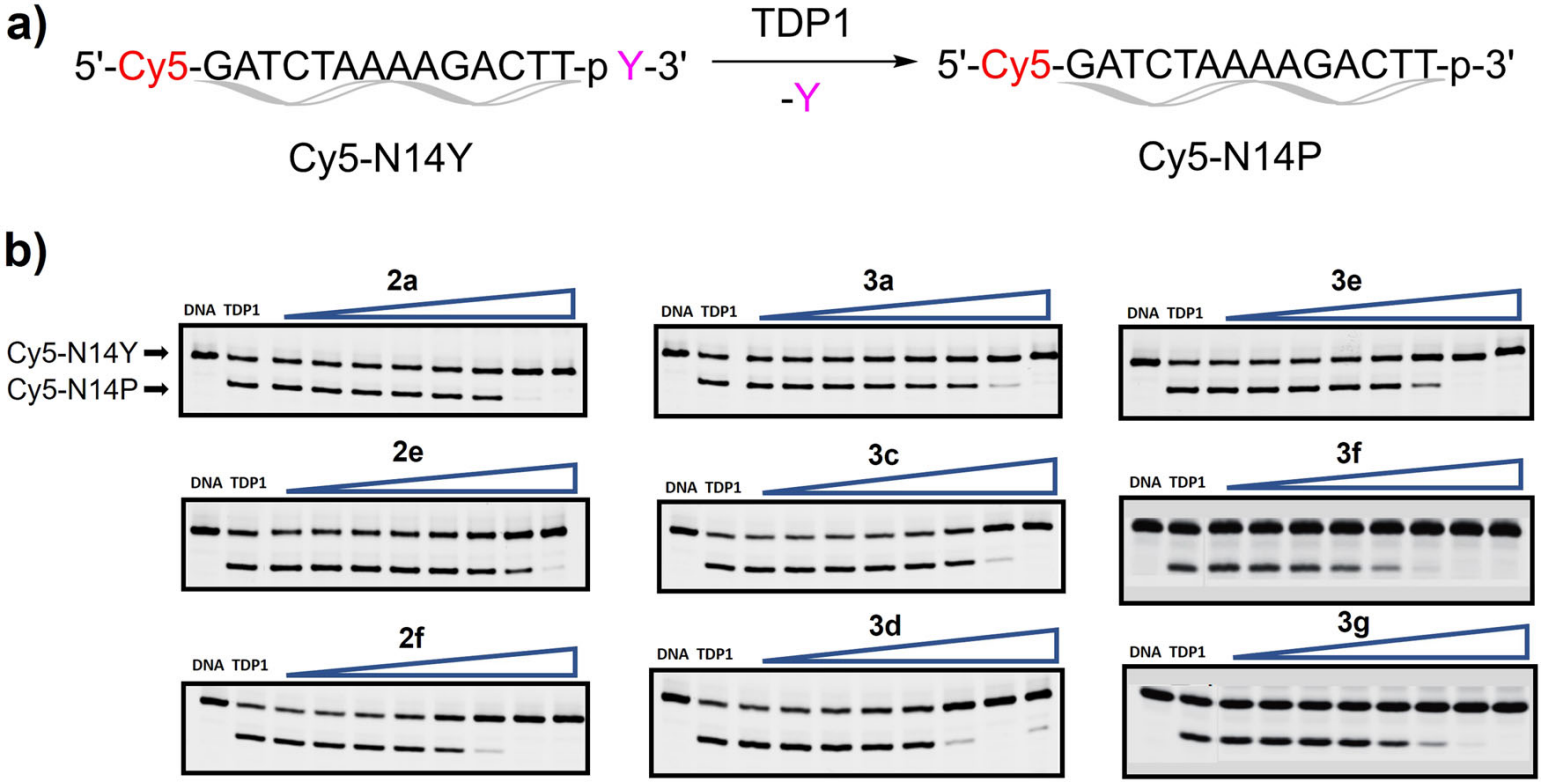
TDP1 catalytic reaction and representative gel images. **a** Scheme of the TDP1 catalytic reaction applied in the gel assays. Cy5-N14Y and Cy5-N14P are the substrate and product of TDP1, respectively. **b** Representative gels showing the inhibition of full-length TDP1-catalyzed hydrolysis by quinolines **2a, 2e, 2f** and **3a, 3c – g**. In each gel: lane 1, DNA (Cy5-N14Y) alone; lane 2, DNA and recombinant TDP1; lanes 3–10, DNA, recombinant TDP1 and the active compounds at 3-fold serial dilution of tested drugs; for **2a** and **3a**: 3-fold serial dilution of drugs from 12 μM to 27 mM; for **2e, 2f, 3c – g**: 3-fold serial dilution of drugs from 4 μM to 9 mM.

We examined the inhibitory effects of the synthetic quinolones in TDP1 catalytic reactions using gel-based analyses (Fig. 9, Table 1, Fig. S1 in Supplementary Information and Supplementary Data 2). In the assay, Cy5-N14Y and Cy5-N14P are the substrate and the product of TDP1 catalysis, respectively (Fig. 9a). Our assays were run in the presence of bovine serum albumin (BSA), which may potentially undergo non-specific covalent modification. However, off-target covalent modification of human serum albumin (HSA) by fluorosulfate-containing compounds has been shown in the literature not to occur^30,41^. Representative gels showing the inhibition of full-length TDP1-catalyzed hydrolysis by quinolines are displayed in Fig. 9b. The TDP1 inhibitory potencies of the quinolones are summarized in Table 1. Quinolone **1a**, lacking an 8-substituent, showed low millimolar inhibitory potency (IC_50_ = 7.2 mM), consistent with to our previous report^13^. Quinolones having 8-substituents showed similar mM inhibitory potencies [**2a** (8-sulfonic acid), IC_50_ = 4.61 mM and **2e** (8-sulfonyl fluoride), IC_50_ = 3.98 mM]. Quinolones **2b – d** bearing 8-sulfonamides do not show inhibition up to the highest tested concentration (9 mM). Quinolone **2f**, which has a sulfonyl fluoride group tethered at the terminus of an 8-ethylamino linker, showed a 13-fold increase in inhibitory potency (IC_50_ = 0.56 mM) as compared with quinolone **1a** (IC_50_ = 7.2 mM). Quinolone **3b** having a sulfonyl fluoride group tethered at the terminus of an 8-ethylaminophenyl chain, did not show inhibition up at the highest tested concentration (9 mM). A quinolone having an 8-phenyl group showed similar inhibitory potency [**3a** (8-phenyl), IC_50_ = 5.17 mM] as the prototype quinolone **1a** (IC_50_ = 7.2 mM). Compounds **3c – e** with a phenyl fluorosulfates at the 8-position retained sub-millimolar inhibitory potencies. The *meta*-substituted 8-phenyloxy fluorophosphate **3d** showed an IC_50_ value of 0.69 mM. In the 8-phenyl series the location of the fluorosulfate group on the phenyl ring affected inhibitory potencies, with the *ortho*-substituted analog **3c** (IC_50_ = 1.99 mM) showing reduced potency relative to the *meta*-fluorosulfate analog **3d** (IC_50_ = 0.69 mM) and the *para*-fluorosulfate **3e** (IC_50_ = 0.98 mM). Addition of *para*-amides onto the 8-phenyl ring of **3c** yielded the phenylmethylamide **3f** and the phenylethylamide **3g**, showed 0.19 mM and 0.41 mM, respectively, and therefore more than 5 to 10-fold increase in inhibitory potency relative to the parent compound **3c** (IC_50_ = 1.99 mM) without the *para*-amides. Compound **3f** displayed a 38-fold increase in inhibitory potency as compared with the starting prototype quinolone **1a** (IC_50_ = 7.2 mM). It is also worth noting that the *para*-phenylethylamino, quinolone **3g** (IC_50_ = 0.41 mM) exhibits a 13-fold increase in TDP1 inhibitory potency compared to quinolone **3a** (IC_50_ = 5.17 mM), which does not contain substituents on 8-phenyl and an 18-fold increase in TDP1 inhibitory potency compared to quinolone **1a** (IC_50_ = 7.2 mM) without substituents on 8-position (Table 1). These may suggest potential π-π interactions formed in the solution phase of the assay condition. It is also worth noting that by extending the pre-incubation time from 5 min to 3 h, the IC_50_ value of compounds **3f** and **3g** were enhanced 1.9-fold and 1.5-fold (0.19 mM vs 0.10 mM for **3f** and 0.41 mM vs 0.27 mM for **3g**), respectively. This time-dependency is consistent with additional covalent bond formation in solution with longer pre-incubation time^42^.

We also investigated **3f** and **3g** using the Y204F TDP1 mutant (TDP1^Y204F^)^23^. Using the mutant TDP1^Y204F^ with 5 min pre-incubation time, compound **3f** shows 32-fold reduced inhibitory potency (0.19 mM vs 6.1 mM for **3f)** and **3g** shows 11-fold reduced inhibitory potency (0.41 mM vs 4.69 mM for **3g**) respectively. Since the TDP1^Y204F^ mutant cannot undergo covalent modification, the loss of inhibitory potency of **3f** and **3g** against the TDP1^Y204F^ supports our crystallographic and proteomic results that covalent modification of Y204 contributes the inhibition in the parent WT enzyme. However, the poor mM inhibitory potencies reflect the low ligand binding affinity and may suggest that covalent modification is not a primary driver of inhibition.

### Implications of the current work

The development of TDP1 inhibitors has been made particularly challenging due to the enzyme topology and mechanisms of catalysis. In the case of TOP1–DNA complexes, the substrate can be viewed as being comprised of three distinct components consisting of the DNA, the TOP1-derived peptide, and a central bridging 3’-ribose ester (Fig. 1a). The enzyme is defined by a relatively shallow central catalytic pocket flanked by open substrate-binding channels that extend in opposite directions to accommodate the peptide and DNA components. The large macromolecular nature of the substrate means that the primary binding energetics occur outside the confined catalytic pocket (Fig. 1b). Interactions of the tyrosyl–DNA substrate within the DNA binding channels must occur in a temporal sequence that precedes catalysis, such that substrate binding affinity and specificity are highly dependent on these “exosite interactions”^43^. Typically, TDP1 inhibitors are small competitive molecules inhibitors designed to bind within the catalytic pocket. However, the extended substrate interactions outside the shallow catalytic pocket make it difficult for such small molecules to achieve effective and enduring inhibition. This represents an example of what has been termed a high “substrate barrier” used to explain the challenge of developing effective Ribonuclease H (RNase H) inhibitors^39,40^.

Recently, we combined small molecule microarray (SMM) and oxime diversification strategies to develop trivalent ligands as TDP1 inhibitors, intended to engage residues within the catalytic pocket, while extending into the DNA and peptide-binding channels^13–15^. The overall affinity of these ligands could theoretically approach the product of the binding affinities of each component of the multivalent constructs. Although some of the resulting trivalent constructs did show micromolar inhibitory values in in vitro assays, the low substrate barriers present in these assays may explain why inhibitory efficacies in whole cell systems were less than what was expected based on results from the in vitro assays^14^. The high substrate barrier of TDP1 complicates the development of inhibitors if the inhibitors must compete with substrate for binding to the enzyme. Development of TDP1 inhibitors is often guided by in vitro assays that do not present the high substrate barrier found in cellular contexts. The substrate barrier could potentially be overcome by inhibitors capable of accommodating the bound substrate by binding to the enzyme-substrate complex (uncompetitive) or by recognizing both the free enzyme and the enzyme-substrate complex (noncompetitive)^44^. After significant effort, a spectrum of structurally diverse TDP1 inhibitors has been discovered^45–54^ including natural product noncompetitive inhibitors of TDP1^54–56^. Recent studies have also shown that the N-terminal domain (NTD) of TDP1 is critical for catalysis in cellular settings and it was postulated that this NTD may be involved with stabilizing the DNA substrate so that it is properly oriented for interaction with the catalytic machinery in the active site^57^. A peptide-based allosteric inhibitor that appears to require the NTD has recently been reported^58^. All of these factors may contribute to the observation that IC_50_ values obtained from in vitro assays may not accurately reflect performance in cellular settings.

As outlined above, there is a need for creative approaches to address problems faced in developing TDP1 inhibitors. Selective protein modification can provide valuable insights into the biological roles of proteins, which can inform ligand design. Using the crystal structures of quinolone-base competitive inhibitors, we identified the Y204 residue within the catalytic pocket as being accessible for selective covalent modification. The focus of our current work is the synthesis and evaluation of site-specific TDP1-directed sulfonyl fluoride and arylfluorosulfate-based affinity ligands designed to target the Y204 residue. We employed SuFEx chemistries to prepare a series of quinolones having sulfonyl fluorides at the 8-position. We then solved the X-ray crystal structures of several covalent TDP1-bound binders showing site-specific covalent bonds with Y204. In parallel, we incubated the TDP1(148-608) with select sulfonylflorides and employed mass analysis to confirm that the intentionally targeted active site Y204 was covalently modified. Our compounds represent the first examples of TDP1-directed affinity covalent ligands. Thus, our work offers promising insights and potential directions for TDP1 modifications and inhibitor development.

## Conclusions

Residues Y204 and F259 in the catalytic groove of TDP1 have been reported to be evolutionarily conserved in different species and characterized biochemically^23^. Our current work starts from our previously identified TDP1-binding quinolone scaffolds^13^ and uses emerging SuFEx click chemistry to prepare a series of quinolones **2a – f** and **3a – g** designed to bind and specifically target the catalytic residues Y204 and F259. This work marks the first example in which SuFEx protocols have been applied to TDP1 binding ligands. We have successfully identified several covalent TDP1 binders that site-specifically form covalent bonds with Y204 based on their high-resolution cocrystal structures and mass spectral analysis. To our knowledge, this work represents the first examples of small molecule ligands that explicitly interact with TDP1 in a covalent fashion. By tethering aryl groups to the end of flexible amide linkers at the *para* position of the 8-phenyl ring, we were able to access and interact with the F259 residue, which serves as a zipper to split dsDNA and direct a DNA single strand to the catalytic pocket. Our crystal structures of TDP1 bound to quinolones reveal that *ortho*-fluorosulfates on the 8-phenyl group are capable of forming covalent bonds with the phenolic hydroxyl of Y204 at the TDP1 catalytic site in a fashion that aligns the *para*-amide toward the DNA-substrate binding groove. Our covalent ligands represent a new genre of pharmacological tools for studying small molecule binding to TDP1. They validate the ability to covalently modify specific TDP1 residues by designed targeting. Adding this capability to the chemical biology toolbox of resources for studying TDP1 could have far-reaching consequences.

## Methods

### In vitro TDP1 gel-based assays

The inhibition of TDP1 or TDP1^Y204F^ was also conducted using a gel-based assay as previously described^13,14^. Briefly, the DNA substrate (1 nM, 5′Cy5-GATCTAAAAGACTT-pY-3′, Cy5-N14Y) was incubated for 15 min. with recombinant full-length TDP1 or TDP1^Y204F^ (40 pM) in the absence or presence of inhibitors for 5 min. or 3 hr preincubation time as shown in Table 1 at room temperature (22 ^o^C) in TDP1 reaction buffer (50 mM Tris HCl, pH 7.5, 80 mM KCl, 2 mM EDTA, 1 mM DTT, 40 µg/mL BSA and 0.01% Tween-20). Reactions were stopped by adding an equal volume of gel loading buffer (99.5% (v/v) formamide, 5 mM EDTA). Samples were then subjected to a 20% denaturing PAGE gel following by gel scanning using a Typhoon FLA 9500 scanner (GE Healthcare). The IC_50_ values of TDP1 inhibitors were calculated by comparing the percentage of cleavage product (5′Cy5-GATCTAAAAGACTT-p-3′, Cy5-N14P) to DMSO control. Compounds **2b – f** and **3a – e** were evaluated with two replicates and compounds **3f** and **3g** were evaluated with three replicates in gel-based TDP1 fluorescence assay (Fig. 9, Fig. S1 in Supplementary Information and Supplementary Data 2).

### X-ray crystallography

The catalytic domain of TDP1 (consisting of residues S148-S608) was expressed and purified for crystallographic studies as previously reported^13^. Crystals were grown by the hanging drop vapor diffusion method by mixing 2 µL of TDP1 (22 mg/mL in 25 mM Tris-HCl pH 7.2, 150 mM sodium chloride, and 2 mM tris(2-carboxyethyl)phosphine buffer) with 2 µL of well solution (0.1 M MOPS/HEPES-Na, pH 7.5, 10% (w/v) PEG 8000, 20% (v/v) ethylene glycol, 0.03 M sodium fluoride, 0.03 M sodium bromide, 0.03 M sodium iodide) and sealed over 500 µL of well solution in a Nextal 15-well crystallization plate. Crystals were improved with streak-seeding. For soaking experiments, stock solutions were prepared in 100% DMSO. Crystals of TDP1 were then transferred to a 4 µL drop solution consisting of well solution supplemented with compounds (concentrations listed in Table S1 in Supplementary Information) in a final concentration of 10% (v/v) DMSO. The drops were then sealed over 500 µL of well solution and the crystals were soaked for 5 days. Crystals for data collection were retrieved from the drops using a litholoop and immediately flash-cooled by plunging into liquid nitrogen without the need of additional cryoprotectant.

X-ray diffraction data were collected remotely at beamlines 22-BM and 22-ID of the SER-CAT facility, Advanced Photon Source, Argonne National Laboratory. For both data sets, X-ray diffraction data were collected with a Rayonix MX300-HS (22-BM) and an Eiger 16 M (22-ID) detector using an X-ray wavelength of 1.0000 Å. The X-ray data sets were processed using HKL3000^59^. The structures were solved by molecular replacement using the coordinates of a previous structure of TDP1 (PDB code: 6DHU)^13^ with all non-protein atoms deleted and the program PHASER^60^ in the Phenix crystallographic software suite^61^. The electron density maps were examined for difference electron density features (*F*_o_-*F*_c_, contoured at 3.0 σ level) to identify the location of the bound compounds. Coordinates for the molecules were prepared using the Molinspiration server (www.molinspiration.com) and the appropriate .cif files for use in refinements were prepared using the eLBOW^62^ feature in Phenix. Iterative rounds of model adjustments and corrections were carried out in Coot^63^ followed by refinement in phenix.refine^64^. Water molecules were located automatically using Coot and phenix.refine, visually inspected, and analyzed with UnDowser^65^ in MolProbity^66^. Final model quality and validation were performed using MolProbity.

Crystallographic data collection and refinement statistics are presented in Table S1 in Supplementary Information. The PDB coordinates and structure factors for the structures of TDP1 bound to **2a** (PDB code: 6DJG), **2e** (PDB code: 8UV1), **2f** (PDB code: 8UZV), **3a** (PDB code: 6MYZ), **3b** (PDB code: 8V0C), **3c** (PDB code: 8UZZ), **3e** (PDB code: 8V0B), **3f** (PDB code: 9B3B) were deposited into the Protein Data Bank under accession codes respectively.

### Intact mass analysis

The recombinant TDP1(148-608) (2 μg) in 50 μL of buffer (25 mM Tris pH 7.5 and 150 mM NaCl) was separately added with DMSO for control or 50 μM of the compounds **2e,** **3b,** **3c,** **3f**, and **3g**. The samples were incubated overnight at 25 ^o^C with shaking at 500 rpm. The samples were diluted with water to 100 μL and analyzed by liquid chromatography coupled online with mass spectrometry (LC-MS)^67^. Separation was performed on a Vanquish Flex chromatographic system (Thermo) using a MabPac reversed-phase column (4 μm, 1500 Å, 3 ×50 mm, Thermo) maintained at 50 ^o^C. The labeled proteins were resolved at 0.5 mL/min over a 15 min gradient of buffer B (47.5% acetonitrile, 47.5% isopropanol, 5% water, 0.2% formic acid (FA)) from 2 to 100% followed by re-equilibration at 2% buffer B (total run time 20 min) against buffer A (5% acetonitrile, 0.2% FA in water). The eluates from the column were introduced to an Exactive Plus EMR mass spectrometer (Thermo) through a heated electrospray ionization (HESI) source. Intact mass spectra were acquired over 500-2000 m/z window at 35,000 FT resolution (at 200 m/z), averaging 10 microscans, with an AGC target of 3 ×10^6^, max injection time of 200 ms, 80% S-lens RF level, 20 V source-induced dissociation, and capillary temperature set at 275 ^o^C. The resulting spectra were manually averaged followed by deconvolution using the ReSpect algorithm in BioPharma Finder 2.0 (Thermo). The default deconvolution parameters used were 20 ppm deconvolution mass tolerance, 6-10 minimum adjacent charges (low and high model mass), 0% relative abundance threshold, 2:2 left/right peak shape, peak detection minimum significance measure of 1 standard deviation, 95% peak detection quality measure, peak model width factor of 1, 0.01 intensity threshold scale, and the noise compensation set to true.

### Mapping of 3c modification on TDP1

Mapping the site of modification on TDP1 was performed as described previously^68^. The recombinant TDP1(148-608) (2 μg) in 100 μL of buffer (25 mM Tris pH 7.5 and 150 mM NaCl) was added with DMSO or 400 μM of **3c** and prepared in triplicates, followed by overnight incubation at 25 ^o^C with shaking at 500 rpm. The samples were then each treated with 100 μL HEPES pH 8, 50 μL, EasyPep lysis buffer, 50 μL reducing solution, and 50 μL alkylating solution provided in the EasyPep kit (Thermo A45733) along with 2 μg Trypsin-LysC (Thermo A40007) and incubated overnight at 37 ^o^C with shaking at 500 rpm. The samples were acidified with 50 μL of 20% FA and cleaned up using the EasyPep MS sample prep 96-well plate (Thermo A57865) by following the manufacturer’s protocol. The eluted peptides were dried under reduced pressure.

The peptides were reconstituted in 50 μL of 3% acetonitrile + 0.1% formic acid (FA) in water and 7 μL was analyzed on a Dionex U3000 RSLC attached to an Orbitrap Eclipse (Thermo) equipped with an EasySpray ion source. The LC unit used solvent A consisted of 0.1% FA in water and solvent B consisted of 0.1% FA + 80% acetonitrile in water. The peptides were separated in an analytical column EasySpray C18 HPLC column (2 μm, 75 μm i.d., 25 cm, PN ES902) using the following gradient operated at 300 nL/min: 5-7% B for 1 min, 7-30% B for 34 min, 30-50% B for 15 min, 50-95% B for min, holding at 95% B for 7 min, then re-equilibration at 5%B for 13 min. The MS injections used the TopSpeed method set at 3 s cycle time operated with the following: Spray voltage at 1800 V and ion transfer temperature of 275 ^o^C. The MS1 scans were acquired in the Orbitrap with a resolution of 120,000, AGC target of 4 ×10^5^, max injection time of 50 ms, while the MS2 scans were acquired in the Orbitrap as well with 15,000 resolution, 5 ×10^4^ of AGC target, 22 ms of max injection time, 30% HCD energy, 1.6 Da isolation window, 2.5 ×10^4^ intensity threshold for MS selection. The charge state selection and mass range were set to 2-5 and 400-1600 m/z, respectively.

The .raw files were searched in Proteome Discoverer 2.4 using Sequest against the truncated sequence of Human TDP1 (UniProt ID Q9NUW8 148-608 aa) using a full tryptic digest, 2 max missed cleavages, minimum of 6 and maximum of 40 amino acids, 10 ppm MS1 tolerance, 0.02 Da MS2 mass tolerance, fixed modification for cysteine carbamidomethylation ( + 57.021 Da) and variable modifications of oxidation ( + 15.995 Da) on methionine and **3c** covalent modification ( + 343.015 Da) on tyrosine. Percolator was used for FDR analysis and IMP-ptmRS for site localization. Parent ion intensities were used to measure label-free quantitation values (LFQ) reported as abundances and the sum of the grouped abundances was used to estimate the %modification of **3c** on tyrosine residues.

### Reporting summary

Further information on research design is available in the Nature Portfolio Reporting Summary linked to this article.

## Supplementary information

Supplementary Information

Description of Additional Supplementary Files

Supplementary Data 1

Supplementary Data 2

Reporting Summary

## Data Availability

The authors declare that the data supporting the study are available within the article and Supplementary Information. For experimental details and methods, see Supplementary Information. Mass spectrometry data files are available on MassIVE (Accession: MSV000095388).

## References

[1] Barthelmes, H. U. et al. TDP1 overexpression in human cells counteracts DNA damage mediated by topoisomerases I and II. *J. Biol. Chem.* **279**, 55618–55625 (2004). 15494395 10.1074/jbc.M405042200

[2] Beretta, G. L., Cossa, G., Gatti, L., Zunino, F. & Perego, P. Tyrosyl-DNA phosphodiesterase 1 targeting for modulation of camptothecin-based treatment. *Curr. Med. Chem.* **17**, 1500–1508 (2010). 20166932 10.2174/092986710790979971

[3] Pommier, Y. et al. Tyrosyl-DNA-phosphodiesterases (TDP1 and TDP2). *DNA Repair (Amst.)* **19**, 114–129 (2014). 24856239 10.1016/j.dnarep.2014.03.020 PMC4090310

[4] Huang, S. N. & Pommier, Y. Mammalian tyrosyl-DNA phosphodiesterases in the context of mitochondrial DNA repair. *Int. J. Mol. Sci.* **20**, 3015 (2019). 31226795 10.3390/ijms20123015 PMC6628236

[5] Pouliot, J. J., Yao, K. C., Robertson, C. A. & Nash, H. A. Yeast gene for a Tyr-DNA phosphodiesterase that repairs topoisomerase I complexes. *Science* **286**, 552–555 (1999). 10521354 10.1126/science.286.5439.552

[6] Interthal, H., Pouliot, J. J. & Champoux, J. J. The tyrosyl-DNA phosphodiesterase Tdp1 is a member of the phospholipase D superfamily. *Proc. Natl Acad. Sci. USA* **98**, 12009–12014 (2001). 11572945 10.1073/pnas.211429198 PMC59758

[7] Murai, J. et al. Tyrosyl-DNA phosphodiesterase 1 (TDP1) repairs DNA damage induced by topoisomerases I and II and base alkylation in vertebrate cells. *J. Biol. Chem.* **287**, 12848–12857 (2012). 22375014 10.1074/jbc.M111.333963 PMC3339927

[8] Interthal, H. et al. SCAN1 mutant Tdp1 accumulates the enzyme-DNA intermediate and causes camptothecin hypersensitivity. *EMBO J.* **24**, 2224–2233 (2005). 15920477 10.1038/sj.emboj.7600694 PMC1150888

[9] Plo, I. et al. Association of XRCC1 and tyrosyl DNA phosphodiesterase (Tdp1) for the repair of topoisomerase I-mediated DNA lesions. *DNA Repair (Amst.)* **2**, 1087–1100 (2003). 13679147 10.1016/S1568-7864(03)00116-2

[10] Comeaux, E. Q. & van Waardenburg, R. C. A. M. Tyrosyl-DNA phosphodiesterase I resolves both naturally and chemically induced DNA adducts and its potential as a therapeutic target. *Drug Metab. Rev.* **46**, 494–507 (2014). 25327705 10.3109/03602532.2014.971957

[11] Gao, R. et al. Epigenetic and genetic inactivation of tyrosyl-DNA-phosphodiesterase 1 (TDP1) in human lung cancer cells from the NCI-60 panel. *DNA Repair* **13**, 1–9 (2014). 24355542 10.1016/j.dnarep.2013.09.001 PMC3919147

[12] Flett, F. J. et al. Structural basis for DNA 3’-end processing by human tyrosyl-DNA phosphodiesterase 1. *Nat. Commun.* **9**, 1–13 (2018). 29295983 10.1038/s41467-017-02530-z PMC5750209

[13] Lountos, G. T. et al. Identification of a ligand binding hot spot and structural motifs replicating aspects of tyrosyl-DNA phosphodiesterase I (TDP1) phosphoryl recognition by crystallographic fragment cocktail screening. *Nucleic Acids Res* **47**, 10134–10150 (2019). 31199869 10.1093/nar/gkz515 PMC6821334

[14] Zhao, X. Z. et al. Small molecule microarray identifies inhibitors of tyrosyl-DNA phosphodiesterase 1 that simultaneously access the catalytic pocket and two substrate binding sites. *Chem. Sci.* **12**, 3876–3884 (2021). 34163656 10.1039/D0SC05411A PMC8179437

[15] Zhao, X. Z. et al. Identification of multidentate tyrosyl-DNA phosphodiesterase 1 (TDP1) inhibitors that simultaneously access the DNA, protein and catalytic-binding sites by oxime diversification. *RSC Chem. Biol.* **4**, 334–343 (2023). 37181631 10.1039/D2CB00230B PMC10170656

[16] Zhao, X. Z. et al. Phosphonic acid-containing inhibitors of tyrosyl-DNA phosphodiesterase 1. *Front. Chem.* **10**, 910953 (2022). 36051621 10.3389/fchem.2022.910953 PMC9424690

[17] Davies, D. R., Interthal, H., Champoux, J. J. & Hol, W. G. J. The crystal structure of human tyrosyl-DNA phosphodiesterase, Tdp1. *Structure* **10**, 237–248 (2002). 11839309 10.1016/S0969-2126(02)00707-4

[18] Davies, D. R. & Hol, W. G. J. The power of vanadate in crystallographic investigations of phosphoryl transfer enzymes. *FEBS Lett.* **577**, 315–321 (2004). 15556602 10.1016/j.febslet.2004.10.022

[19] Raymond, A. C., Rideout, M. C., Staker, B., Hjerrild, K. & Burgin, A. B. Analysis of human tyrosyl-DNA phosphodiesterase I catalytic residues. *J. Mol. Biol.* **338**, 895–906 (2004). 15111055 10.1016/j.jmb.2004.03.013

[20] Debéthune, L., Kohlhagen, G., Grandas, A. & Pommier, Y. Processing of nucleopeptides mimicking the topoisomerase I–DNA covalent complex by tyrosyl-DNA phosphodiesterase. *Nucleic Acids Res* **30**, 1198–1204 (2002). 11861912 10.1093/nar/30.5.1198 PMC101246

[21] Sun, Y., Saha, L. K., Saha, S., Jo, U. & Pommier, Y. Debulking of topoisomerase DNA-protein crosslinks (TOP-DPC) by the proteasome, non-proteasomal and non-proteolytic pathways. *DNA Repair (Amst.)* **94**, 102926 (2020). 32674013 10.1016/j.dnarep.2020.102926 PMC9210512

[22] Sun, Y. et al. Excision repair of topoisomerase DNA-protein crosslinks (TOP-DPC). *DNA Repair (Amst.)* **89**, 102837 (2020). 32200233 10.1016/j.dnarep.2020.102837 PMC7188568

[23] Kiselev, E., Dexheimer, T. S., Marchand, C., Huang, S.-Y. N. & Pommier, Y. Probing the evolutionary conserved residues Y204, F259, S400 and W590 that shape the catalytic groove of human TDP1 for 3’- and 5’-phosphodiester-DNA bond cleavage. *DNA Repair* **66-67**, 64–71 (2018). 29747024 10.1016/j.dnarep.2018.05.001 PMC8057126

[24] Dexheimer, S. T., Antony, S., Marchand, C. & Pommier, Y. Tyrosyl-DNA phosphodiesterase as a target for anticancer therapy. *Anti-Cancer Agents Med. Chem.* **8**, 381–389 (2008). 10.2174/187152008784220357 PMC2443942 18473723

[25] Dong, J., Krasnova, L., Finn, M. G. & Sharpless, K. B. Sulfur(VI) fluoride exchange (SuFEx): another good reaction for click chemistry. *Angew. Chem. Int. Ed.* **53**, 9430–9448 (2014). 10.1002/anie.201309399 25112519

[26] Hett, E. C. et al. Rational targeting of active-site tyrosine residues using sulfonyl fluoride probes. *ACS Chem. Biol.* **10**, 1094–1098 (2015). 25571984 10.1021/cb5009475

[27] Chen, W. et al. Arylfluorosulfates inactivate intracellular lipid binding protein(s) through chemoselective sufex reaction with a binding site Tyr residue. *J. Am. Chem. Soc.* **138**, 7353–7364 (2016). 27191344 10.1021/jacs.6b02960 PMC4909538

[28] Kitamura, S. et al. Sulfur(VI) fluoride exchange (SuFEx)-enabled high-throughput medicinal chemistry. *J. Am. Chem. Soc.* **142**, 10899–10904 (2020). 32479075 10.1021/jacs.9b13652 PMC7751259

[29] Lou, T. S.-B. & Willis, M. C. Sulfonyl fluorides as targets and substrates in the development of new synthetic methods. *Nat. Rev. Chem.* **6**, 146–162 (2022). 37117299 10.1038/s41570-021-00352-8

[30] Martin-Gago, P. & Olsen, C. A. Arylfluorosulfate-based electrophiles for covalent protein labeling: A new addition to the arsenal. *Angew. Chem. Int. Ed.* **58**, 957–966 (2019). 10.1002/anie.201806037 PMC6518939 30024079

[31] Elsea, S. H., Osheroff, N. & Nitiss, J. L. Cytotoxicity of quinolones toward eukaryotic cells. Identification of topoisomerase II as the primary cellular target for the quinolone CP-115,953 in yeast. *J. Biol. Chem.* **267**, 13150–13153 (1992). 1320012 10.1016/S0021-9258(18)42185-0

[32] Hooper, D. C. Emerging mechanisms of fluoroquinolone resistance. *Emerg. Infect. Dis.* **7**, 337–341 (2001). 11294736 10.3201/eid0702.010239 PMC2631735

[33] Andriole, V. T. The quinolones: past, present, and future. *Clin. Infect. Dis.* **41**, S113–S119 (2005). 15942877 10.1086/428051

[34] Foti, J. J., Devadoss, B., Winkler, J. A., Collins, J. J. & Walker, G. C. Oxidation of the guanine nucleotide pool underlies cell death by bactericidal antibiotics. *Science* **336**, 315–319 (2012). 22517853 10.1126/science.1219192 PMC3357493

[35] Aldred, K. J., Kerns, R. J. & Osheroff, N. Mechanism of quinolone action and resistance. *Biochem* **53**, 1565–1574 (2014). 24576155 10.1021/bi5000564 PMC3985860

[36] Pham, T. D. M., Ziora, Z. M. & Blaskovich, M. A. T. Quinolone antibiotics. *Medchemcomm* **10**, 1719–1739 (2019). 31803393 10.1039/C9MD00120D PMC6836748

[37] Terreni, M., Taccani, M. & Pregnolato, M. New antibiotics for multidrug-resistant bacterial strains: latest research developments and future perspectives. *Molecules* **26**. 10.3390/molecules26092671 (2021). 10.3390/molecules26092671 PMC8125338 34063264

[38] Miyaura, N. & Suzuki, A. Palladium-catalyzed cross-coupling reactions of organoboron compounds. *Chem. Rev.* **95**, 2457–2483 (1995). 10.1021/cr00039a007

[39] Wang, L., Sarafianos, S. G. & Wang, Z. Cutting into the substrate dominance: pharmacophore and structure-based approaches toward inhibiting human immunodeficiency virus reverse transcriptase-associated ribonuclease H. *Acc. Chem. Res.* **53**, 218–230 (2020). 31880912 10.1021/acs.accounts.9b00450 PMC7144833

[40] Madia, V. N. et al. Small-molecule inhibitors of HIV-1 reverse transcriptase-associated ribonuclease H function: Challenges and recent developments. *Curr. Med. Chem.* **28**, 6146–6178 (2021). 34225606 10.2174/0929867328666210322164557

[41] Fadeyi, O. O. et al. Covalent enzyme inhibition through fluorosulfate modification of a noncatalytic serine residue. *ACS Chem. Biol.* **12**, 2015–2020 (2017). 28718624 10.1021/acschembio.7b00403

[42] Bolding, J. E. et al. Aryl fluorosulfate based inhibitors that covalently target the SIRT5 lysine deacylase. *Angew. Chem. Int. Ed.* **61**, e202204565 (2022). 10.1002/anie.202204565 PMC9828517 36130196

[43] Copeland, R. A. *Evaluation of Enzyme Inhibitors in Drug Discovery*, 2nd Edition. 2nd Edition edn, (John Wiley & Sons, Inc.). 10.1002/9781118540398 2013.

[44] Buker, S. M., Boriack-Sjodin, P. A. & Copeland, R. A. Enzyme-inhibitor interactions and a simple, rapid method for determining inhibition modality. *SLAS Discov.* **24**, 515–522 (2019). 30811960 10.1177/2472555219829898

[45] Zakharenko, A., Dyrkheeva, N. & Lavrik, O. Dual DNA topoisomerase 1 and tyrosyl-DNA phosphodiesterase 1 inhibition for improved anticancer activity. *Med. Res. Rev.* **39**, 1427–1441 (2019). 31004352 10.1002/med.21587

[46] Koldysheva, E. V. et al. Antimetastatic activity of combined topotecan and tyrosyl-DNA phosphodiesterase-1 inhibitor on modeled lewis lung carcinoma. *Bull. Exp. Biol. Med.* **166**, 661–666 (2019). 30903487 10.1007/s10517-019-04413-3

[47] Zakharenko, A. L. et al. Novel tyrosyl-DNA phosphodiesterase 1 inhibitors enhance the therapeutic impact of topoteсan on in vivo tumor models. *Eur. J. Med. Chem.* **161**, 581–593 (2019). 10.1016/j.ejmech.2018.10.055 30396105

[48] Luzina, O. et al. Usnic acid conjugates with monoterpenoids as potent tyrosyl-DNA phosphodiesterase 1 inhibitors. *J. Nat. Prod.* **83**, 2320–2329 (2020). 32786885 10.1021/acs.jnatprod.9b01089

[49] Zakharenko, A. L. et al. Inhibition of tyrosyl-DNA phosphodiesterase 1 by lipophilic pyrimidine nucleosides. *Molecules* **25**, 3694 (2020). 32823658 10.3390/molecules25163694 PMC7465190

[50] Khomenko, T. M. et al. Promising new inhibitors of tyrosyl-DNA phosphodiesterase i (TDP 1) combining 4-arylcoumarin and monoterpenoid moieties as components of complex antitumor therapy. *Int. J. Mol. Sci.* **21**, 126 (2020). 10.3390/ijms21010126 PMC6982354 31878088

[51] Gladkova, E. D. et al. The first berberine-based inhibitors of tyrosyl-DNA phosphodiesterase 1 (Tdp1), an important dna repair enzyme. *Int. J. Mol. Sci.* **21**, 7162 (2020). 32998385 10.3390/ijms21197162 PMC7582571

[52] Salomatina, O. V. et al. Deoxycholic acid as a molecular scaffold for tyrosyl-DNA phosphodiesterase 1 inhibition: A synthesis, structure-activity relationship and molecular modeling study. *Steroids* **165**, 108771 (2021). 33221302 10.1016/j.steroids.2020.108771

[53] Dyrkheeva, N. S. et al. New hybrid compounds combining fragments of usnic acid and monoterpenoids for effective tyrosyl-DNA phosphodiesterase 1 inhibition. *Biomol* **11**, 973 (2021). 10.3390/biom11070973 PMC8301776 34356597

[54] Zakharenko, A. L. et al. Natural products and their derivatives as inhibitors of the DNA repair enzyme tyrosyl-DNA phosphodiesterase 1. *Int. J. Mol. Sci.* **24**, 5781 (2023). 36982848 10.3390/ijms24065781 PMC10051138

[55] Bermingham, A. et al. Identification of natural products that inhibit the catalytic function of human tyrosyl-DNA phosphodiesterase (TDP1). *SLAS Discov.* **22**, 1093–1105 (2017). 28697309 10.1177/2472555217717200 PMC7900901

[56] Wei, X. et al. Pyranodipyran derivatives with tyrosyl DNA phosphodiesterase 1 inhibitory activities and fluorescent properties from aspergillus sp. EGF 15-0-3. *Mar. Drugs* **20**, 211 (2022). 35323510 10.3390/md20030211 PMC8954640

[57] Brettrager, E. J. et al. N-terminal domain of tyrosyl-DNA phosphodiesterase I regulates topoisomerase I-induced toxicity in cells. *Sci. Rep.* **13**, 1377 (2023). 36697463 10.1038/s41598-023-28564-6 PMC9876888

[58] Krumpe, L. R. H. et al. Recifin A, initial example of the Tyr-lock peptide structural family, is a selective allosteric inhibitor of tyrosyl-DNA phosphodiesterase I. *J. Am. Chem. Soc.* **142**, 21178–21188 (2020). 33263997 10.1021/jacs.0c10418 PMC8921975

[59] Minor, W., Cymborowski, M., Otwinowski, Z. & Chruszcz, M. HKL-3000: the integration of data reduction and structure solution - from diffraction images to an initial model in minutes. *Acta Crystallogr. D. Biol. Crystallogr.* **62**, 859–866 (2006). 16855301 10.1107/S0907444906019949

[60] McCoy, A. J. et al. Phaser crystallographic software. *J. Appl. Crystallogr.* **40**, 658–674 (2007). 19461840 10.1107/S0021889807021206 PMC2483472

[61] Liebschner, D. et al. Macromolecular structure determination using X-rays, neutrons and electrons: recent developments in Phenix. *Acta Crystallogr. D. Struct. Biol.* **75**, 861–877 (2019). 31588918 10.1107/S2059798319011471 PMC6778852

[62] Moriarty, N. W., Grosse-Kunstleve, R. W. & Adams, P. D. electronic Ligand Builder and Optimization Workbench (eLBOW): a tool for ligand coordinate and restraint generation. *Acta Crystallogr. D. Biol. Crystallogr.* **65**, 1074–1080 (2009). 19770504 10.1107/S0907444909029436 PMC2748967

[63] Emsley, P., Lohkamp, B., Scott, W. G. & Cowtan, K. Features and development of Coot. *Acta Crystallogr. D. Biol. Crystallogr.* **66**, 486–501 (2010). 20383002 10.1107/S0907444910007493 PMC2852313

[64] Afonine, P. V. et al. Towards automated crystallographic structure refinement with phenix.refine. *Acta Crystallogr. D. Biol. Crystallogr.* **68**, 352–367 (2012). 22505256 10.1107/S0907444912001308 PMC3322595

[65] Prisant, M. G., Williams, C. J., Chen, V. B., Richardson, J. S. & Richardson, D. C. New tools in MolProbity validation: CaBLAM for CryoEM backbone, UnDowser to rethink “waters,” and NGL Viewer to recapture online 3D graphics. *Protein Sci.* **29**, 315–329 (2020). 31724275 10.1002/pro.3786 PMC6933861

[66] Chen, V. B. et al. MolProbity: all-atom structure validation for macromolecular crystallography. *Acta Crystallogr. D Biol. Crystallogr.* **66**, 12–21 (2010). 20057044 10.1107/S0907444909042073 PMC2803126

[67] D’Ippolito, R. A. et al. Refining the N-termini of the SARS-CoV-2 spike protein and its discrete receptor-binding domain. *J. Proteome Res.* **20**, 4427–4434 (2021). 34379411 10.1021/acs.jproteome.1c00349 PMC8419861

[68] Kim, H.-R. et al. Discovery of a tunable heterocyclic electrophile 4-chloro-pyrazolopyridine that defines a unique subset of ligandable cysteines. *ACS Chem. Biol.* **19**, 1082–1092 (2024). 38629450 10.1021/acschembio.4c00025 PMC11107811

