# Supplementary material for: Targeted sulfur(VI) fluoride exchange-mediated covalent modification of a tyrosine residue in the catalytic pocket of tyrosyl-DNA phosphodiesterase 1
Source: Commun Chem. 2024 Sep 16;7:208. doi: 10.1038/s42004-024-01298-w (PMC11405833; doi:10.1038/s42004-024-01298-w)
Supplement: Supplementary file 1 — Supplementary Information [file 42004_2024_1298_MOESM1_ESM.pdf]

Supplementary Information

Targeted sulfur(VI) fluoride exchange-mediated covalent modification of a tyrosine residue in the catalytic pocket of tyrosyl-DNA phosphodiesterase 1

Xue Zhi Zhao[1*], Idris A. Barakat[1], George T. Lountos[2], Wenjie Wang[3], Keli Agama[3], Md Rasel Al Mahmud[3], Kiall F. Suazo[1,4], Thorkell Andresson[4], Yves Pommier[3], and Terrence R. Burke, Jr.[1]

[1]Chemical Biology Laboratory, Center for Cancer Research, National Cancer Institute, National Institutes of Health, Frederick, MD, USA.

[2]Basic Science Program, Frederick National Laboratory for Cancer Research, Frederick, MD, USA.

[3]Developmental Therapeutics Branch & Laboratory of Molecular Pharmacology, Center for Cancer Research, National Cancer Institute, National Institutes of Health, Bethesda, MD, USA.

[4]Protein Characterization Laboratory, Cancer Research Technology Program, Frederick National Laboratory for Cancer Research, Frederick, MD, USA.

Corresponding author[*]: xuezhi.zhao@nih.gov

Table of Content

Contents **Page**

I. SUPPLEMENTARY METHODS S6

1.1 Synthesis of Substituted Quinolones **2a – f** S6

1.2 Synthesis of Substituted Quinolones **3a** and **3b** S7

1.3 Synthesis of Quinolones **3c – e** S8

1.4 Synthesis of Quinolones **3f** and **3g** S9

SCHEMES

Scheme S1. Preparation of substituted quinolones **2a – f** S7

Scheme S2. Preparation of substituted quinolones **3a** and **3b** S8

Scheme S3. Preparation of quinolones **3c – e** S9

Scheme S4. Preparation of quinolones **3f** and **3g** S10

II. EXPERIMENTAL SECTION S10

General Synthetic Procedures S10

2.1 General Procedure A. Preparation of the substituted 4-oxo-1,4-dihydroquinoline-3-carboxylic acids (**2a – e**) S11

2.2 General Procedure B. Preparation of fluorosulfates (**16a – c, 22a, b**) using SuFEx click chemistry S11

2.3 General Procedure C. Preparation of phenols (**15a – c**) using Suzuki coupling S11

2.4 General Procedure D. Preparation of acids (**3c – g**) S12

2.5 General Procedure E. Preparation of amides (**19a, b**) S12

2.6 General Procedure F. Preparation of boronate esters (**20a, b**) and quinolones (**21a, b**) using Miyaura-Borylation reaction.. S12

III. COMPOUND PREPARATION S13

3.1 Preparation of *8-(chlorosulfonyl)-4-oxo-1,4-dihydroquinoline-3-carboxylic acid (**5**)* S13

3.2 Preparation of *4-oxo-8-sulfo-1,4-dihydroquinoline-3-carboxylic acid (**2a**)*............ S14

3.3 Preparation of *4-oxo-8-(N-phenylsulfamoyl)-1,4-dihydroquinoline-3-carboxylic acid (**2b**)* S14

3.4 Preparation of *4-oxo-8-(N-phenethylsulfamoyl)-1,4-dihydroquinoline-3-carboxylic acid (**2c**)* S14

3.5 Preparation of *4-oxo-8-(N-(3-phenylpropyl)sulfamoyl)-1,4-dihydroquinoline-3-carboxylic acid (**2d**)*.. S15

3.6 Preparation of *8-(fluorosulfonyl)-4-oxo-1,4-dihydroquinoline-3-carboxylic acid (**2e**)* S16

3.7 Preparation of *8-((2-(fluorosulfonyl)ethyl)amino)-4-oxo-1,4-dihydroquinoline-3-carboxylic acid (**2f**)*.. S16

3.8 Preparation of *diethyl 2-(([1,1'-biphenyl]-2-ylamino)methylene)malonate (**9**)*........ S17

3.9 Preparation of *ethyl 4-oxo-8-phenyl-1,4-dihydroquinoline-3-carboxylate (**10**)*....... S17

3.10 Preparation of *4-oxo-8-phenyl-1,4-dihydroquinoline-3-carboxylic acid (**3a**)*........ S18

3.11 Preparation of *8-(2-aminophenyl)-4-oxo-1,4-dihydroquinoline-3-carboxylic acid (**12**)* S18

3.12 Preparation of *8-(2-((2-(fluorosulfonyl)ethyl)amino)phenyl)-4-oxo-1,4-dihydroquinoline-3-carboxylic acid (**3b**)* S19

3.13 Preparation of *tert-butyl 8-bromo-4-oxo-1,4-dihydroquinoline-3-carboxylate* ***(13)*** S20

3.14 Preparation of *tert-butyl 8-(2-hydroxyphenyl)-4-oxo-1,4-dihydroquinoline-3-carboxylate* ***(15a)*** S21

3.15 Preparation of *tert-butyl 8-(3-((fluorosulfonyl)oxy)phenyl)-4-oxo-1,4-dihydroquinoline-3-carboxylate* ***(15b)*** S21

3.16 Preparation of *tert-butyl 8-(4-hydroxyphenyl)-4-oxo-1,4-dihydroquinoline-3-carboxylate* ***(16c)*** S22

3.17 Preparation of *tert-butyl 8-(2-((fluorosulfonyl)oxy)phenyl)-4-oxo-1,4-dihydroquinoline-3-carboxylate* ***(16a)*** S22

3.18 Preparation of *tert-butyl 8-(3-((fluorosulfonyl)oxy)phenyl)-4-oxo-1,4-dihydroquinoline-3-carboxylate* ***(16b)*** S22

3.19 Preparation of *tert-butyl 8-(4-((fluorosulfonyl)oxy)phenyl)-4-oxo-1,4-dihydroquinoline-3-carboxylate* ***(16c)*** S23

3.20 Preparation of *8-(4-((fluorosulfonyl)oxy)phenyl)-4-oxo-1,4-dihydroquinoline-3-carboxylic acid* ***(3c)*** S23

3.21 Preparation of *8-(3-((fluorosulfonyl)oxy)phenyl)-4-oxo-1,4-dihydroquinoline-3-carboxylic acid* ***(3d)*** S24

3.22 Preparation of *8-(2-((fluorosulfonyl)oxy)phenyl)-4-oxo-1,4-dihydroquinoline-3-carboxylic acid* ***(3e)*** S24

3.23 Preparation of *N-benzyl-4-bromo-3-hydroxybenzamide* ***(19a)*** S25

3.24 Preparation of *4-bromo-3-hydroxy-N-phenethylbenzamide* ***(19b)*** S25

3.25 Preparation of *tert-butyl 8-(4-(benzylcarbamoyl)-2-hydroxyphenyl)-4-oxo-1,4-dihydroquinoline-3-carboxylate (**21a**)* S26

3.26 Preparation of *tert-butyl 8-(2-hydroxy-4-(phenethylcarbamoyl)phenyl)-4-oxo-1,4-dihydroquinoline-3-carboxylate (**21b**)* S26

3.27 Preparation of *tert-butyl-8-(4-(benzylcarbamoyl)-2-((fluorosulfonyl)oxy)phenyl)-4-oxo-1,4-dihydroquinoline-3-carb-oxylate (**22a**)* S26

3.28 Preparation of *tert-butyl-8-(2-((fluorosulfonyl)oxy)-4-(phenethylcarbamoyl)phenyl)-4-oxo-1,4-dihydro-quinoline-3-carboxylate (**22b**)* S27

3.29 Preparation of *8-(4-(benzylcarbamoyl)-2-((fluorosulfonyl)oxy)phenyl)-4-oxo-1,4-dihydroquinoline-3-carboxylic acid (**3f**)* S27

3.30 Preparation of *8-(2-((fluorosulfonyl)oxy)-4-(phenethylcarbamoyl)phenyl)-4-oxo-1,4-dihydroquinoline-3-carboxylic acid (**3g**)* S28

IV. X-RAY CRYSTALLOGRAPHY S29

Table S1. X-ray diffraction data collection and refinement statistics S29

V. Dose-dependent curves of compound **3f** with TDP1 in different pre-incubation time S31

VI. ^{1}H and ^{13}C NMR spectra of compounds **2a – f** and **3a – g** S32

VII. Analytical HPLC of compounds **2a – f** and **3a – g** S45

VIII. HRMS of compounds **2a – f** and **3a – g** S52

SUPPLEMENTARY REFERENCES S59

I. SUPPLEMENTARY METHODS

1.1 Synthesis of Substituted Quinolones 2a – f. We prepared two distinct classes of quinolone platforms without (**2a – f**) or with 8-phenyl (**3a – h**) that were designed to probe a variety of protein interactions. A critical aspect of our current work involves the use of fluorosulfuryl imidazolium triflate salt (SuFEx-IT), 1-(fluorosulfonyl)-2,3-dimethyl-1*H*-imidazol-3-ium trifluoromethanesulfonate (FDIT) to introduce fluorosulfate or fluorosulfonyl functionality[1]. Heating commercially available ethyl 4-oxo-1,4-dihydroquinoline-3-carboxylate **4** with chlorosulfuric acid at 140 °C gave 8-(chlorosulfonyl)-4-oxo-1,4-dihydroquinoline-3-carboxylic acid **5**. Aqueous hydrolysis of sulfonyl chloride **5** gave the sulfonic acid **2a** (Scheme S1). Treatment of **5** with amines **6a – c** in THF yielded product substituted 4-oxo-1,4-dihydroquinoline-3-carboxylic acids **2b – d** following HPLC purification (Scheme 1). Reaction of **5** with potassium hydrogen fluoride (KHF_2) in acetonitrile gave the sulfonyl fluoride **2e**. Catalytic hydrogenation of the 8-nitro group in **1c**[2] afforded the 8-amino-containing quinolone **7**. Subsequent Michael-type addition[3] of the amino group in **7** with ethenesulfonyl fluoride (ESF) in DMF yielded the product quinolone **2f** having an ethyl-linked sulfonylfluoride group at the 8-position.

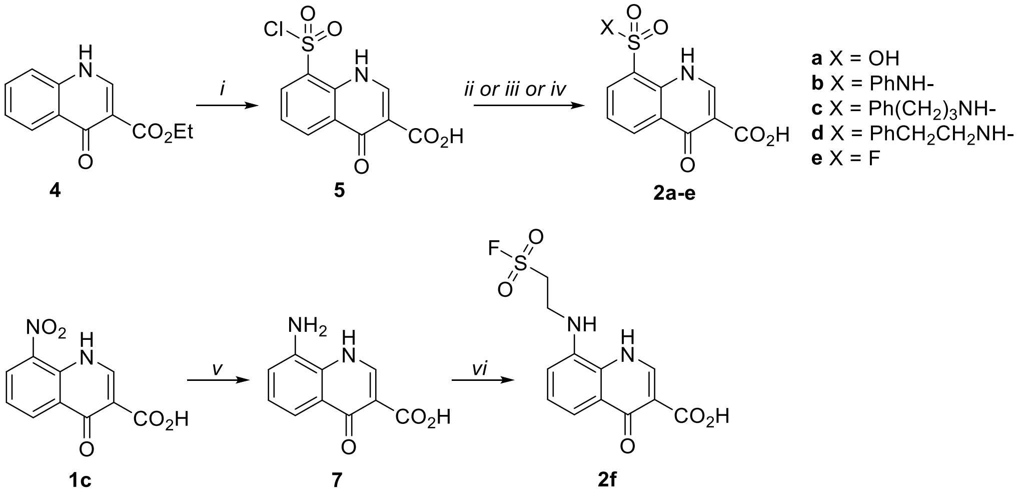

Scheme S1. Preparation of substituted quinolones **2a – f**. *Reagents and conditions:* (i) $ClSO_3H$, 140 °C; (ii) RNH_2 (**6a – c**); (iii) H_2O; (iv) KHF_2; (v) H_2, Pd/C, MeOH/DMF; (vi) $CH_2=CHSO_2F$ (ESF), DMF, 50 °C.

1.2 Synthesis of Substituted Quinolones 3a and 3b. The 8-phenylquinoline-3-carboxylic acids **3a** was prepared starting from commercially available [1,1'-biphenyl]-2-amine **8** using Gould-Jacobs thermal cyclization methodology (Scheme S2)[2,4-6]. Condensation of **8** with diethyl 2-(ethoxymethylene)malonate (DEEMM) at 120 °C provided the diethyl 2-((phenylamino)methylene)malonate **9**. Subsequent heating at 250 °C in Dowtherm® A (a thermally-stable organic heat transfer fluid composed of a eutectic mixture of 26.5% diphenyl with 73.5% diphenyl oxide) yielded the ethyl 4-oxo-1,4-dihydroquinoline-3-carboxylate **10** via a 6-electron cyclization process[5]. Saponification of **10** using sodium hydroxide in EtOH, followed by acidification (aqueous HCl) gave 8-phenyl substituted 4-oxo-1,4-dihydroquinoline-3-carboxylic acid **3a** (Scheme 2). Suzuki coupling of **1b**[2] with (2-aminophenyl)boronic acid **11** catalyzed by tetrakis(triphenylphosphine)palladium(0) [$Pd(PPh_3)_4$] in a solvent cocktail of toluene/EtOH/H_2O (10/7/3), gave 8-(2-aminophenyl)quinolone **12** (Scheme 2). Michael addition of the amino group in **12** with ESF in DMF gave quinolone **3b** having an ethylaminophenyl-linked sulfonylfluoride at the 8-position.

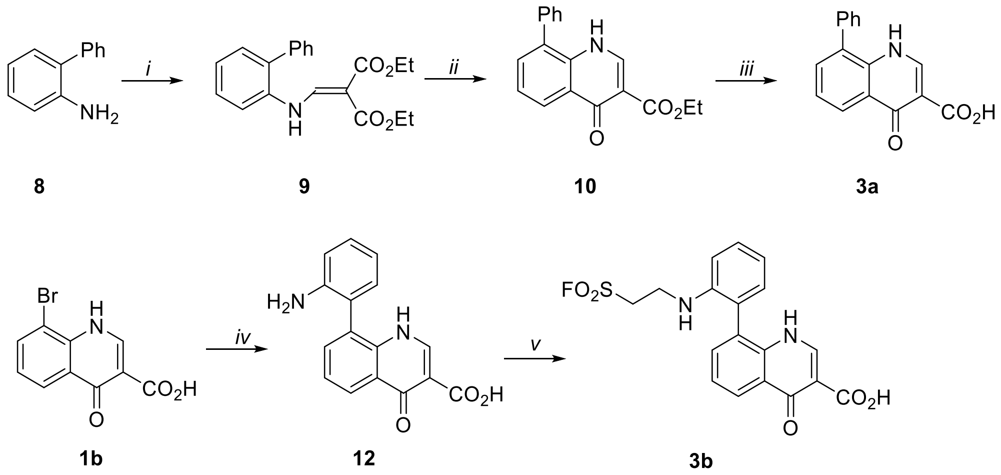

Scheme S2. Preparation of substituted quinolones **3a** and **3b**. *Reagents and conditions:* (i) $EtOCH=C(CO_2Et)_2$ (DEEMM), reflux; (ii) Ph_2O, 250 °C or Eaton's reagent, 100 °C; (iii) NaOH (aq., 2 N), 70°C; iv) *o*-NH_2-Ph-$B(OH)_2$ (**11**), $Pd(PPh_3)_4$, Na_2CO_3, Toluene/EtOH/H_2O, 80 °C; v) $CH_2=CHSO_2F$ (ESF), DMF, 50 °C.

1.3 Synthesis of Quinolones 3c – e. *Q*uinolone **1b** was protected as its *tert*-butyl ester using 1,1-di-*tert*-butoxy-N,N-dimethylmethylamine to give quinolone **13** (Scheme S3)[7]. Suzuki couplings with commercially available phenylboronic acids **14a – c**[8,9] gave the phenol-containing quinolones **15a – c**, which were converted as above to the product fluorosulfates **16a – c** (Scheme 3). Acidic deprotection of the tert-butyl ester groups gave the final product quinolones **3c – e** having 8-phenyl rings with fluorosulfate functionality at the *o*-, *m*-, *p*-positions.

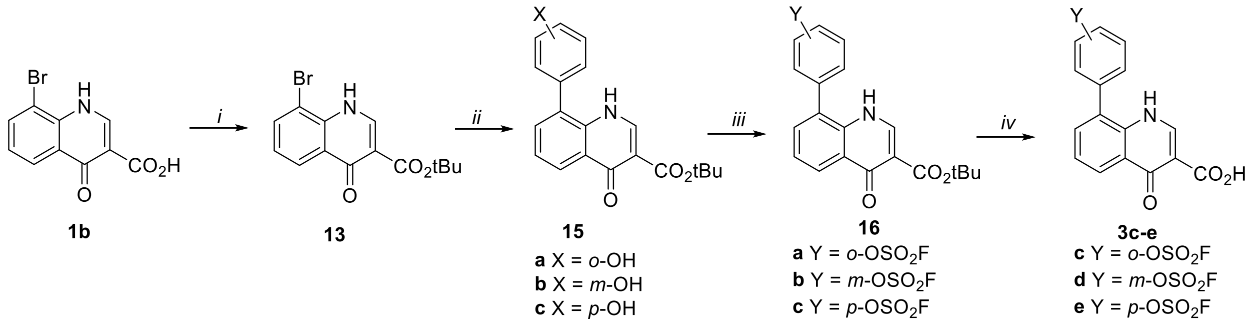

Scheme S3. Preparation of quinolones **3c – e**. *Reagents and conditions:* i) 1,1-di-tert-butoxy-*N,N*-dimethylmethylamine, 110 °C; ii) Ar-B(OH)$_2$ (**14a – c**), Pd(PPh$_3$)$_4$, Na$_2$CO$_3$, toluene / EtOH / H$_2$O, 80 °C; iii) FDIT, TEA, MeCN/THF, rt; iv) TFA/DCM.

1.4 Synthesis of Quinolones 3f and 3g. Substituted quinolones **3f** and **3g** were prepared with 8-phenyl rings having both *o*-fluorosulfate and *p*-amide starting from *tert*-butyl 8-bromo-4-oxo-1,4-dihydroquinoline-3-carboxylate **13** (Scheme S4). Benzoic acid **17** was coupled with amines **18a** or **18b** to provide the amides **19a** or **19b**. The boronates **20a** or **20b** were prepared using Miyaura-Borylation reactions of bromides **19a** or **19b** with bis(pinacolato)diboron B$_2$pin$_2$[10]. Suzuki coupling of the boronates **20a** or **20b** with t-*butyl* ester-protected bromide **13**, provided the phenols **21a** or **21b** (Scheme 4). Reaction of **21a** or **21b** with FDIT yielded the fluorosulfates **22a** or **22b**. Finally, TFA-mediated deprotection of tert-butyl ester led to the product quinolones **3f** and **3g** having *ortho*-fluorosulfates and *p*-amides on the 8-phenyl rings.

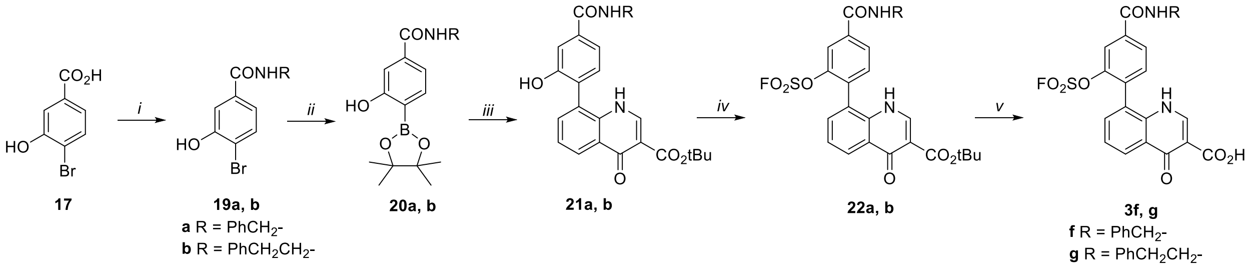

Scheme S4. Preparation of quinolones **3f** and **3g**. *Reagents and conditions:* i) RNH$_2$ (**18a, b**), HATU, TEA, DMF; ii) B$_2$pin$_2$, potassium propionate, Pd(PPh$_3$)$_2$Cl$_2$, toluene, 80 °C; iii) **13**, Pd(PPh$_3$)$_4$, K$_2$CO$_3$, toluene/EtOH/H$_2$O, 80 °C; iv) FDIT, TEA, MeCN/THF, rt; v) TFA/DCM.

II. EXPERIMENTAL SECTION

General Synthetic Procedures. Proton (^{1}H) and carbon (^{13}C) NMR spectra were recorded on a Varian 400 MHz spectrometer or a Varian 500 MHz spectrometer and are reported in ppm relative to TMS and referenced to the solvent in which the spectra were collected. Solvent was removed by rotary evaporation under reduced pressure, and anhydrous solvents were obtained commercially and used without further drying. Purification by silica gel chromatography was performed using Combiflash with EtOAc−hexanes or DCM/MeOH solvent systems. Preparative high pressure liquid chromatography (HPLC) was conducted either using a Waters 2545 Binary Grandient Module system having Waters 2998 photodiode array detector, Waters 2707 autosampler, Waters fraction collector III and Phenomenex C18 columns (catalogue no. 00G-4436-P0-AX, 250 mm × 21.2 mm 10 µm particle size, 110 Å pore) at a flow rate of 20 mL/min. or a Teledyne ISCO CombiFlash® EZ Prep system having Redisep Gold® C18 reversed phase column (catalogue no. 69-2203-336, 50 g, 20-40 µm spherical particle size, 100 Å pore) at a flow rate of 40 mL/min. Binary solvent systems consisting of A = 0.1% aqueous TFA and B = 0.1% TFA in acetonitrile were employed with gradients as indicated. Products were obtained as amorphous solids following lyophilization. Analytical HPLC spectra were recorded using an Agilent 1260 Infinity HPLC system and Phenomenex C18 column (catalogue no. 00G-4435-E0, 250 × 4.6 mm, Gemini 5µm particle size, 110 Å pore) at a flow rate of 1.0 mL/min. Binary solvent systems consisting of A = 0.1% aqueous TFA and B = 0.1% TFA in acetonitrile for analytical HPLC were employed with the following gradient: B 0% for 2 min., 0 to 10% for 3 min., and 10% to 95% for 22 min. Electrospray ionization-mass spectrometric (ESI-MS) were acquired with an Agilent LC/MSD system equipped with a multimode ion source or Shimadzu LC-MS 2020 ESI and APCI dual ionization-mass spectrometric (DUIS-MS) system. High

resolution mass spectrometry (HRMS) were acquired using an Agilent 6520 Accurate-Mass Q-TOF LC/MS system.

2.1 General Procedure A. Preparation of the substituted 4-oxo-1,4-dihydroquinoline-3-carboxylic acids (2a – e).

The solid of 8-(chlorosulfonyl)-4-oxo-1,4-dihydroquinoline-3-carboxylic acid (**5**, 0.4 mmol) and water or substituted amine (**6a – c**, 8.0 mmol) were dissolved in THF (3.0 mL). The reaction mixture was stirred (rt, 4 h). The mixture was dissolved in DMF and purified by HPLC. The substituted 4-oxo-1,4-dihydroquinoline-3-carboxylic acid (**2b – d**) were afforded.

2.2 General Procedure B. Preparation of fluorosulfates (16a – c, 22a, b) using SuFEx click chemistry[1].

To a solution of phenols (**15a – c** or **21a, b**, 0.3 mmol) and triethylamine (2 mmol) in MeCN/THF (1:1, 4.0 mL), commercially available fluorosulfuryl imidazolium triflate salt (SuFEx-IT) 1-(fluorosulfonyl)-2,3-dimethyl-1H-imidazol-3-ium trifluoromethanesulfonate (FDIT, 1 mmol) was added. The reaction was stirred (rt, 2 h) and monitored using TLC and LC/MS analysis. After completion, the reaction was purified by silica gel chromatography to afford the fluorosulfates (**16a – c, 22a, b**).

2.3 General Procedure C. Preparation of phenols (15a – c) using Suzuki coupling.

A mixture of tert-butyl 8-bromo-4-oxo-1,4-dihydroquinoline-3-carboxylate (**13**, 0.6 mmol), commercially available boronated phenol (**14a – c**, 3.0 mmol), and Na_2CO_3 (5.0 mmol) was

suspended in toluene/EtOH/H_2O (5:1:1, 14 mL) under argon. The suspension was degassed using argon. $Pd(PPh_3)_4$ (30 µmol) was added. The reaction mixture was refluxed (16 h). After cooling to rt, the reaction suspension was concentrated with Celite and solid-loaded into a silica gel column. After purification, phenols (**15a – c**) were afforded.

2.4 General Procedure D. Preparation of acids (3c – g).

The *tert*-butyl ester (**16a – c** or **22a, b,** 0.331 mmol) was mixed in DCM/TFA (1:1, 2 mL). The reaction was stirred (rt, 30 min.). The reaction mixture was concentrated. After HPLC purification (Teledyne ISCO CombiFlash® EZ Preparative HPLC), acids (**3c – g**) were afforded.

2.5 General Procedure E. Preparation of amides (19a, b).

To the solution of 4-bromo-3-hydroxybenzoic acid (**17**, 10.0 g, 46 mmol) in DMF (55 mL), HATU (17.5 g, 46 mmol) and TEA (12.9 mL, 92 mmol) were added. The reaction suspension was incubated (15 min.). Amine (**18a, b**, 115 mmol) was added. The reaction was stirred (rt, 3 h). Reaction mixture was concentrated, and the residue was purified in a silica gel column chromatography. Amides (**19a, b**) were afforded.

2.6 General Procedure F. Preparation of boronate esters (20a, b) and quinolones (21a, b) using Miyaura-Borylation reaction[11,12].

The reaction mixture of bromide (**19a, b**, 6.5 mmol), 4,4,4',4',5,5,5',5'-octamethyl-2,2'-bi(1,3,2-dioxaborolane) ($Bpin_2$, 7.9 mmol), potassium propionate (17 mmol) and $Pd(PPh_3)_2Cl_2$ (0.2

mmol) in toluene (15 mL) was stirred under argon and refluxed (85°C, 8 h). The reaction mixture was cooled to rt and diluted by EtOAc. The organic phase was washed by NH_4Cl (sat. aq.), brine and dried by anhydrous $MgSO_4$. The crude product was filtered and concentrated. The boronate esters (**20a, b**) was afforded and used next step directly without further purification to avoid the decomposition. A mixture of tert-butyl 8-bromo-4-oxo-1,4-dihydroquinoline-3-carboxylate (**25**, 0.6 mmol), the boronate esters (**20a, b**, 3.0 mmol), and K_2CO_3 (5.0 mmol) was suspended in toluene/EtOH/H_2O (5:1:1, 14 mL) under argon. The suspension was degassed using argon. $Pd(PPh_3)_4$ (30 µmol) was added. The reaction mixture was refluxed (16 h). After cooling to rt, the reaction suspension was concentrated with Celite and solid-loaded into a silica gel column. After purification, quinolones (**21a, b**) were afforded.

III. COMPOUND PREPARATION

3.1 Preparation of *8-(chlorosulfonyl)-4-oxo-1,4-dihydroquinoline-3-carboxylic acid (5)*. Commercially available ethyl 4-oxo-1,4-dihydroquinoline-3-carboxylate (**4**, 1.0 g, 4.8 mmol) was added to chlorosulfuric acid (2.0 mL). The clear brown mixture was stirred (140 °C, 3.5 h). The reaction mixture was quenched by pouring into ice and extracted by chloroform and dried by Na_2SO_4. The solvent was filtered and concentrated. The residue was purified by silica gel chromatography. The fraction was collected and concentrated. The pale grey solid 8-(chlorosulfonyl)-4-oxo-1,4-dihydroquinoline-3-carboxylic acid (**5**, 197 mg) was afforded (14 % yield). ^{1}H NMR (400 MHz, $CDCl_3$) δ 13.94 (brs, 1H), 10.77 (brs, 1H), 9.00 – 8.89 (m, 2H), 8.53 (d, J = 6.2 Hz, 1H), 7.77 (t, J = 8.0 Hz, 1H). ESI-MS m/z: 288.0 (MH^+).

3.2 Preparation of *4-oxo-8-sulfo-1,4-dihydroquinoline-3-carboxylic acid (2a)*.

Treatment of 8-(chlorosulfonyl)-4-oxo-1,4-dihydroquinoline-3-carboxylic acid (**5**) and water as outlined in general procedure A and purification by preparative HPLC (CAT# 00G-4436-P0-AX) (linear gradient of 10% B to 50% B over 30 min with a flow rate 10 mL/min, retention time = 16.7 min.) provided the title compound (**2a**) as a white solid (34% yield). ^{1}H NMR (500 MHz, DMSO-d6) δ 12.57 (brs, 1H), 9.01 (d, *J* = 7.2 Hz, 1H), 8.35 (dd, *J* = 8.1, 1.6 Hz, 1H), 8.18 (dd, *J* = 7.4, 1.6 Hz, 1H), 7.64 – 7.57 (m, 1H). ^{13}C NMR (126 MHz, DMSO-d6) δ 178.70, 166.62, 146.33, 137.16, 135.46, 132.15, 126.92, 125.87, 125.42, 108.00. ESI-MS m/z: 269.9 (MH^+), 539.0 (M_2H^+). HRMS (ESI) m/z calcd. for $C_{10}H_8NO_6S^+$ (MH^+), 270.0067; found, 270.0067 (Δ = -0.06 ppm); m/z calcd. for $C_{20}H_{15}N_2O_{12}S_2^+$ (M_2H^+), 539.0061; found, 539.0069 (Δ = -1.5 ppm).

3.3 Preparation of *4-oxo-8-(N-phenylsulfamoyl)-1,4-dihydroquinoline-3-carboxylic acid (2b)*.

Treatment of 8-(chlorosulfonyl)-4-oxo-1,4-dihydroquinoline-3-carboxylic acid (**5**) and aniline (**6a**) as outlined in general procedure A and purification by preparative HPLC (CAT# 00G-4436-P0-AX) (linear gradient of 20% B to 100% B over 30 min with a flow rate 10 mL/min, retention time = 17.4 min.) provided the title compound (**2b**) as a white fluffy solid (49% yield). ^{1}H NMR (400 MHz, DMSO-d_6) δ 14.59 (brs, 1H), 12.01 (brs, 1H), 10.77 (brs, 1H), 8.90 (s, 1H), 8.57 (dd, *J* = 8.1, 1.5 Hz, 1H), 8.23 (dd, *J* = 7.6, 1.5 Hz, 1H), 7.70 (t, *J* = 7.9 Hz, 1H), 7.29 – 7.20 (m, 2H), 7.13 – 7.02 (m, 3H). ^{13}C NMR (101 MHz, DMSO-d_6) δ 178.13, 165.90, 147.20, 136.67, 134.97, 131.75, 129.89 (3C), 126.31, 125.94 (2C), 122.13 (3C), 109.09. ESI-MS m/z: 345.0 (MH^+), 367.0 (MNa^+). HRMS (ESI) m/z calcd. for $C_{16}H_{13}N_2O_5S^+$ (MH^+), 345.0540; found, 345.0540 (Δ = -0.09 ppm).

3.4 Preparation of *4-oxo-8-(N-phenethylsulfamoyl)-1,4-dihydroquinoline-3-carboxylic acid (2c)*.

Treatment of 8-(chlorosulfonyl)-4-oxo-1,4-dihydroquinoline-3-carboxylic acid (**5**) and 2-phenylethan-1-amine (**6b**) as outlined in general procedure A and purification by preparative HPLC (CAT# 00G-4436-P0-AX) (linear gradient of 20% B to 100% B over 30 min with a flow rate 10 mL/min, retention time = 18.1 min.) provided the title compound (**2c**) as a white solid (78% yield). ^{1}H NMR (500 MHz, DMSO-d$_6$) δ 14.74 (brs, 1H), 12.02 (brs, 1H), 8.87 (s, 1H), 8.57 (dd, *J* = 8.2, 1.5 Hz, 1H), 8.30 (dd, *J* = 7.5, 1.5 Hz, 1H), 8.27 (d, *J* = 6.0 Hz, 1H), 7.73 (t, *J* = 7.8 Hz, 1H), 7.16 - 7.13 (m, 2H), 7.11 – 7.06 (m, 3H), 3.17 (q, *J* = 6.7 Hz, 2H), 2.68 (t, *J* = 7.1 Hz, 2H). ^{13}C NMR (101 MHz, DMSO-d$_6$) δ 178.38, 165.99, 146.95, 138.67, 135.37, 134.75, 131.12, 129.29, 129.04 (2C), 128.55 (2C), 126.64, 126.41, 125.99, 109.02, 44.31, 35.46. ESI-MS m/z: 373.0 (MH$^+$), 395.0 (MNa$^+$). HRMS (ESI) m/z calcd. for $C_{18}H_{17}N_2O_5S^+$ (MH$^+$), 373.0853 (Δ = -0.62 ppm); found, 373.0855; m/z calcd. for $C_{36}H_{32}N_4O_{10}S_2Na^+$ (M_2Na^+), 767.1452; found, 767.1444 (Δ = 1.05 ppm).

3.5 Preparation of *4-oxo-8-(N-(3-phenylpropyl)sulfamoyl)-1,4-dihydroquinoline-3-carboxylic acid (2d)*.

Treatment of 8-(chlorosulfonyl)-4-oxo-1,4-dihydroquinoline-3-carboxylic acid (**5**) and 3-phenylpropan-1-amine (**6c**) as outlined in general procedure A and purification by preparative HPLC (CAT# 00G-4436-P0-AX) (linear gradient of 20% B to 100% B over 30 min with a flow rate 10 mL/min, retention time = 19.5 min.) provided the title compound (**5c**) as a white solid (71% yield). ^{1}H NMR (400 MHz, DMSO-d$_6$) δ 14.71 (brs, 1H), 12.12 (brs, 1H), 8.94 (s, 1H), 8.61 (dd, *J* = 8.1, 1.4 Hz, 1H), 8.33 (dd, *J* = 7.6, 1.4 Hz, 1H), 8.28 (d, *J* = 5.9 Hz, 1H), 7.78 (t, *J*

= 7.9 Hz, 1H), 7.21 (t, *J* = 7.3 Hz, 2H), 7.13 (t, *J* = 7.3 Hz, 1H), 7.06 (d, *J* = 7.0 Hz, 2H), 2.89 (q, *J* = 6.6 Hz, 2H), 2.52 – 2.49 (m, 2H), 1.66 (p, *J* = 7.1 Hz, 2H). ^{13}C NMR (101 MHz, DMSO-d_6) δ 176.23, 163.87, 145.02, 139.39, 133.46, 132.59, 129.05, 127.24, 126.60 (2C), 126.50 (2C), 124.29, 124.15, 123.98, 106.91, 40.38, 30.26, 28.93. ESI-MS m/z: 387.1 (MH^+), 409.0 (MNa^+). HRMS (ESI) m/z calcd. for $C_{19}H_{19}N_2O_5S^+$ (MH^+), 387.1009; found, 387.1010 (Δ = -0.21 ppm); m/z calcd. for $C_{38}H_{36}N_4O_{10}S_2Na^+$ (M_2Na^+), 765.1765; found, 795.1757 (Δ = 1.01 ppm).

3.6 Preparation of *8-(fluorosulfonyl)-4-oxo-1,4-dihydroquinoline-3-carboxylic acid (2e)*[13].

Compound 8-(chlorosulfonyl)-4-oxo-1,4-dihydroquinoline-3-carboxylic acid (**5**, 0.15 g, 0.51 mmol) was dissolved in MeCN (588 µL) and subsequently added to a sat. solution of potassium hydrogen fluoride (0.092 g, 1.18 mmol) in H_2O (259 µL). The reaction mixture was stirred (rt, 3 h). The solution was extracted with EtOAc, and washed with brine, dried over Na_2SO_4. The solution was filtered and concentrated. After purification by preparative HPLC, the title compound (**2e**, 0.12 g, 0.43 mmol) was afforded as a white fluffy solid (85% yield). ^{1}H NMR (400 MHz, $CDCl_3$) δ 13.91 (brs, 1H), 10.67 (brs, 1H), 8.96 (dd, *J* = 8.1, 1.6 Hz, 1H), 8.92 (d, *J* = 5.3 Hz, 1H), 8.53 (dd, *J* = 7.7, 1.6 Hz, 1H), 7.79 (t, *J* = 7.9 Hz, 1H). ^{1}H NMR (500 MHz, DMSO-d_6) δ 8.88 – 8.77 (m, 2H), 8.64 (d, *J* = 6.0 Hz, 1H), 7.82 (t, *J* = 7.9 Hz, 1H). ^{13}C NMR (126 MHz, DMSO-d_6) δ 176.97, 166.53, 163.52, 149.14, 136.83, 135.15, 126.97 (2C), 125.40, 109.88. ^{19}F NMR (376 MHz, DMSO-d_6) δ 65.90. ESI-MS m/z: 272.0 (MH^+). HRMS (ESI) m/z calcd. for $C_{10}H_7FNO_5S^+$ (MH^+), 272.0023 (Δ = -0.56 ppm); found, 272.0025; m/z calcd. for $C_{20}H_{12}F_2N_2O_{10}S_2Na^+$ (M_2Na^+), 564.9794; found, 564.9791 (Δ = 0.47 ppm).

3.7 Preparation of *8-((2-(fluorosulfonyl)ethyl)amino)-4-oxo-1,4-dihydroquinoline-3-carboxylic acid (2f)*.

The solution of 8-nitro-4-oxo-1,4-dihydroquinoline-3-carboxylic acid[2] (**1c**, 0.69 g, 2.9 mmol) in MeOH/DMF (3:2) was mixed with Pd/C (10%) and purged by hydrogen. The reaction mixture was stirred hydrogen under hydrogen (rt, 2 h). The suspension was filtered by a pad of silica gel and concentrated to afford crude 8-Amino-4-oxo-1,4-dihydroquinoline-3-carboxylic acid [**7**, 333A-176, ESI-MS m/z: 205.1 (MH^+)] which was used in next step directly. Commercially available ethenesulfonyl fluoride (0.22 mL, 2.7 mmol) was added to the solution of *8-amino-4-oxo-1,4-dihydroquinoline-3-carboxylic acid (7)* in DMF (10 mL). The reaction was stirred (50 °C, 16 h). The reaction mixture was concentrated. After purification by preparative HPLC, the title compound (**2f**) was afforded as a beige solid. ^{1}H NMR (500 MHz, DMSO-d6) δ 15.47 (s, 1H), 12.62 (s, 1H), 8.73 (s, 1H), 7.58 (d, J = 8.1 Hz, 1H), 7.38 (t, J = 7.9 Hz, 1H), 7.08 (d, J = 7.8 Hz, 1H), 4.20 (q, J = 6.1 Hz, 2H), 3.78 (q, J = 6.0 Hz, 2H). ^{13}C NMR (126 MHz, DMSO-d_6) δ 178.77, 166.99, 163.52, 144.31, 138.05, 127.28, 125.90, 113.76, 113.36, 107.81, 49.64 (d, J = 11.6 Hz), 38.20. ^{19}F NMR (376 MHz, DMSO-d6) δ 58.05. ESI-MS m/z: 315.1 (MH^+). HRMS (ESI) m/z calcd. for $C_{12}H_{12}FN_2O_5S^+$ (MH^+), 315.0445; found, 315.0448 (Δ = -0.8 ppm).

3.8 Preparation of *diethyl 2-(([1,1'-biphenyl]-2-ylamino)methylene)malonate (9)*.

Using Gould-Jacob cyclization[2,4-6], a mixture of commercially available [1,1'-biphenyl]-2-amine (**8**, 763 mg, 4.5 mmol) and diethyl 2-(ethoxymethylene)malonate (DEEMM, 4.5 mmol) was heated (120 °C, 4 h). The reaction was cooled to rt. The formed white solid was filtered and washed with hexanes. The title compound (**9**, 1.36 g) was afforded as a white solid (89% yield). ^{1}H NMR (400 MHz, $CDCl_3$) δ 10.73 (d, J = 13.6 Hz, 1H), 8.51 (d, J = 13.7 Hz, 1H), 7.54 – 7.49

(m, 2H), 7.47 – 7.40 (m, 4H), 7.36 – 7.33 (m, 2H), 7.25 (td, *J* = 7.4, 1.2 Hz, 1H), 4.25 (q, *J* = 7.1 Hz, 2H), 4.15 (q, *J* = 7.1 Hz, 2H), 1.33 (t, *J* = 7.1 Hz, 3H), 1.22 (t, *J* = 7.1 Hz, 3H). ^{13}C NMR (101 MHz, $CDCl_3$) δ 167.63, 165.94, 151.80, 137.35, 136.97, 132.85, 131.21, 129.27 (2C), 129.02 (2C), 128.90, 128.13, 124.96, 116.51, 94.18, 60.07, 60.05, 14.45, 14.20. ESI-MS m/z: 340.1 (MH^+), 701.3 (M_2Na^+).

3.9 Preparation of *ethyl 4-oxo-8-phenyl-1,4-dihydroquinoline-3-carboxylate (10)*[5].

Diethyl 2-(([1,1'-biphenyl]-2-ylamino)methylene)malonate (**9**, 433 mg, 1.3 mmol) was suspended in Dowtherm A (5.0 mL) (eutectic mixture of 26.5% diphenyl + 73.5% diphenyl oxide). The reaction mixture was heated (250 °C, 2 h). The resultant mixture was cooled to rt and filtered. The solid was washed by Et_2O (10 mL X 3). The title compound (**10,** 157 mg) was afforded as a white solid (42% yield). ^{1}H NMR (400 MHz, $CDCl_3$) δ 8.73 (s, 1H), 8.43 (d, *J* = 8.2 Hz, 1H), 7.66 – 7.64 (m, 1H), 7.54 – 7.49 (m, 5H), 7.47 – 7.43 (m, 1H), 7.28 (s, 1H), 4.40 (q, *J* = 6.8 Hz, 2H), 1.41 (t, *J* = 7.1 Hz, 3H). ESI-MS m/z: 294.1 (MH^+), 316.0 (MNa^+), 609.1 (M_2Na^+).

3.10 Preparation of *4-oxo-8-phenyl-1,4-dihydroquinoline-3-carboxylic acid (3a)*.

Ethyl 4-oxo-8-phenyl-1,4-dihydroquinoline-3-carboxylate (**10**, 146 mg, 0.5 mmol) was suspended in NaOH (2N, 4 mL) and EtOH (0.6 mL). The mixture was stirred (70 °C, 4 h). The reaction mixture was cooled to rt and acidified by HCl (aq., 2N). The formed white suspension was filtered and washed by water. After purification by preparative HPLC, the title compound (**3a**, 124 mg) was afforded as a white solid (94% yield). ^{1}H NMR (400 MHz, DMSO-d_6) δ 15.23 (brs, 1H), 12.04 (s, 1H), 8.58 (d, *J* = 5.7 Hz, 1H), 8.37 (dd, *J* = 8.2, 1.6 Hz, 1H), 7.80 (dd, *J* =

7.3, 1.6 Hz, 1H), 7.69 (dd, J = 8.1, 7.3 Hz, 1H), 7.66 – 7.55 (m, 5H). ^{13}C NMR (101 MHz, DMSO-d_6) δ 178.98, 166.59, 145.85, 136.95, 135.98, 135.50, 133.28, 130.12 (2C), 129.79 (2C), 129.24, 126.52, 125.48, 125.13, 107.96. ESI-MS m/z: 266.1 (MH^+), 288.0 (MNa^+), 553.1 (M_2Na^+). HRMS (ESI) m/z calcd. for $C_{16}H_{12}NO_3$ (MH^+), 266.0812; found, 266.0813 (Δ = -0.49 ppm); m/z calcd. for $C_{32}H_{22}N_2O_6Na^+$ (M_2Na^+), 553.1370; found, 553.1361 (Δ = -1.64 ppm).

3.11 Preparation of *8-(2-aminophenyl)-4-oxo-1,4-dihydroquinoline-3-carboxylic acid (12)*.

The mixture of 8-bromo-4-hydroxyquinoline-3-carboxylic acid[2] (**1b**, 173 mg, 0.65 mmol), (2-aminophenyl)boronic acid (**11**, 442 mg, 3.22 mmol), tetrakis(triphenylphosphine)palladium(0) (37.0 mg, 0.032 mmol) and Na_2CO_3 (476 mg, 4.49 mmol) were suspended in toluene/EtOH/H_2O (10/7/3, 20 mL). The reaction mixture was stirred and refluxed overnight under argon. After cooling to rt, the reaction mixture was concentrated, quenched to pH 3 with HCl (aq., 2.0 M) and extracted with DCM. The organic layer was dried ($MgSO_4$), filtered, concentrated, and the residue was purified in reverse phased C18 column using automated flash chromatography (H_2O/CAN 0.1 % TFA) to afford the title compound (**12**) as a beige solid (95% yield). ^{1}H NMR (400 MHz, DMSO-d_6) δ 15.34 (s, 1H), 8.57 (s, 1H), 8.36 (d, J = 7.8 Hz, 1H), 7.69 (dt, J = 15.2, 7.3 Hz, 2H), 7.25 (t, J = 7.7 Hz, 1H), 7.06 (d, J = 7.5 Hz, 1H), 6.90 (d, J = 8.2 Hz, 1H), 6.77 (t, J = 7.4 Hz, 1H). ^{13}C NMR (126 MHz, DMSO-d_6) δ 179.29, 166.78, 45.60, 137.67, 136.25, 131.62, 131.11, 130.36, 126.76, 125.60, 125.05, 119.77, 117.54, 116.41, 115.18, 107.96. ESI-MS m/z: 281.1 (MH^+).

3.12 Preparation of *8-(2-((2-(fluorosulfonyl)ethyl)amino)phenyl)-4-oxo-1,4-dihydroquinoline-3-carboxylic acid (3b)*.

The suspension of 8-(2-aminophenyl)-4-oxo-1,4-dihydroquinoline-3-carboxylic acid (**12**, 126 mg, 0.448 mmol) and ethenesulfonyl fluoride (0.074 ml, 0.897 mmol) in DMF (3 mL) was stirred and microwave-heated (50 °C, 16 h) in a Biotage vessel. The reaction mixture was concentrated and the crude residue was purified by HPLC. The title compound (**3b**) was afforded as a beige fluffy solid. ^{1}H NMR (400 MHz, DMSO-d_6) δ 15.34 (s, 1H), 11.69 (s, 1H), 8.51 (d, *J* = 6.8 Hz, 1H), 8.38 (dd, *J* = 6.6, 3.2 Hz, 1H), 7.73 – 7.66 (m, 2H), 7.42 (ddd, *J* = 8.6, 7.4, 1.7 Hz, 1H), 7.12 (dd, *J* = 7.4, 1.6 Hz, 1H), 6.91 – 6.82 (m, 2H), 4.95 (s, 1H), 4.12 – 3.94 (m, 2H). ^{13}C NMR (101 MHz, DMSO-d_6) δ 179.29, 166.75, 145.82, 145.19, 137.77, 136.55, 131.97, 130.88, 130.46, 126.85, 125.58, 125.35, 121.13, 117.87, 111.37, 107.98, 49.69 (d, *J* = 11.0 Hz), 37.49. ^{19}F NMR (376 MHz, DMSO-d_6) δ 56.89. ESI-MS m/z: 391.1 (MH^+). HRMS (ESI) m/z calcd. for $C_{18}H_{16}FN_2O_5S^+$ (MH^+), 391.0758; found, 391.0765 (Δ = -1.67 ppm).

3.13 Preparation of *tert-butyl 8-bromo-4-oxo-1,4-dihydroquinoline-3-carboxylate (13)*.

Commercially available 1,1-di-tert-butoxy-N,N-dimethylmethanamine (15 mL, 62.6 mmol) was added dropwise to 8-bromo-4-oxo-1,4-dihydroquinoline-3-carboxylic acid (**1b,** 2.1 g, 7.85 mmol). The reaction mixture was stirred and refluxed (24 h). The reaction was cooled to rt and diluted with water. The mixture was extracted with EtOAc and dried ($MgSO_4$). The crude sample was concentrated and purified via silica gel chromatography. The title compound (**13**, 1.98 g) was afforded as a pale white solid (78 % yield). ^{1}H NMR (400 MHz, $CDCl_3$) δ 9.24 (s, 1H), 8.26 (d, *J* = 6.9 Hz, 1H), 8.07 (d, *J* = 6.1 Hz, 1H), 7.39 (t, 1H), 1.63 (s, 9H), 1.42 (s, 9H). ^{13}C NMR (101 MHz, $CDCl_3$) δ 164.68, 160.52, 152.16, 147.66, 134.55, 128.65, 126.43, 124.94, 124.37, 122.10, 87.21, 82.40, 29.18, 28.16. ESI-MS m/z: 326.2 (MH^+).

3.14 Preparation of *tert-butyl 8-(2-hydroxyphenyl)-4-oxo-1,4-dihydroquinoline-3-carboxylate (15a)*.

Treatment of tert-butyl 8-bromo-4-oxo-1,4-dihydroquinoline-3-carboxylate ***(13)** and* 4-(4,4,5,5-tetramethyl-1,3,2-dioxaborolan-2-yl)phenol (**14a**) as outlined in general procedure C provided the title compound (**15a**) as a white solid (97% yield). ^{1}H NMR (400 MHz, CD_3OD) δ 8.47 (s, 1H), 8.35 (dd, *J* = 8.1, 1.6 Hz, 1H), 7.58 (dd, *J* = 7.3, 1.6 Hz, 1H), 7.49 (dd, *J* = 8.1, 7.2 Hz, 1H), 7.36 – 7.30 (m, 1H), 7.24 (dd, *J* = 7.8, 1.7 Hz, 1H), 7.02 – 6.96 (m, 2H), 1.57 (s, 9H). ^{13}C NMR (126 MHz, CD_3OD) δ 177.94, 165.67, 156.20, 146.16, 138.37, 135.73, 132.78, 131.47, 131.44, 128.73, 126.33, 125.99, 124.11, 121.31, 117.18, 111.87, 81.94, 28.58 (3C). ESI-MS m/z: 338.2 (MH^+).

3.15 Preparation of *tert-butyl 8-(3-((fluorosulfonyl)oxy)phenyl)-4-oxo-1,4-dihydroquinoline-3-carboxylate (15b)*.

Treatment of tert-butyl 8-bromo-4-oxo-1,4-dihydroquinoline-3-carboxylate ***(13)** and* 3-(4,4,5,5-tetramethyl-1,3,2-dioxaborolan-2-yl)phenol (**14b**) as outlined in general procedure C provided the title compound (**15b**) as a white solid (99% yield). ^{1}H NMR (400 MHz, CD_3OD) δ 8.47 (s, 1H), 8.36 (dd, *J* = 8.1, 1.6 Hz, 1H), 7.68 – 7.59 (m, 2H), 7.51 (t, *J* = 7.7 Hz, 1H), 7.39 (t, *J* = 7.9 Hz, 1H), 6.96 – 6.90 (m, 2H), 6.88 (t, *J* = 2.0 Hz, 1H), 1.58 (s, 9H). ^{13}C NMR (126 MHz, CD_3OD) δ 177.68, 165.55, 159.44, 146.36, 138.73, 137.57, 134.81, 131.60, 130.03, 129.94, 126.54, 126.05, 121.53, 117.34, 116.80, 112.11, 82.06, 28.56 (3C). ESI-MS m/z: 338.1 (MH^+).

3.16 Preparation of *tert-butyl 8-(4-hydroxyphenyl)-4-oxo-1,4-dihydroquinoline-3-carboxylate (16c)*.

Treatment of tert-butyl 8-bromo-4-oxo-1,4-dihydroquinoline-3-carboxylate ***(13)*** *and* 3-(4,4,5,5-tetramethyl-1,3,2-dioxaborolan-2-yl)phenol (**14c**) as outlined in general procedure C provided the title compound (**15c**) as a white solid (63% yield). ^{1}H NMR (400 MHz, CD_3OD) δ 8.47 (s, 1H), 8.33 (dd, *J* = 8.1, 1.6 Hz, 1H), 7.60 (dd, *J* = 7.2, 1.6 Hz, 1H), 7.49 (t, *J* = 8.2, 7.2 Hz, 1H), 7.35 – 7.26 (m, 2H), 7.03 – 6.93 (m, 2H), 1.58 (s, 9H). ^{13}C NMR (126 MHz, CD_3OD) δ 177.79, 165.59, 159.34, 146.30, 137.85, 135.06, 134.17, 131.83 (2C), 130.00, 129.09, 128.29, 126.08, 117.23 (2C), 112.02, 82.03, 28.57 (3C). ESI-MS (m/z): 338.1 (MH^+).

3.17 Preparation of *tert-butyl 8-(2-((fluorosulfonyl)oxy)phenyl)-4-oxo-1,4-dihydroquinoline-3-carboxylate (16a)*.

Treatment of tert-butyl 8-(2-hydroxyphenyl)-4-oxo-1,4-dihydroquinoline-3-carboxylate (**15a**) as outlined in general procedure B provided the title compound (**16a**) as a white solid (55% yield). ^{1}H NMR (400 MHz, CD_3OD) δ 8.47 (dd, *J* = 8.1, 1.5 Hz, 1H), 8.43 (s, 1H), 7.77 – 7.61 (m, 5H), 7.57 (t, *J* = 7.7 Hz, 1H), 1.59 (s, 9H). ^{13}C NMR (126 MHz, CD_3OD) δ 177.37, 165.39, 149.55, 146.39, 138.24, 136.08, 134.29, 132.65, 131.00, 130.82, 129.13, 128.13, 126.92, 125.90, 123.36, 112.52, 82.20, 28.54 (3C). ^{19}F NMR (376 MHz, CD_3OD) δ 37.97. ESI-MS m/z: 420.1 (MH^+).

3.18 Preparation of *tert-butyl 8-(3-((fluorosulfonyl)oxy)phenyl)-4-oxo-1,4-dihydroquinoline-3-carboxylate (16b)*.

Treatment of tert-butyl 8-(3-((fluorosulfonyl)oxy)phenyl)-4-oxo-1,4-dihydroquinoline-3-carboxylate (**15b***)* as outlined in general procedure B provided the title compound (**16b**) as a white solid (53% yield). ^{1}H NMR (400 MHz, CD_3OD) δ 8.45 (s, 1H), 8.42 (dd, *J* = 8.2, 1.5 Hz, 1H), 7.76 (dd, *J* = 9.1, 7.5 Hz, 1H), 7.69 – 7.61 (m, 4H), 7.54 (t, *J* = 7.7 Hz, 1H), 1.58 (s, 9H). ^{13}C NMR (101 MHz, CD_3OD) δ 177.42, 165.41, 152.02, 146.49, 140.52, 137.63, 135.25, 132.66, 131.72, 131.42, 129.24, 127.59, 126.10, 123.53, 122.33, 112.45, 82.15, 28.55 (3C). ^{19}F NMR (376 MHz, CD_3OD) δ 36.10. ESI-MS m/z: 420.1 (MH^+).

3.19 Preparation of *tert-butyl 8-(4-((fluorosulfonyl)oxy)phenyl)-4-oxo-1,4-dihydroquinoline-3-carboxylate (16c).*

Treatment of tert-butyl 8-(4-hydroxyphenyl)-4-oxo-1,4-dihydroquinoline-3-carboxylate (**15c**) as outlined in general procedure B provided the title compound (**16c**) as a white solid (56% yield). ^{1}H NMR (500 MHz, $CDCl_3$) δ 9.08 (s, 1H), 8.41 (d, *J* = 8.2 Hz, 1H), 7.83 – 7.70 (m, 3H), 7.62 (t, 1H), 7.46 (d, *J* = 8.1 Hz, 2H), 1.65 (s, 9H). ^{13}C NMR (101 MHz, CD_3OD) δ 177.47, 160.05, 151.75, 146.48, 138.70, 137.68, 135.31, 133.20 (2C), 132.06, 127.42, 126.11, 123.07 (2C), 112.37, 82.19, 28.55 (3C). ^{19}F NMR (376 MHz, $CDCl_3$) δ 37.73. ESI-MS m/z: 420.1 (MH^+).

3.20 Preparation of *8-(4-((fluorosulfonyl)oxy)phenyl)-4-oxo-1,4-dihydroquinoline-3-carboxylic acid (3c).*

Treatment of tert-butyl 8-(4-((fluorosulfonyl)oxy)phenyl)-4-oxo-1,4-dihydroquinoline-3-carboxylate (**16a**) as outlined in general procedure D provided the title compound (**3c**) as a white solid. ^{1}H NMR (500 MHz, DMSO-d_6) δ 12.24 (s, 1H), 8.54 (d, *J* = 7.0 Hz, 1H), 8.46 (d, *J* = 6.6

Hz, 1H), 7.90 – 7.80 (m, 3H), 7.78 – 7.71 (m, 3H). ^{13}C NMR (126 MHz, DMSO-d_6) δ 178.83, 166.37, 148.10, 145.82, 137.51, 136.52, 133.83, 132.35, 130.58, 128.97, 126.59, 126.43, 126.37, 125.42, 122.82, 108.30. ^{19}F NMR (376 MHz, DMSO-d_6) δ 40.36. ESI-MS m/z: 364.0 (MH^+). HRMS (ESI) m/z calcd. for $C_{16}H_{11}FNO_6S^+$ (MH^+), 364.0286; found, 364.0288 (Δ = -0.65 ppm).

3.21 Preparation of *8-(3-((fluorosulfonyl)oxy)phenyl)-4-oxo-1,4-dihydroquinoline-3-carboxylic acid (3d).*

Treatment of tert-butyl 8-(3-((fluorosulfonyl)oxy)phenyl)-4-oxo-1,4-dihydroquinoline-3-carboxylate (**16b**) as outlined in general procedure D provided the title compound (**3d**) as a white solid. ^{1}H NMR (500 MHz, DMSO-d_6) δ 15.22 (s, 1H), 12.18 (s, 1H), 8.56 (s, 1H), 8.41 (d, *J* = 6.7 Hz, 1H), 7.90 – 7.78 (m, 4H), 7.76 – 7.66 (m, 2H). ^{13}C NMR (126 MHz, DMSO-d_6) δ 178.81, 166.61, 150.46, 146.01, 138.81, 137.25, 135.70, 132.12, 131.31, 131.18, 126.46, 125.90, 125.56, 123.09, 121.88, 108.09. ^{19}F NMR (376 MHz, DMSO-d_6) δ 39.03. ESI-MS m/z: 364.0 (MH^+). HRMS (ESI) m/z calcd. for $C_{16}H_{11}FNO_6S^+$ (MH^+), 364.0286; found, 364.0287 (Δ = -0.38 ppm).

3.22 Preparation of *8-(2-((fluorosulfonyl)oxy)phenyl)-4-oxo-1,4-dihydroquinoline-3-carboxylic acid (3e).*

Treatment of tert-butyl 8-(2-((fluorosulfonyl)oxy)phenyl)-4-oxo-1,4-dihydroquinoline-3-carboxylate (**16c**) as outlined in general procedure D provided the title compound (**3e**) as a white solid. ^{1}H NMR (500 MHz, DMSO-d_6) δ 15.19 (s, 1H), 12.20 (s, 1H), 8.57 (s, 1H), 8.40 (d, *J* = 8.2 Hz, 1H), 7.90 – 7.73 (m, 5H), 7.70 (t, *J* = 7.8 Hz, 1H). ^{13}C NMR (126 MHz, DMSO-d_6) δ

178.91, 166.57, 150.37, 145.86, 137.09, 137.08, 135.78, 132.81 (2C), 131.62, 126.52, 125.75, 125.52, 122.46 (2C), 108.09. ^{19}F NMR (376 MHz, DMSO-d$_6$) δ 38.99. ESI-MS m/z: 364.0 (MH$^+$). HRMS (ESI) m/z calcd. for $C_{16}H_{11}FNO_6S^+$ (MH$^+$), 364.0286; found, 364.0289 (Δ = -0.93 ppm).

3.23 Preparation of *N-benzyl-4-bromo-3-hydroxybenzamide (19a)*.

Treatment of 4-bromo-3-hydroxybenzoic acid (**17**) and phenylmethanamine (**18a**) as outlined in general procedure E provided the title compound (**19a**) as a light-yellow solid (96% yield). ^{1}H NMR (500 MHz, DMSO-d$_6$) δ 10.58 (s, 1H), 9.02 (t, J = 6.0 Hz, 1H), 7.57 (d, J = 6.4 Hz, 1H), 7.45 (s, 1H), 7.33 – 7.28 (m, 4H), 7.27 – 7.21 (m, 2H), 4.44 (d, J = 5.9 Hz, 2H). ^{13}C NMR (126 MHz, DMSO-d$_6$) δ 166.00, 154.57, 140.04, 135.57, 133.16, 128.75 (2C), 127.65(2C), 127.22, 119.25, 115.94, 113.09, 43.09. ESI-MS m/z: 306.0, 308.0 (MH$^+$).

3.24 Preparation of *4-bromo-3-hydroxy-N-phenethylbenzamide (19b)*.

Treatment of 4-bromo-3-hydroxybenzoic acid (**17**) and 2-phenylethan-1-amine (**18b**) as outlined in general procedure E provided the title compound (**19b**) as a light-yellow solid (92% yield). ^{1}H NMR (400 MHz, DMSO-d$_6$) δ 10.48 (s, 1H), 8.53 (t, *J* = 5.6 Hz, 1H), 7.55 (d, *J* = 8.2 Hz, 1H), 7.39 (d, *J* = 2.0 Hz, 1H), 7.32 – 7.14 (m, 6H), 3.45 (q, 2H), 2.82 (t, *J* = 7.4 Hz, 2H).^{13}C NMR (101 MHz, DMSO-d$_6$) δ 165.90, 154.49, 139.96, 135.83, 133.09, 129.11 (2C), 128.81 (2C), 126.56, 119.16, 115.84, 112.91, 41.35, 35.49. ESI-MS m/z: 320.1, 322.0 (MH$^+$).

3.25 Preparation of *tert-butyl 8-(4-(benzylcarbamoyl)-2-hydroxyphenyl)-4-oxo-1,4-dihydroquinoline-3-carboxylate (21a).*

Treatment of N-benzyl-4-bromo-3-hydroxybenzamide (**19a**) as outlined in general procedure F and coupling of the intermediate N-benzyl-3-hydroxy-4-(4,4,5,5-tetramethyl-1,3,2-dioxaborolan-2-yl)benzamide [**20a**, ESI-MS m/z: 354.3 (MH^+)] with tert-butyl 8-bromo-4-oxo-1,4-dihydroquinoline-3-carboxylate (**13**) provided the title compound (**21a**) as a white solid (29% yield for two steps). ESI-MS m/z: 471.1 (MH^+).

3.26 Preparation of *tert-butyl 8-(2-hydroxy-4-(phenethylcarbamoyl)phenyl)-4-oxo-1,4-dihydroquinoline-3-carboxylate (21b).*

Treatment of 4-bromo-3-hydroxy-N-phenethylbenzamide (**19b**) as outlined in general procedure F and coupling of the intermediate 3-hydroxy-N-phenethyl-4-(4,4,5,5-tetramethyl-1,3,2-dioxaborolan-2-yl)benzamide [**20b**, ESI-MS m/z: 485.4 (MH^+)] with tert-butyl 8-bromo-4-oxo-1,4-dihydroquinoline-3-carboxylate (**13**) provided the title compound (**21b**) as a white solid (15% yield for two steps). ESI-MS m/z: 485.4 (MH^+)

3.27 Preparation of *tert-butyl-8-(4-(benzylcarbamoyl)-2-((fluorosulfonyl)oxy)phenyl)-4-oxo-1,4-dihydroquinoline-3-carb-oxylate (22a).*

Treatment of tert-butyl 8-(4-(benzylcarbamoyl)-2-hydroxyphenyl)-4-oxo-1,4-dihydroquinoline-3-carboxylate (**21a**) as outlined in general procedure B provided the title compound (**22a**) as a white solid (22% yield). ^{1}H NMR (400 MHz, $CDCl_3$) δ 12.59 (s, 1H), 8.90 (s, 1H), 8.46 (d, *J* = 8.1 Hz,

1H), 8.25 – 7.81 (m, 2H), 7.82 – 7.50 (m, 2H), 7.44 (d, *J* = 7.8 Hz, 1H), 7.25 – 6.96 (m, 5H), 4.49 (s, 2H), 1.59 (s, 9H). ^{19}F NMR (376 MHz, $CDCl_3$) δ 41.25. ESI-MS m/z: 553.4 (MH^+).

3.28 Preparation of *tert-butyl-8-(2-((fluorosulfonyl)oxy)-4-(phenethylcarbamoyl)phenyl)-4-oxo-1,4-dihydro-quinoline-3-carboxylate (22b).*

Treatment of tert-butyl 8-(2-hydroxy-4-(phenethylcarbamoyl)phenyl)-4-oxo-1,4-dihydroquinoline-3-carboxylate (**21b**) as outlined in general procedure B provided the title compound (**22b**) as a white solid (27% yield). ^{1}H NMR (400 MHz, $CDCl_3$) δ 12.55 (s, 1H), 9.00 (s, 1H), 8.46 (dd, *J* = 8.3, 1.6 Hz, 1H), 7.86 (s, 1H), 7.80 (d, *J* = 7.8 Hz, 1H), 7.72 (d, *J* = 7.0 Hz, 1H), 7.62 (t, *J* = 7.8 Hz, 1H), 7.55 (d, *J* = 7.9 Hz, 1H), 7.33 (t, *J* = 7.4 Hz, 2H), 7.24 (d, *J* = 7.7 Hz, 3H), 3.72 (q, *J* = 6.7 Hz, 2H), 2.95 (t, *J* = 6.9 Hz, 2H), 1.62 (s, 9H). ^{13}C NMR (126 MHz, $CDCl_3$) δ 165.25, 160.71, 156.12, 148.04, 146.59, 138.65, 136.37, 133.69, 133.21, 130.21, 128.83 (4C), 126.75, 126.59, 125.60, 123.30, 120.11, 117.60, 113.23, 106.69, 41.43, 35.56, 31.62, 28.23 (3C), 25.86. ^{19}F NMR (376 MHz, $CDCl_3$) δ 41.74. ESI-MS m/z: 567.4 (MH^+).

3.29 Preparation of *8-(4-(benzylcarbamoyl)-2-((fluorosulfonyl)oxy)phenyl)-4-oxo-1,4-dihydroquinoline-3-carboxylic acid (3f).*

Treatment of tert-butyl-8-(4-(benzylcarbamoyl)-2-((fluorosulfonyl)oxy)phenyl)-4-oxo-1,4-dihydroquinoline-3-carb-oxylate (**22a**) as outlined in general procedure D provided the title compound (**3f**) as a white solid. ^{1}H NMR (400 MHz, DMSO-d_6) δ 12.30 (d, *J* = 7.0 Hz, 1H), 9.47 (t, *J* = 6.0 Hz, 1H), 8.53 (d, *J* = 6.8 Hz, 1H), 8.48 (dd, *J* = 8.1, 1.5 Hz, 1H), 8.32 (s, 1H), 8.27 (dd, *J* = 8.0, 1.6 Hz, 1H), 7.93 – 7.84 (m, 2H), 7.76 (t, 1H), 7.38 – 7.33 (m, 4H), 7.32 – 7.24

(m, 1H), 4.57 (d, *J* = 5.9 Hz, 2H). ^{13}C NMR (126 MHz, DMSO-d_6) δ 178.31, 165.86, 163.68, 147.63, 145.37, 139.21, 137.29, 137.01, 135.86, 133.71, 131.32, 128.51, 128.40 (2C), 127.30 (2C), 126.98, 126.45, 125.99, 125.29, 124.99, 120.98, 107.93, 48.60. ^{19}F NMR (376 MHz, DMSO-d_6) δ 40.94. ESI-MS m/z: 497.3 (MH^+). HRMS (ESI) m/z calcd. for $C_{24}H_{18}FN_2O_7S^+$ (MH^+), 497.0813; found, 497.0817 (Δ = -0.75 ppm).

3.30 Preparation of *8-(2-((fluorosulfonyl)oxy)-4-(phenethylcarbamoyl)phenyl)-4-oxo-1,4-dihydroquinoline-3-carboxylic acid (3g).*

Treatment of tert-butyl-8-(2-((fluorosulfonyl)oxy)-4-(phenethylcarbamoyl)phenyl)-4-oxo-1,4-dihydro-quinoline-3-carboxylate (**22b**) as outlined in general procedure D provided the title compound (**3g**) as a white solid. ^{1}H NMR (500 MHz, CD_3CN) δ 14.90 (brs, 1H), 10.37 (brs, 1H), 8.52 (t, *J* = 7.5 Hz, 2H), 8.05 (s, 1H), 8.00 (dd, *J* = 7.9, 1.7 Hz, 1H), 7.81 (d, *J* = 7.3 Hz, 1H), 7.72 – 7.64 (m, 2H), 7.41 (t, *J* = 6.1 Hz, 1H), 7.32 (p, *J* = 7.1, 6.7 Hz, 4H), 7.23 (t, 1H), 3.66 (q, *J* = 6.9 Hz, 2H), 2.95 (t, *J* = 7.2 Hz, 2H). ^{13}C NMR (126 MHz, DMSO-d_6) δ 178.32, 165.88, 163.64, 147.57, 145.38, 139.35, 137.57, 137.00, 135.90, 133.66, 131.16, 128.71 (2C), 128.41 (2C), 128.35, 126.44, 126.21, 125.99, 125.31, 124.99, 120.84, 107.93, 41.03, 34.93. ^{19}F NMR (376 MHz, DMSO-d_6) δ 40.85. ESI-MS m/z: 511.3 (MH^+). HRMS (ESI) m/z calcd. for $C_{25}H_{20}FN_2O_7S^+$ (MH^+), 511.0970; found, 511.0972 (Δ = -0.44 ppm).

IV. X-RAY CRYSTALLOGRAPHY

Table S1. X-ray diffraction data collection and refinement statistics.

Complex	TDP1-XZ503 **2a**	TDP1-IB01 **2e**	TDP1-IB02 **2f**	TDP1-XZ520 **3a**	TDP1-IB06 **3b**	TDP1-IB03 **3c**	TDP1-IB05 **3e**	TDP1-IB09 **3f**
Compound concentration	3.2 mM	25 mM	12 mM	15.9 mM	7.2 mM	78 mM	28 mM	5.4 mM
Diffraction source	SER-CAT, 22_ID	SER-CAT, 22-BM	SER-CAT, 22-BM	SER-CAT, 22-BM	SER-CAT, 22-ID	SER-CAT, 22-BM	SER-CAT, 22-BM	SER-CAT 22-ID
Wavelength (Å)	1.0000	1.0000	1.000	1.0000	1.0000	1.000	1.0000	1.0000
Space group	$P2_12_12_1$	$P2_12_12_1$	$P2_12_12_1$	$P2_12_12_1$	$P2_12_12_1$	$P2_12_12_1$	$P2_12_12_1$	$P2_12_12_1$
a=, b=, c= (Å)	49.54, 105.36, 193.74	49.96, 105.02, 193.04	49.79, 104.81, 193.18	50.07, 105.59, 194.67	50.02, 105.18, 193.63	49.90, 104.99, 193.49	49.94, 104.53, 192.84	50.02, 104.75,193.24
α, β, γ = (°)	90	90	90	90	90	90	90	90
Resolution range (Å)	50-1.88 (1.91-1.88)	50-1.83 (1.86-1.83)	50-1.86 (1.89-1.85)	50-1.66 (1.69-1.66)	50-1.62 (1.65-1.62)	50-1.93 (1.96-1.93)	50-1.65 (1.68-1.65)	50-1.62 (1.65-1.62)
Total No. of reflections	1151097	617907	633847	814736	649141	572656	843854	624220
Total No. of Unique reflections	83844	89424	87230	121959	123313	77695	121541	122336
Completeness (%)	100 (100)	98.8 (94.4)	100 (100)	99.7 (100)	94.4 (92.5)	100 (100)	99.6 (96.9)	94.2 (89.4)
Multiplicity	13.7 (13.2)	6.9 (6.4)	7.3 (6.6)	3.3 (3.2)	5.3 (5.4)	7.4 (7.0)	6.9 (5.8)	5.2 (5.2)
$I/(\sigma)I$	3.5 (40.4	18.5 (2.1)	18.5 (2.0)	42.2 (3.3)	20.6 (1.9)	16.6 (2.0)	25 (2.0)	24.7 (2.0)
CC1/2 in highest resolution shell	0.880	0.865	0.773	0.831	0.802	0.777	0.872	0.803
R_{sym}	0..109 (0.785)	0.084 (0.608)	0.117 (0.882)	0.068 (0.697)	0.067 (0.666)	0.124 (0.968)	0.076 (0.611)	0.061 (0.670)
R_{pim}	0.030 (0.221)	0.034 (0.252)	0.046 (0.364)	0.035 (0.298)	0.031 (0.299)	0.049 (0.395)	0.031 (0.269)	0.027 (0.297)
Resolution Range (Å)	50-1.88 (1.92-1.88)	43.85-1.83 (1.86-1.83)	43.80-1.84 (1.89-1.85)	34.63-1.66 (1.70-1.66)	45.17-1.62 (1.66-1.62)	50-1.93 (1.95-1.93)	40.55-1.65 (1.69-1.65)	45.14-1.62 (1.64-1.62)
Final R_{work}	0.182 (0.239)	0.163 (0.203)	0.163 (0.210)	0.192 (0.249)	0.172 (0.218)	0.162 (0.227)	0.164 (0.236)	0.160 (0.219)
Final R_{free}	0.225 (0.285)	0.192 (0.258)	0.193 (0.268)	0.227 (0.274)	0.203 (0.243)	0.204 (0.261)	0.188 (0.260)	0.184 (0.286)
No. of non H-atoms/Average B-factor ($Å^2$)								
Protein chain A	3640/25.0	3639/24.9	3658/25.7	3626/27.0	3684/19.6	3644/25.1	3643/23.9	3656/21.2
Protein chain B	3619/30.3	3597/31.9	3667/32.2	3625/34.2	3639/27.8	3640/32.6	3684/29.5	3607/26.7
Water		571/36.9	597/37.2	627/40.5	721/34.7	632/37.4	676/37.3	755/37.0
Ethylene glycol	40/37.9	8/20.2	32/34.7	20/36.1	14/31.6	32/33.6	32/32.5	32/30.2
Polyethylene glycol	-	-	7/54.2	-	32/27.8	14/33.1	14/26.6	-
DMSO	-	-	8/60.6	-	4/44.8	8/70.8	8/68.2	4/58.6
Compound chain A	15/37.1	52/33.7	21/52.0	20/39.2	53/30.6	24/28.9	25/48.1	54/44.1
Compound chain B	15/39.5	70/50.5	21/47.17	-	53/34.5	24/36.5	25/45.1	27/34.7
R.m.s.d from ideal								
Bond lengths	0.006	0.006	0.007	0.006	0.006	0.007	0.006	0.018

Bond angles	0.8	0.8	0.9	0.8	0.9	0.9	0.8	1.4
Ramachandran plots								
% Favored	98.3	97.5	97.8	97.8	97.8	97.9	97.9	97.6
% Allowed	1.6	2.5	2.1	2.1	2.1	2.1	2.1	2.4
% Outliers	0.1	0	0.1	0.1	0.1	0	0	0
MolProbity Validation								
All-Atom Contacts Clash score	3.16 (98th percentile)	3.33 (98th percentile)	4.16 (98th percentile)	3.8 (97th percentile)	3.67 (97th percentile)	4.04 (98th percentile)	3.87 (97th percentile)	3.24 (98th percentile)
Protein Geometry, MolProbity Score	1.11 (99th percentile)	1.17 (99th percentile)	1.24 (99th percentile)	1.24 (98th percentile)	1.20 (98th percentile)	1.20 (99th percentile)	1.17 (99th percentile)	1.20 (98th percentile)
PDB deposition code	6DJG	8UV1	8UZV	6MYZ	8V0C	8UZZ	8V0B	9B3B

V. Dose-dependent curves of compound 3f with TDP1 in different pre-incubation time.

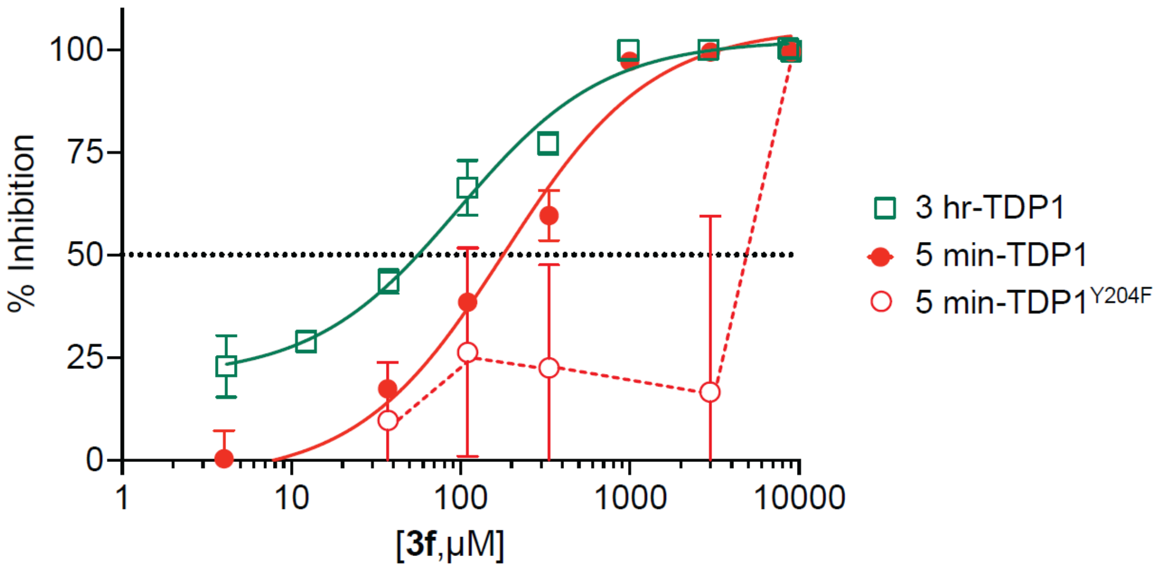

Figure S1. Overlay of dose-dependent curves of compound **3f** with TDP1 (5 min pre-incubation), TDP1 (3 hr pre-incubation) and TDP1^{Y204F} (5 min pre-incubation).

VI. ^{1}H and ^{13}C NMR Spectra of Compounds 2a – f and 3a – g.

Compound 2a, ^{1}H NMR, 500 MHz, DMSO-d_6

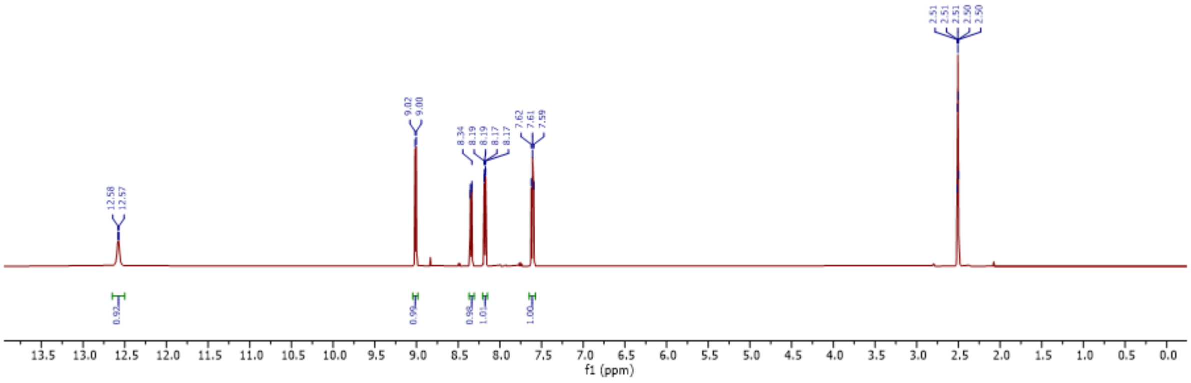

Compound 2a, ^{13}C NMR, 126 MHz, DMSO-d_6

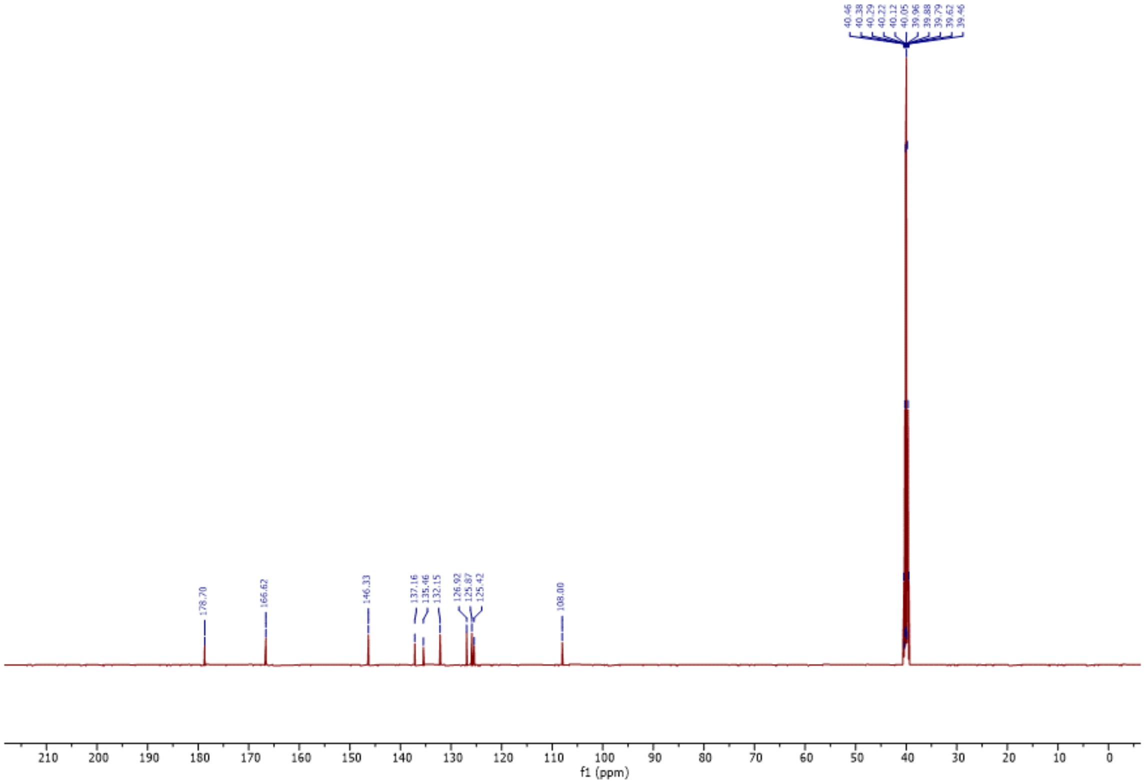

Compound 2b, ^{1}H NMR, 400 MHz, DMSO-d$_6$

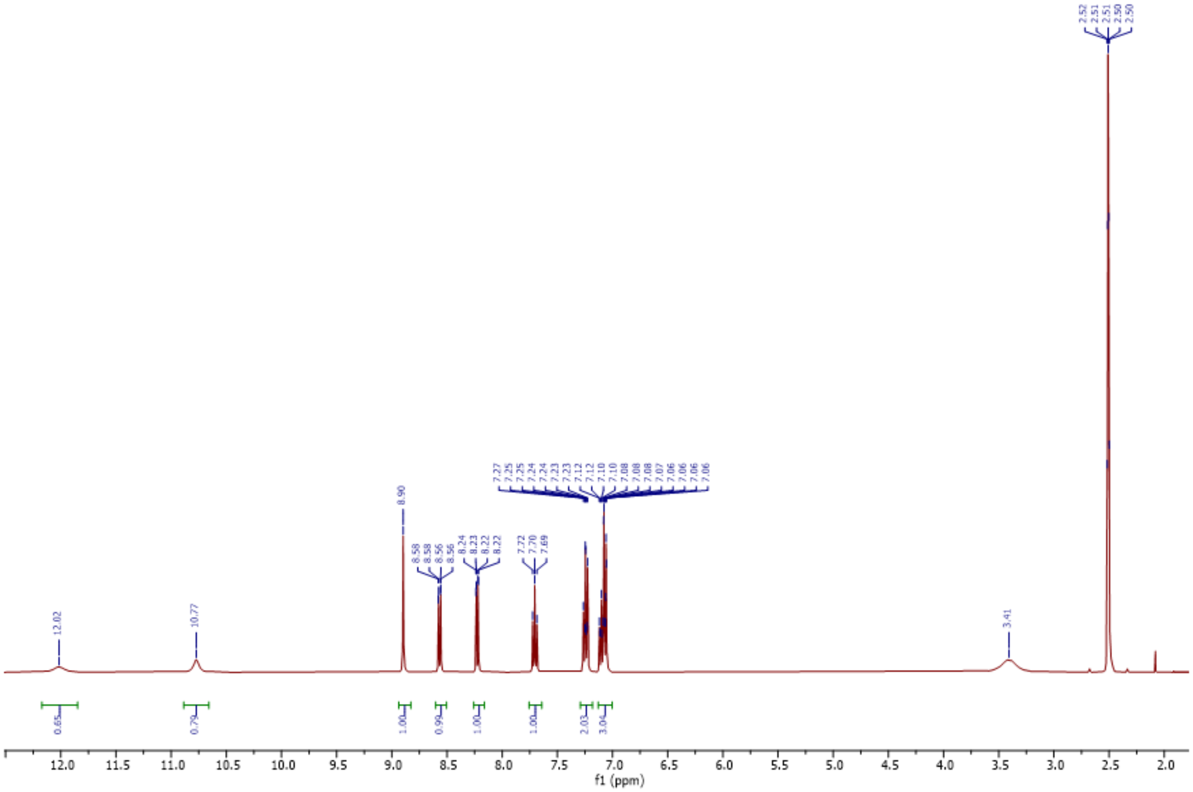

Compound 2b, ^{13}C NMR, 101 MHz, DMSO-d$_6$

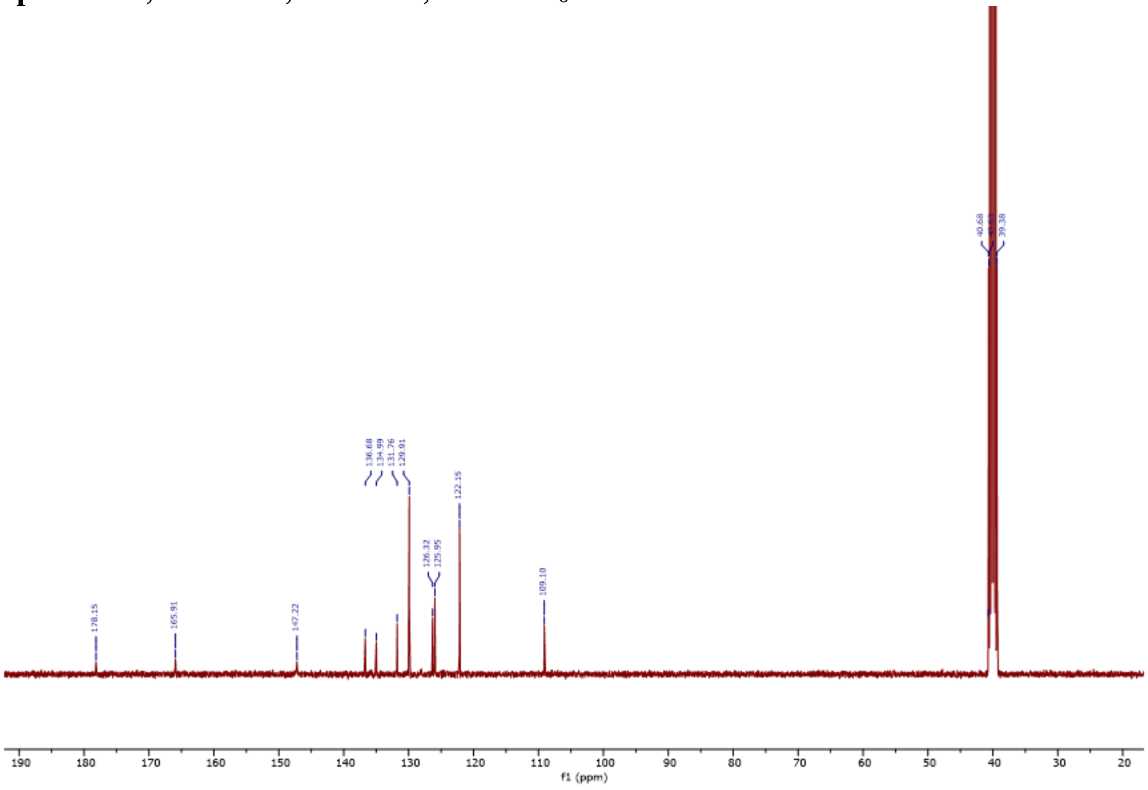

Compound 2c, ^{1}H NMR, 500 MHz, DMSO-d$_6$

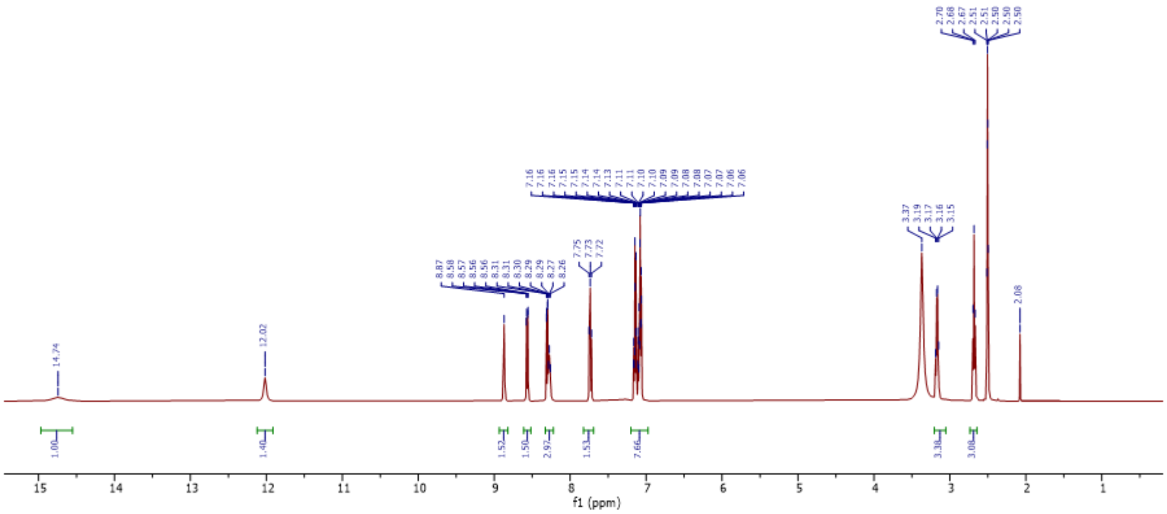

Compound 2c, ^{13}C NMR, 101 MHz, DMSO-d$_6$

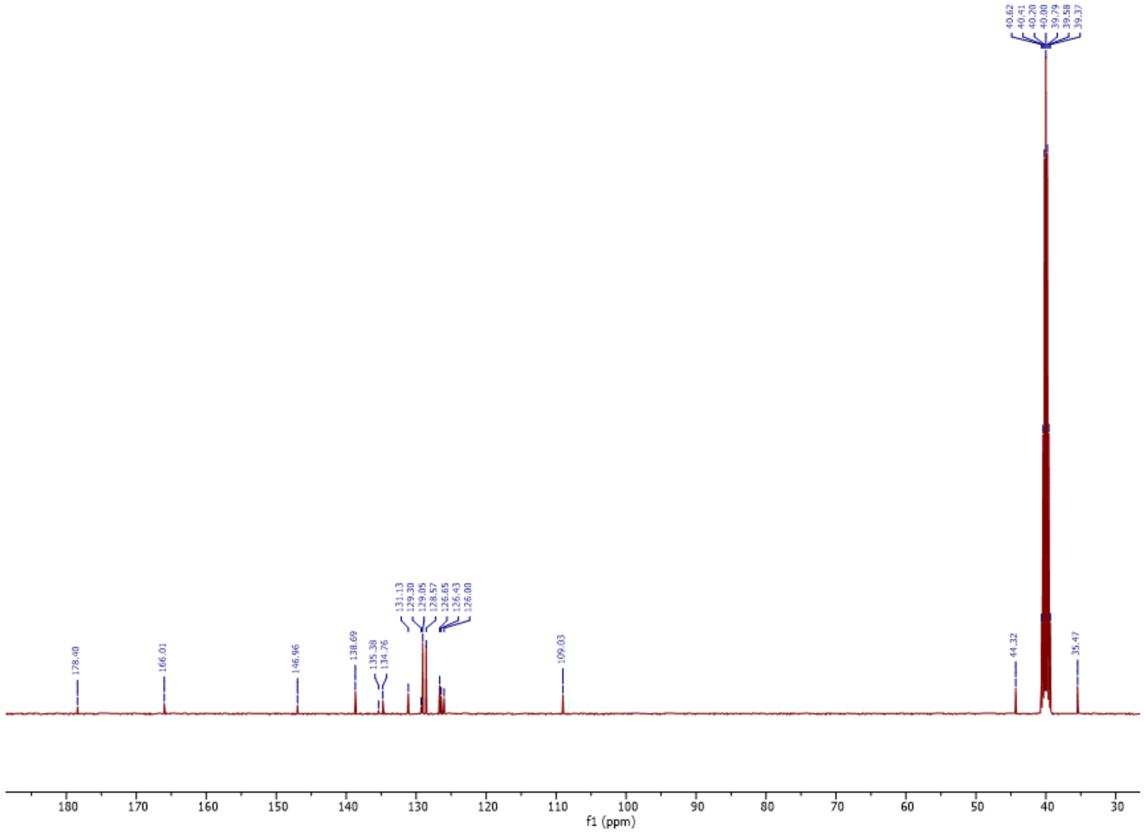

Compound 2d, ^{1}H NMR, 400 MHz, DMSO-d$_6$

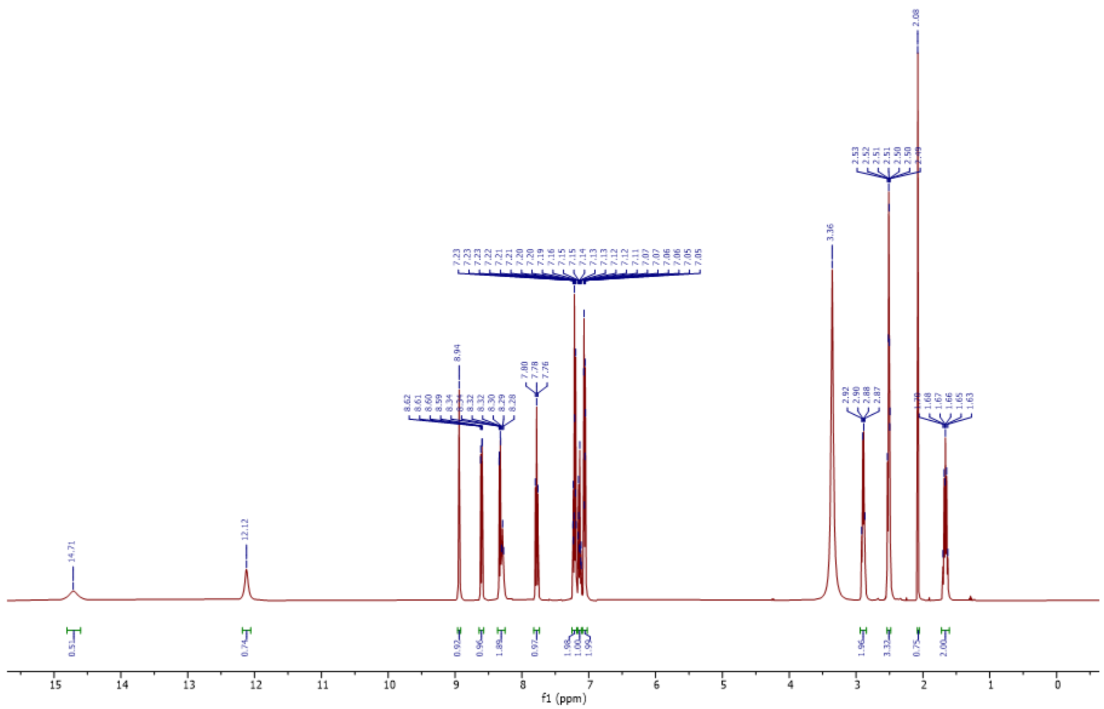

Compound 2d, ^{13}C NMR, 101 MHz, DMSO-d$_6$

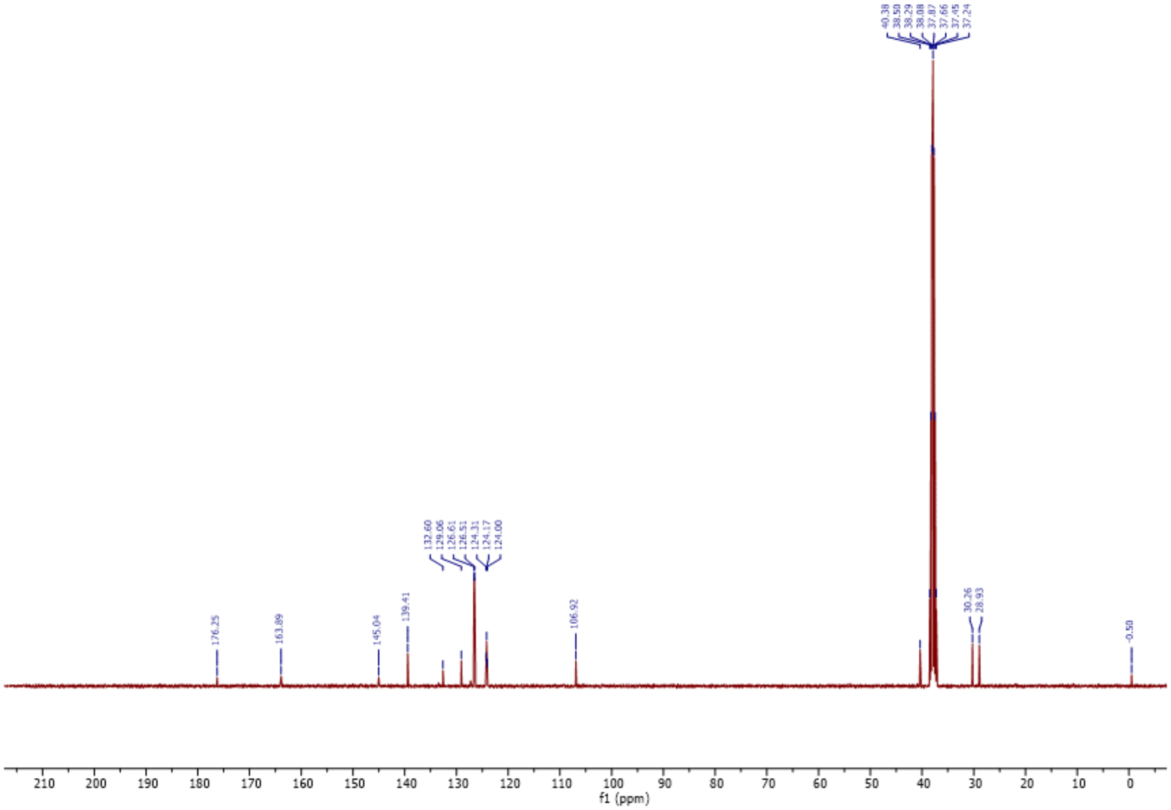

Compound 2e, ^{1}H NMR, 500 MHz, DMSO-d$_6$

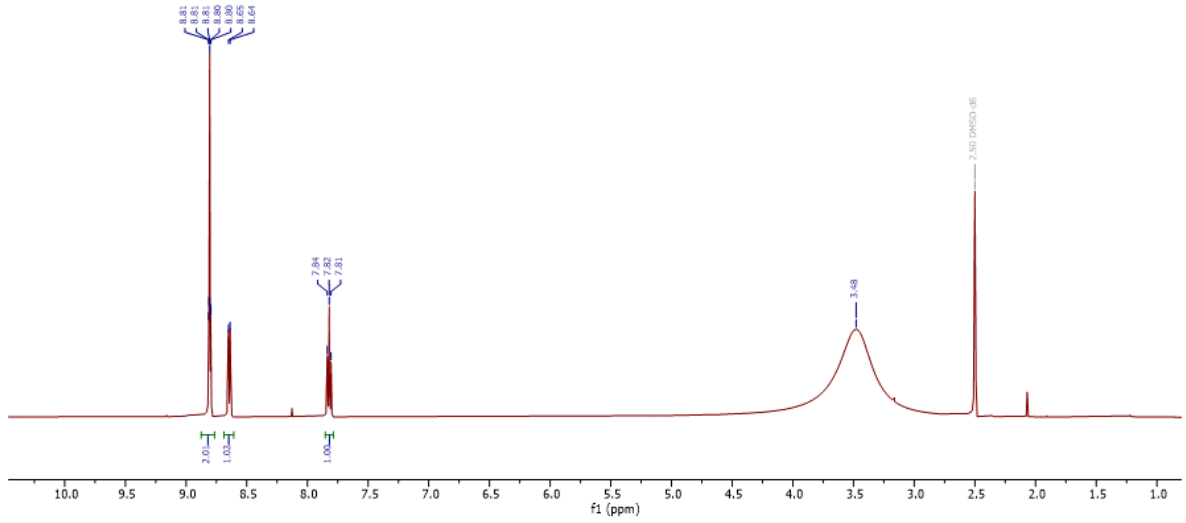

Compound 2e, ^{13}C NMR, 126 MHz, DMSO-d$_6$

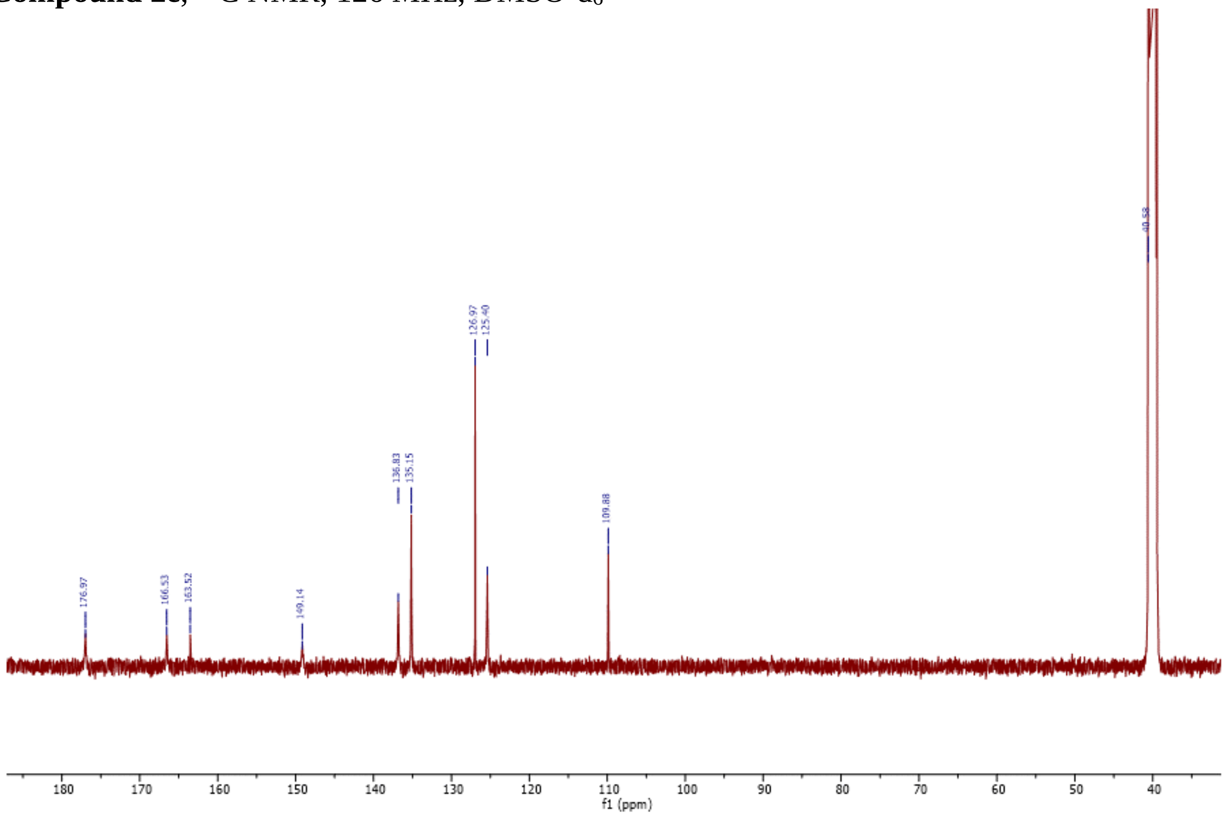

Compound 2f, ^{1}H NMR, 500 MHz, DMSO-d$_6$

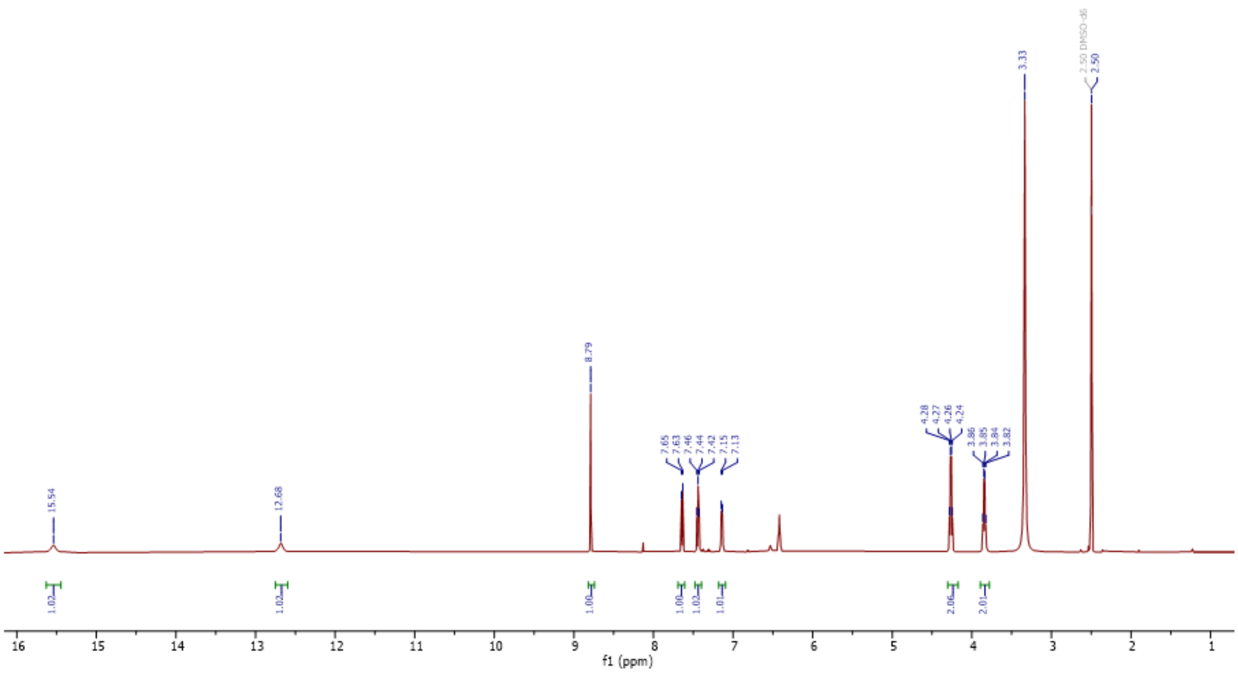

Compound 2f, ^{13}C NMR, 126 MHz, DMSO-d$_6$

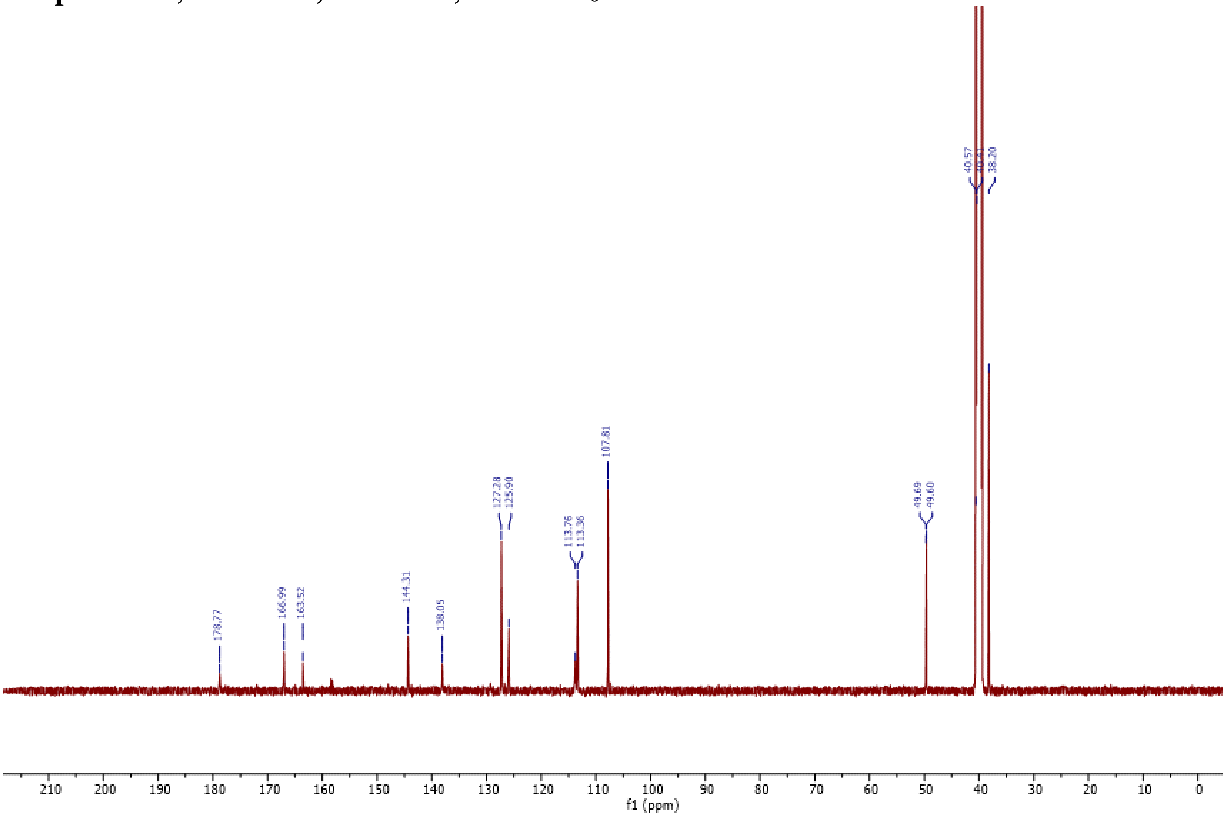

Compound 3a, ^{1}H NMR, 400 MHz, DMSO-d$_6$

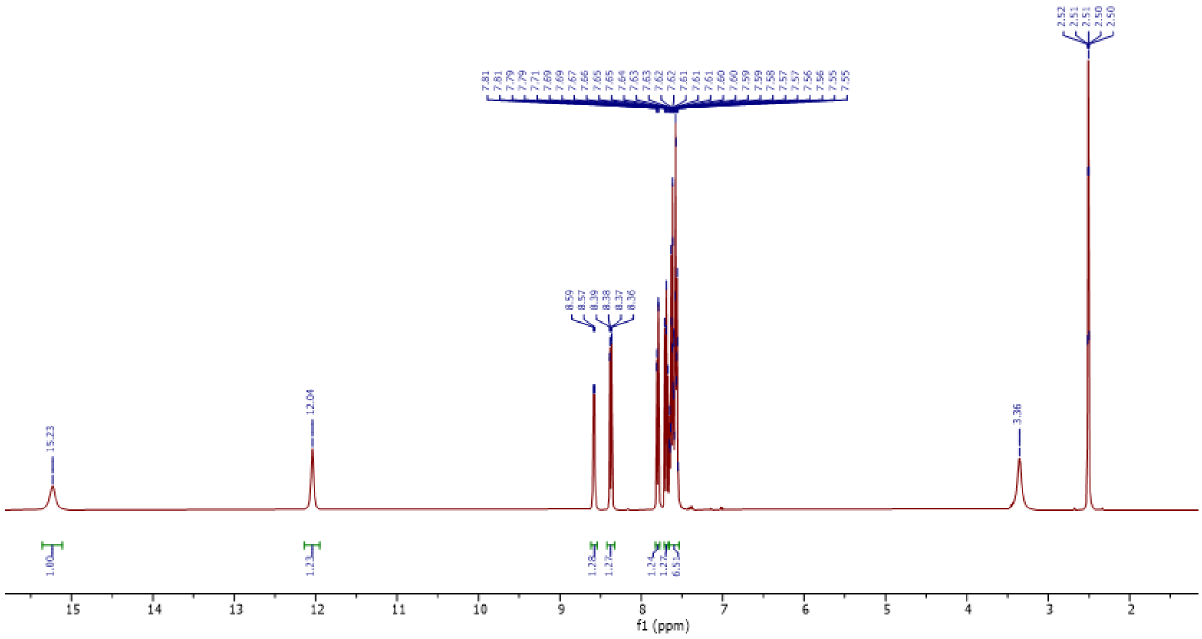

Compound 3a, ^{13}C NMR, 101 MHz, DMSO-d$_6$

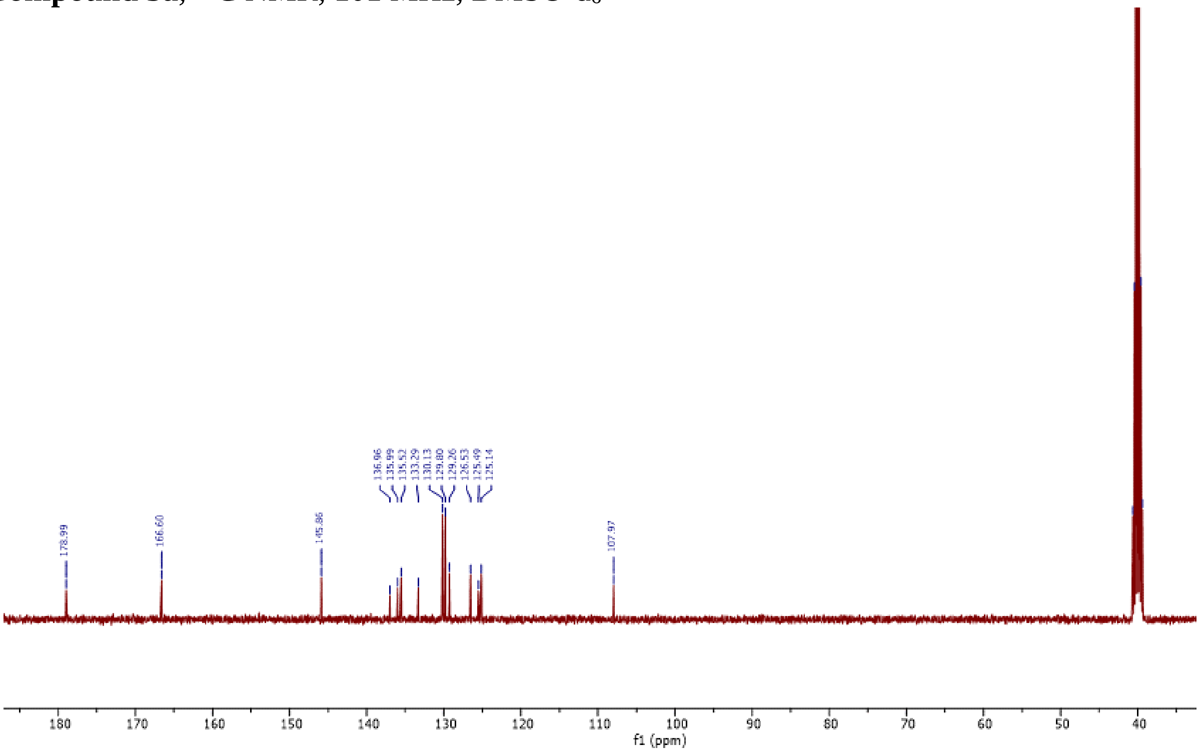

Compound 3b, ^{1}H NMR, 400 MHz, DMSO-d$_6$

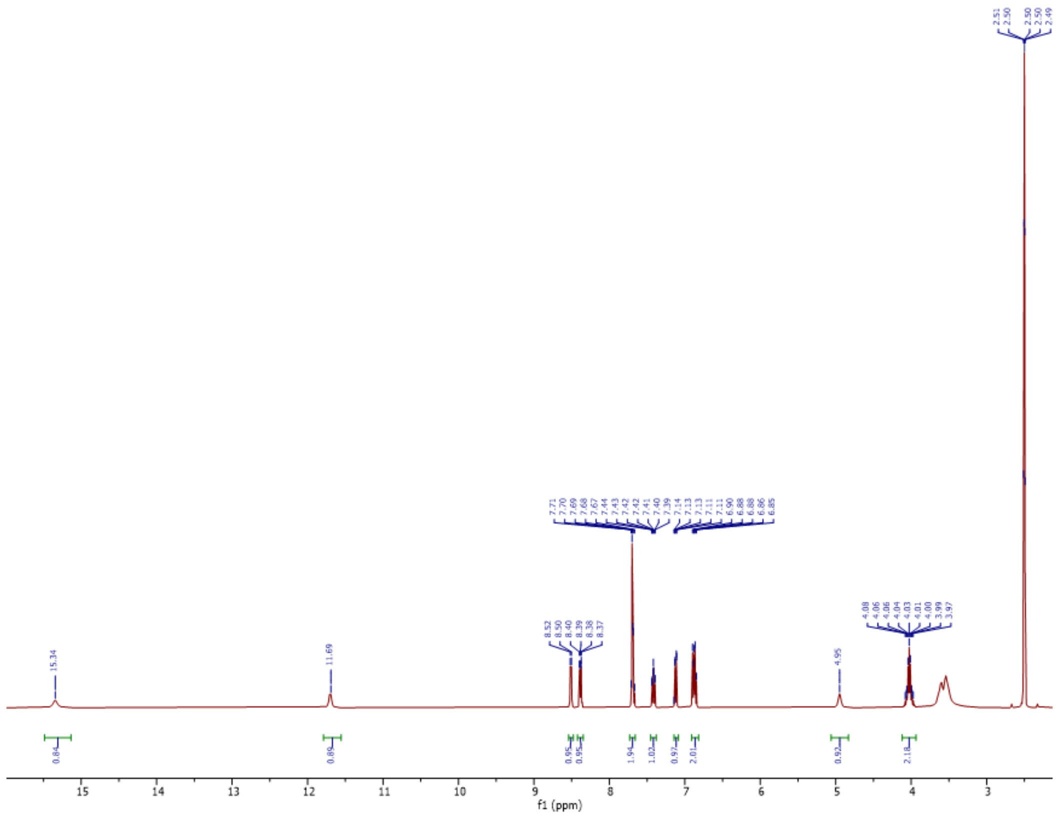

Compound 3b, ^{13}C NMR, 101 MHz, DMSO-d$_6$

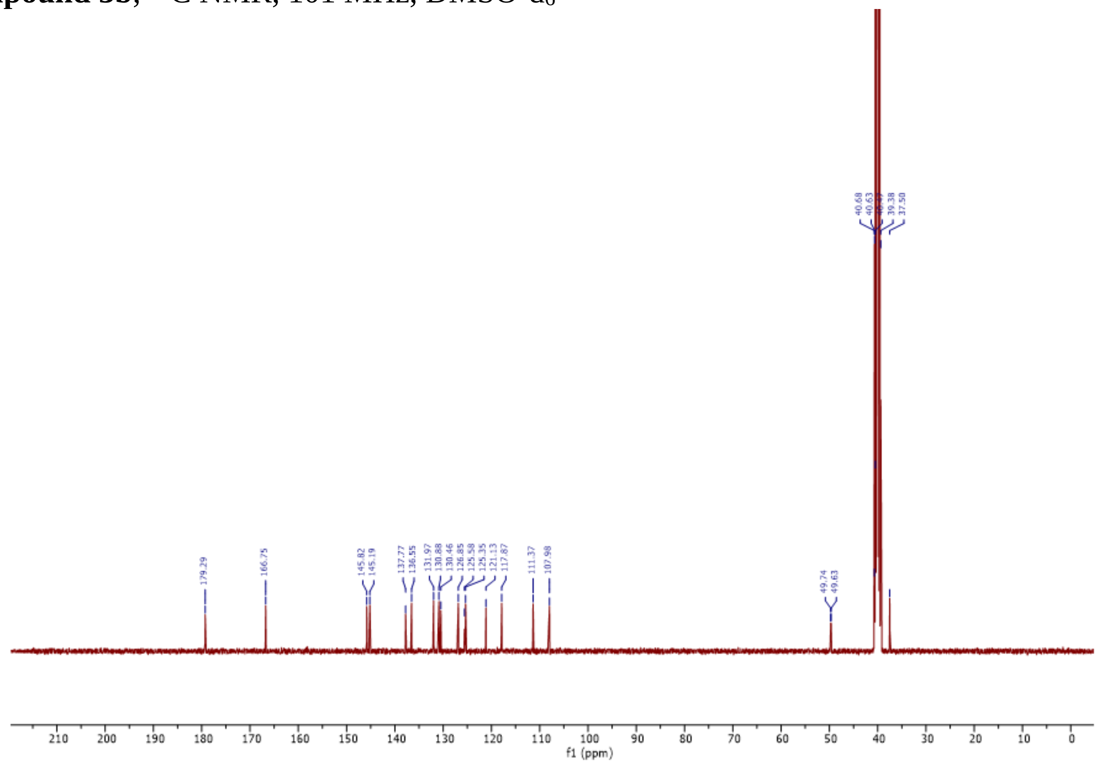

Compound 3c, ^{1}H NMR, 500 MHz, DMSO-d$_6$

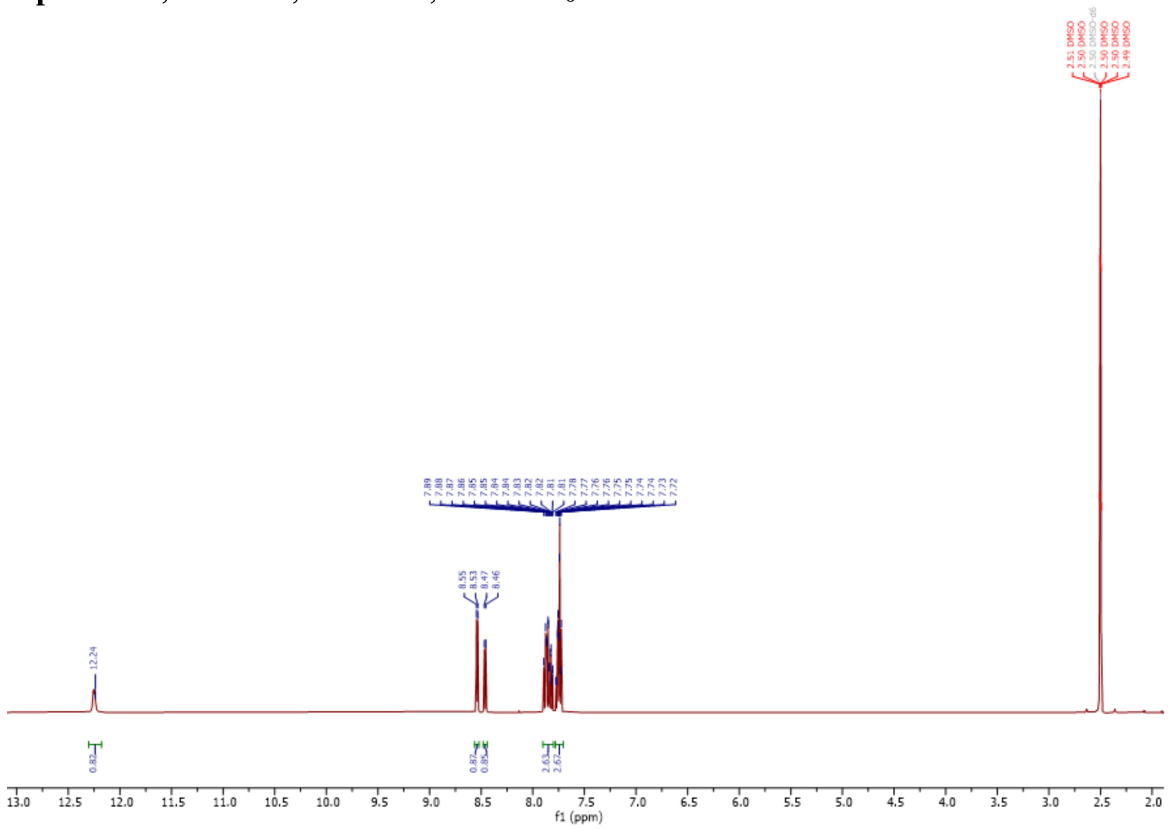

Compound 3c, ^{13}C NMR, 126 MHz, DMSO-d$_6$

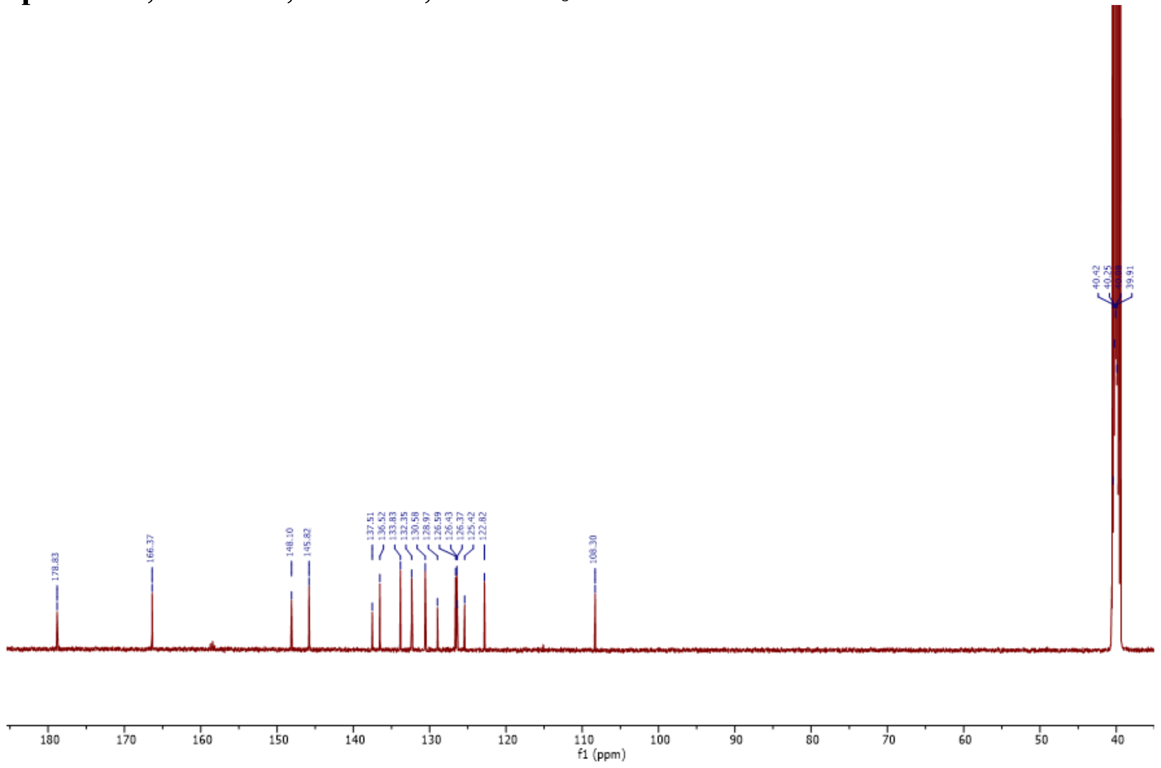

Compound 3d, ^{1}H NMR, 500 MHz, DMSO-d_6

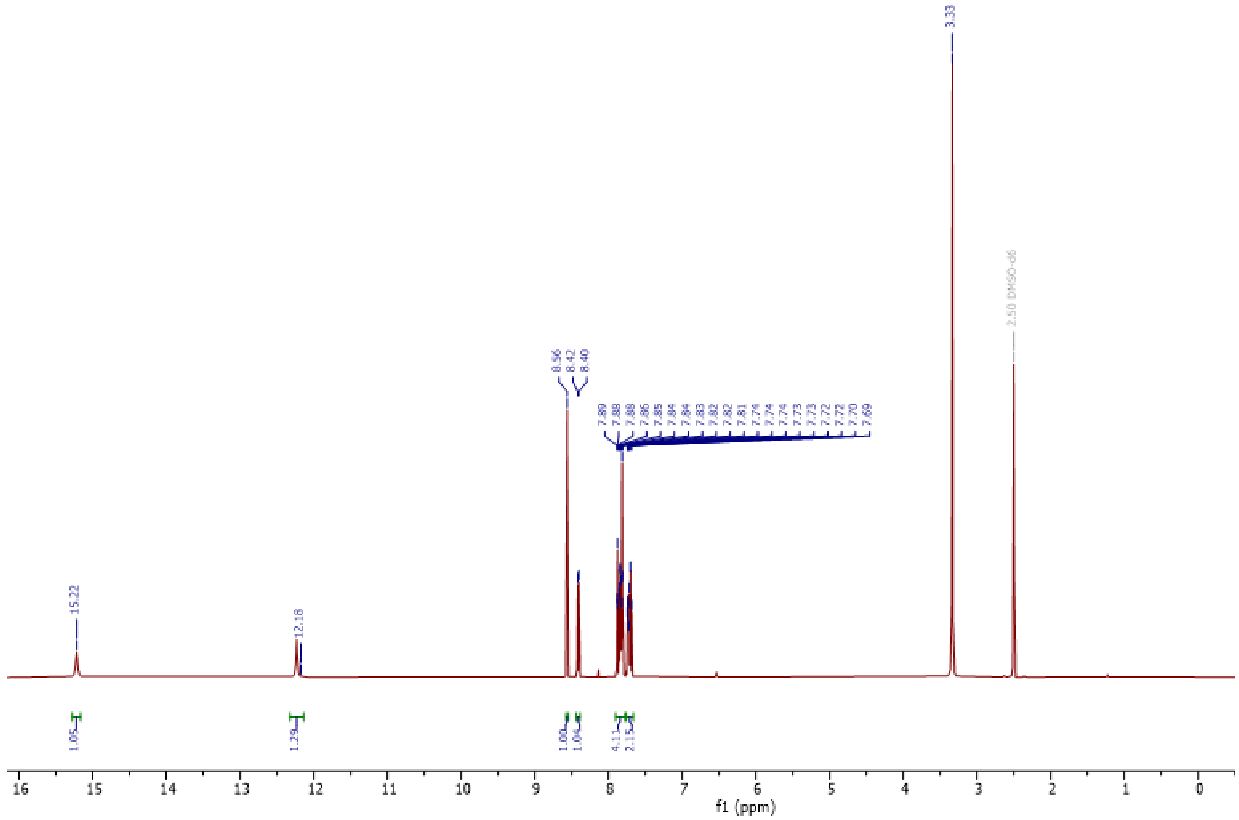

Compound 3d, ^{13}C NMR, 126 MHz, DMSO-d_6

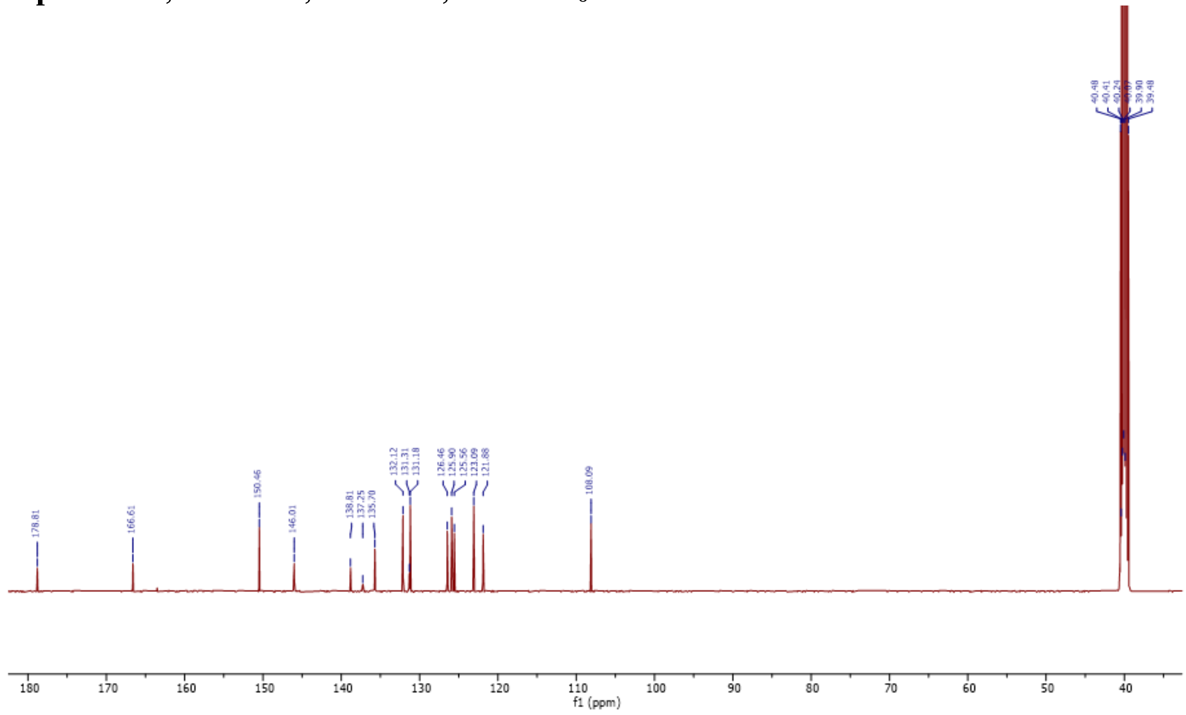

Compound 3e, ^{1}H NMR, 500 MHz, DMSO-d$_6$

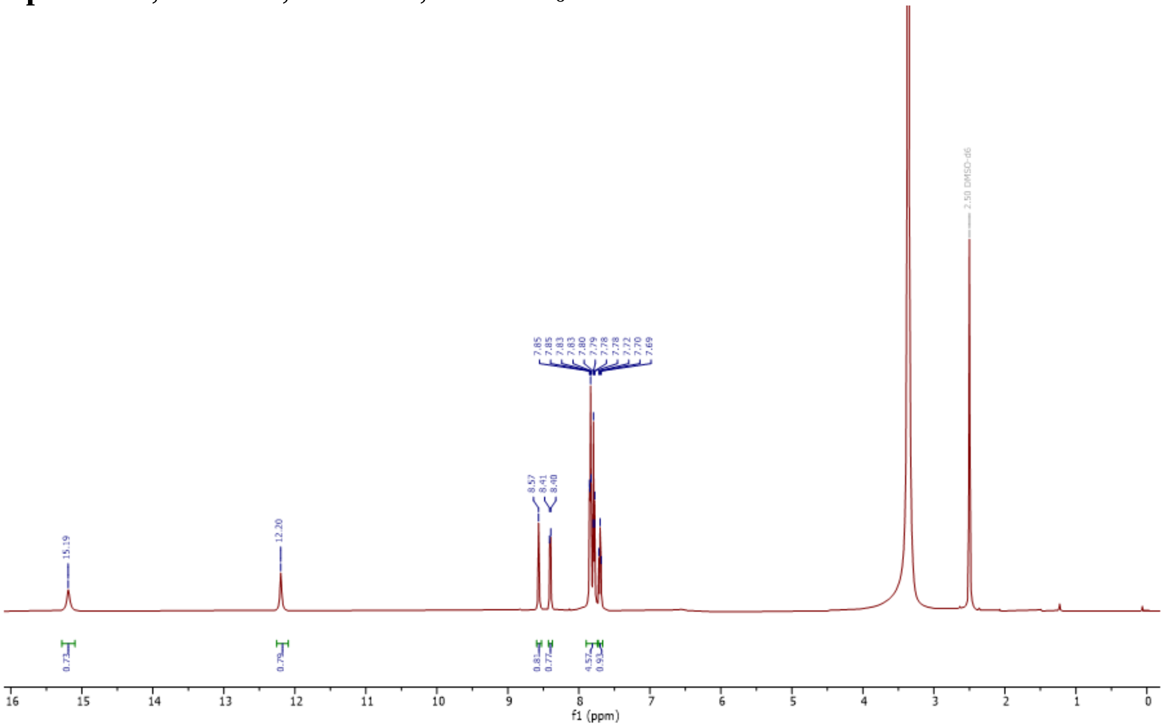

Compound 3e, ^{13}C NMR, 126 MHz, DMSO-d$_6$

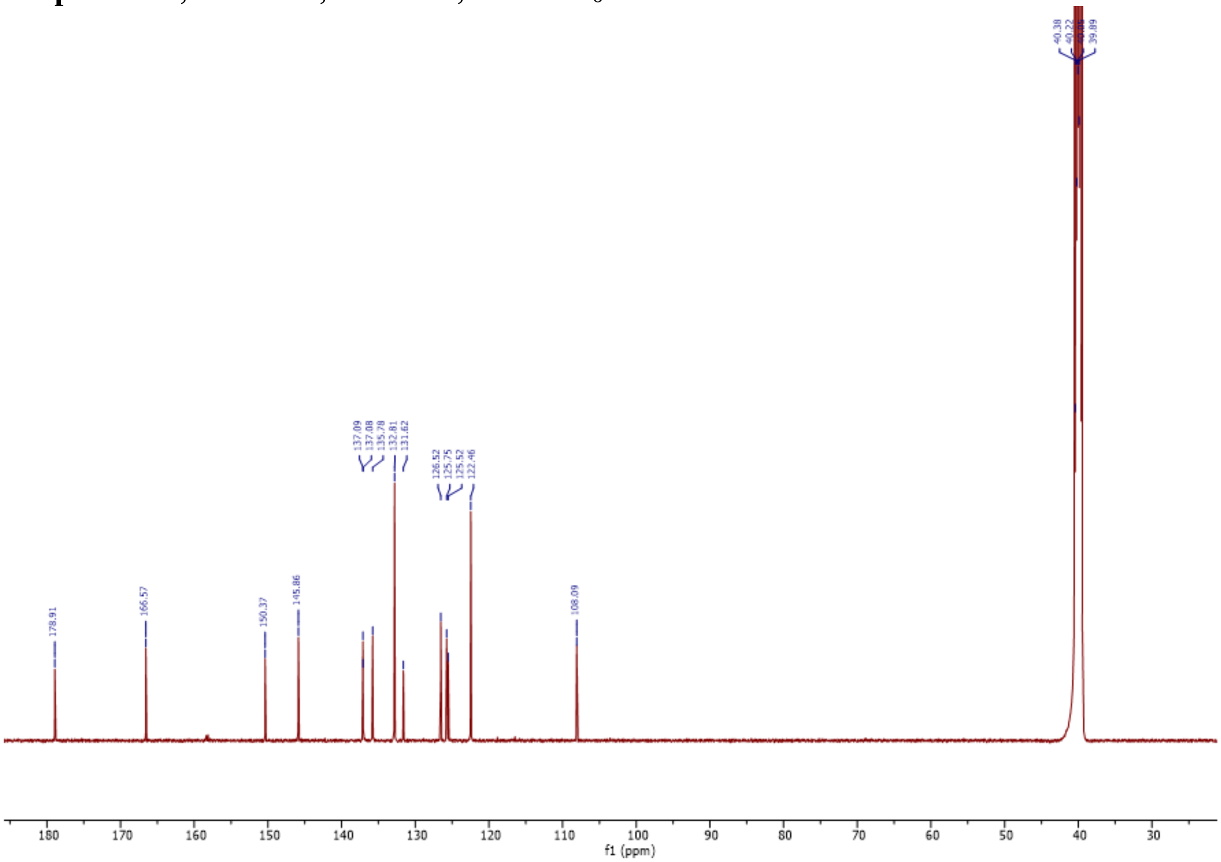

Compound 3f, ^{1}H NMR, 400 MHz, DMSO-d_6

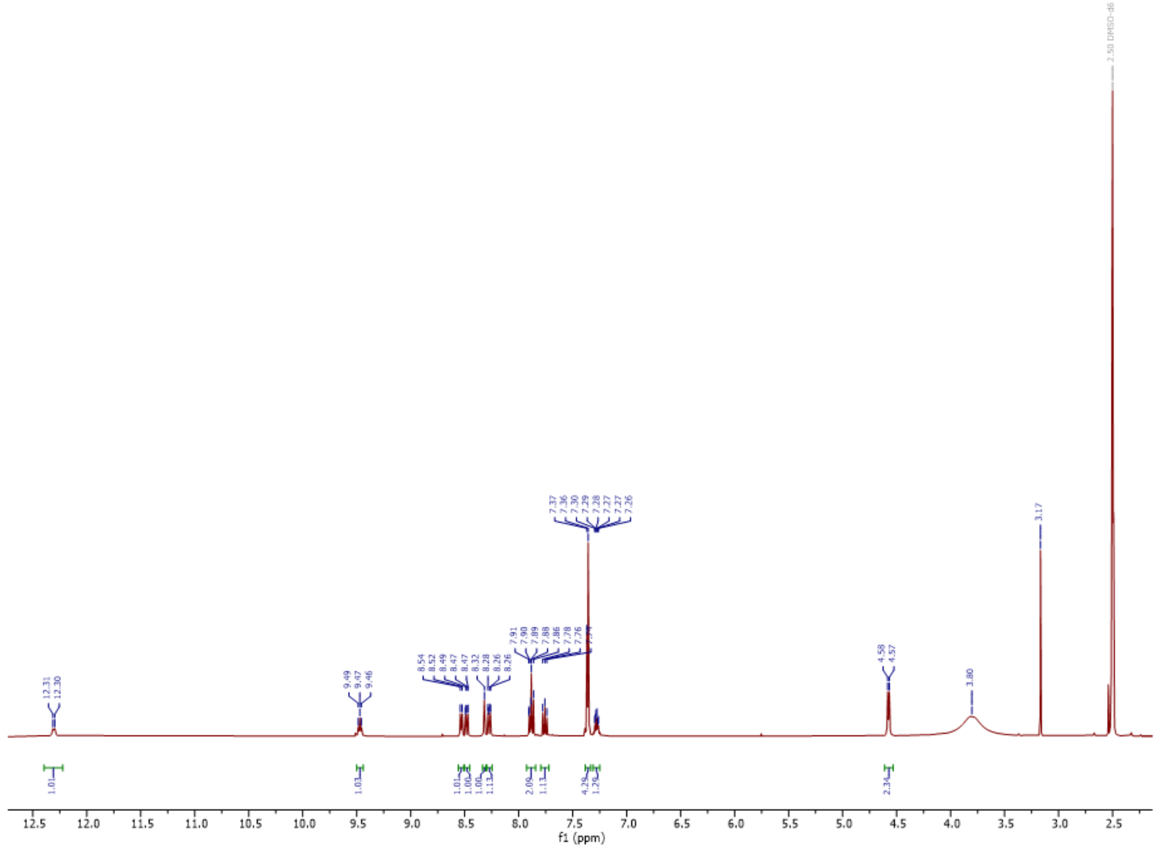

Compound 3f, ^{13}C NMR, 126 MHz, DMSO-d_6

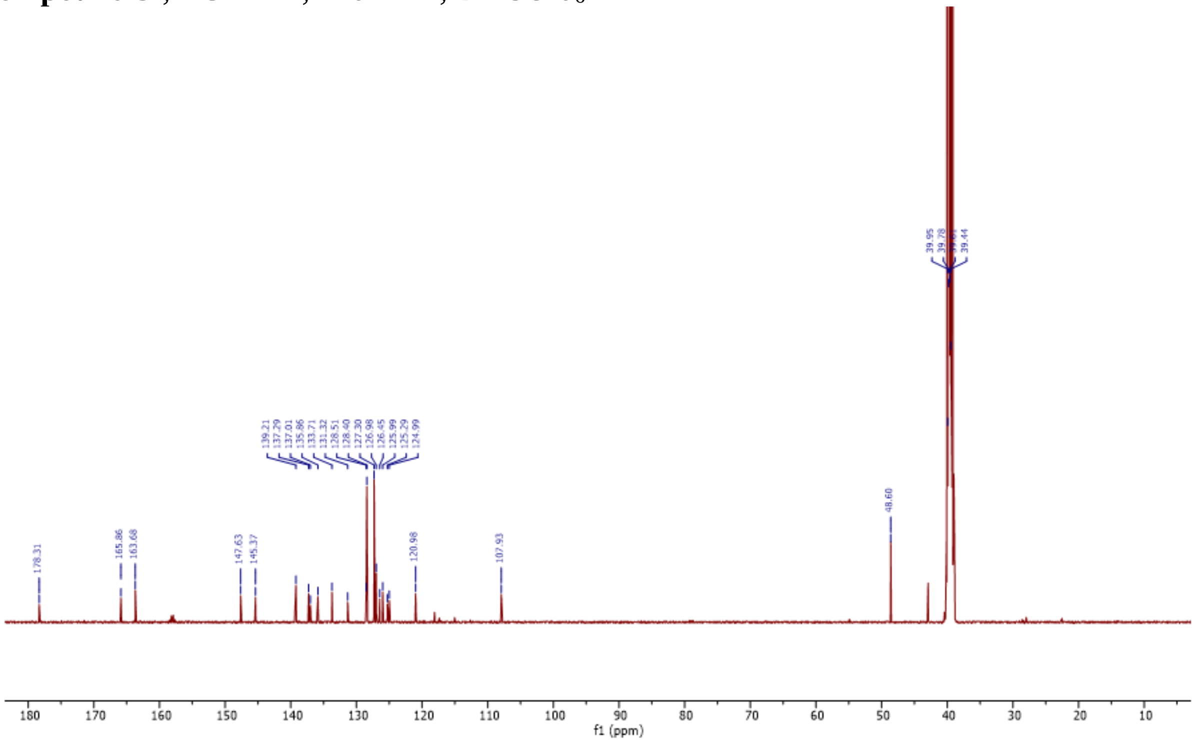

Compound 3g, ^{1}H NMR, 500 MHz, DMSO-d_6

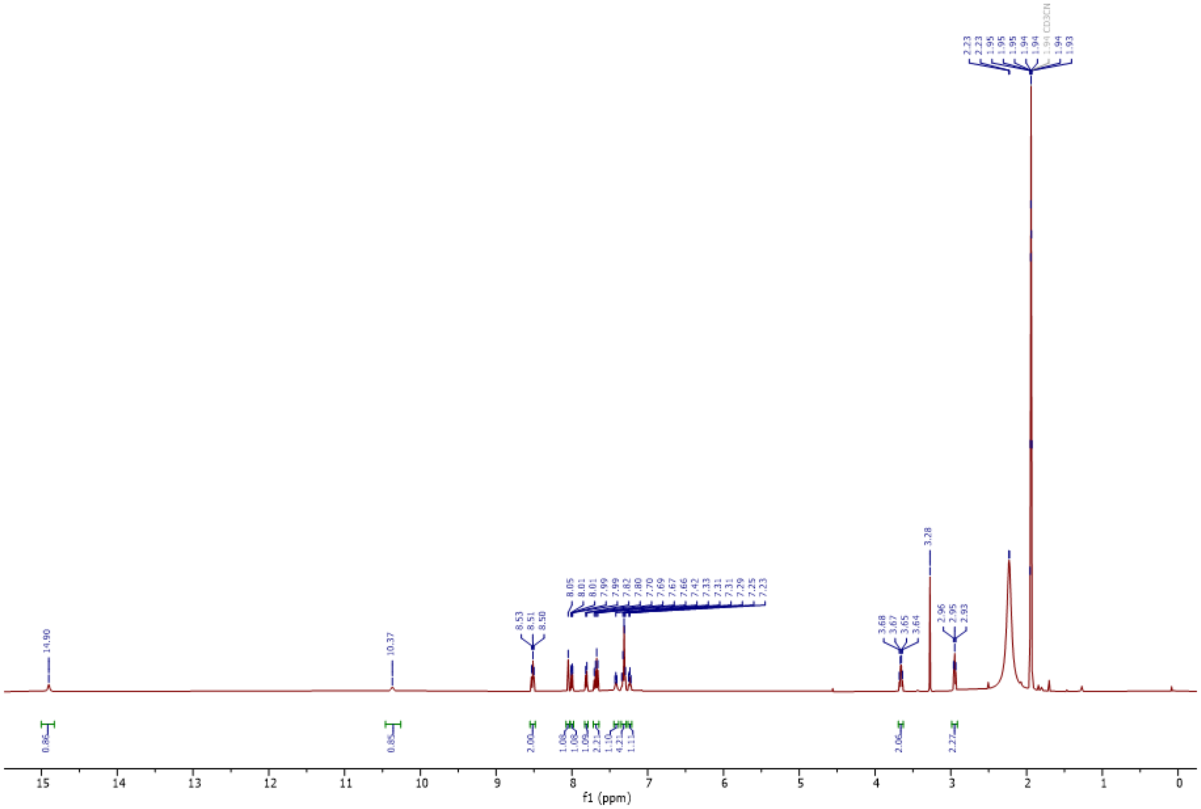

Compound 3g, ^{13}C NMR, 126 MHz, DMSO-d_6

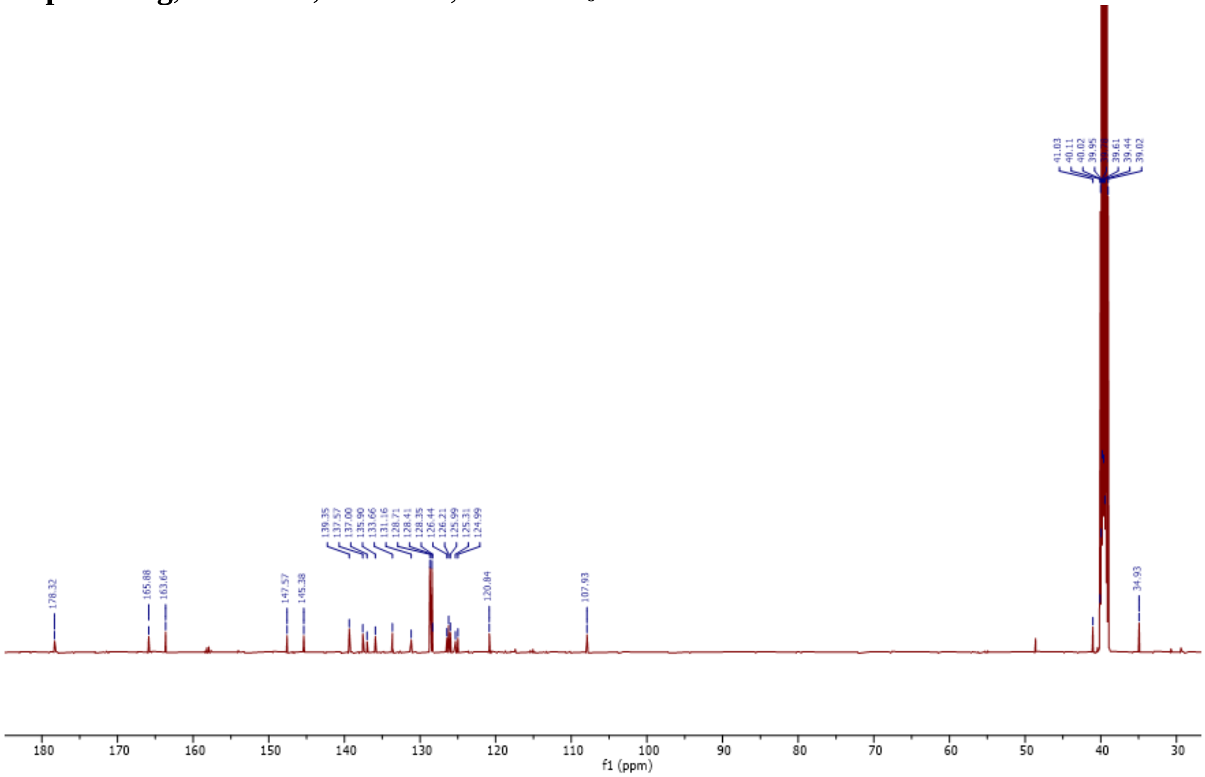

VII. Analytical HPLC of compounds 2a – f and 3a – g

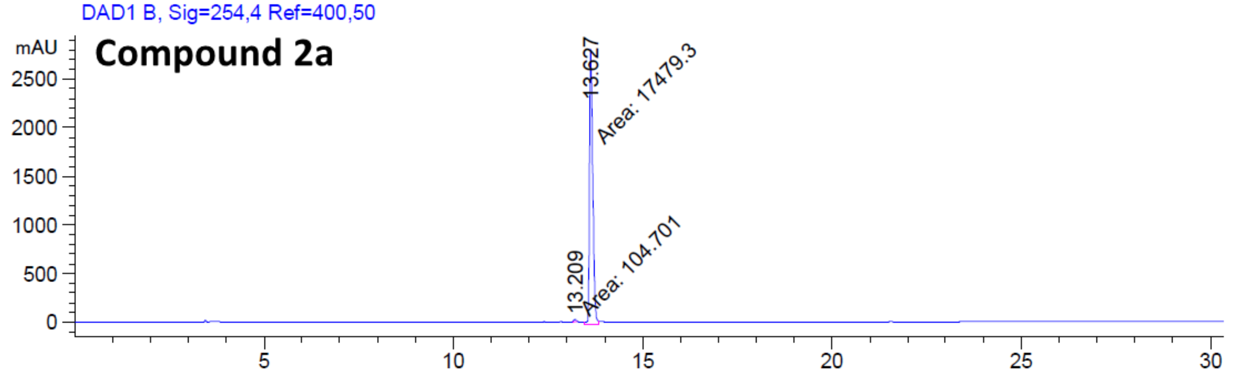

Signal 2: DAD1 B, Sig=254,4 Ref=400,50

Peak #	RetTime [min]	Type	Width [min]	Area [mAU*s]	Height [mAU]	Area %
1	13.209	MM	0.0660	104.70054	26.42572	0.5954
2	13.627	MM	0.1029	1.74793e4	2830.08643	99.4046
Totals :				1.75840e4	2856.51214	

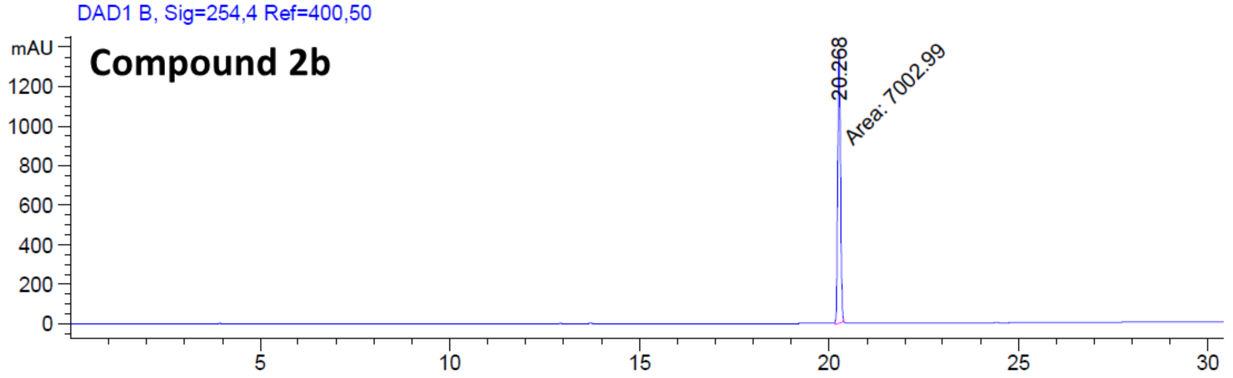

Signal 2: DAD1 B, Sig=254,4 Ref=400,50

Peak #	RetTime [min]	Type	Width [min]	Area [mAU*s]	Height [mAU]	Area %
1	20.268	MM	0.0843	7002.98779	1385.04285	100.0000
Totals :				7002.98779	1385.04285	

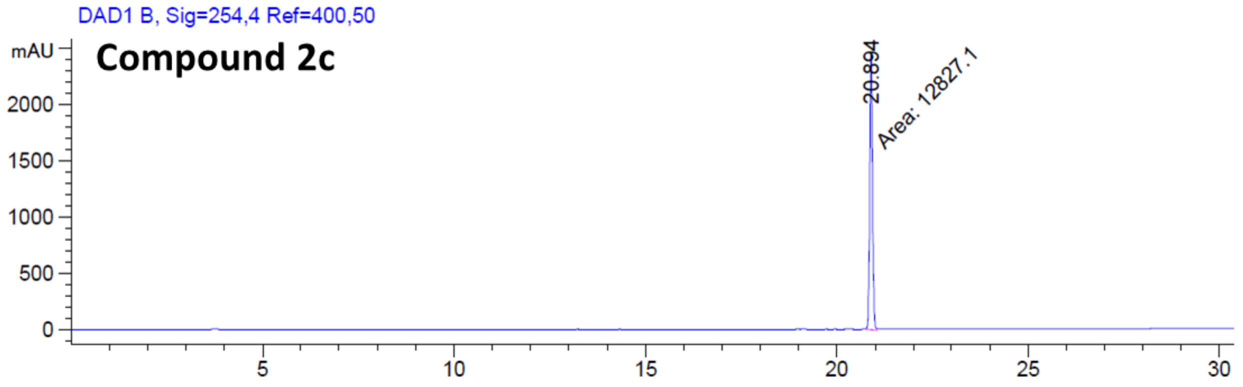

Signal 2: DAD1 B, Sig=254,4 Ref=400,50

Peak #	RetTime [min]	Type	Width [min]	Area [mAU*s]	Height [mAU]	Area %
1	20.894	MM	0.0865	1.28271e4	2472.25342	100.0000

Totals : 1.28271e4 2472.25342

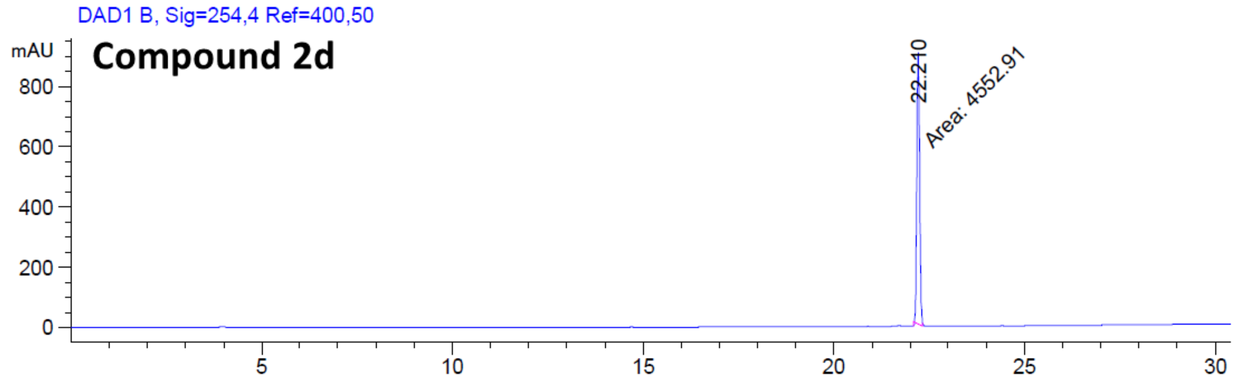

Signal 2: DAD1 B, Sig=254,4 Ref=400,50

Peak #	RetTime [min]	Type	Width [min]	Area [mAU*s]	Height [mAU]	Area %
1	22.210	MM	0.0841	4552.91406	902.51831	100.0000

Totals : 4552.91406 902.51831

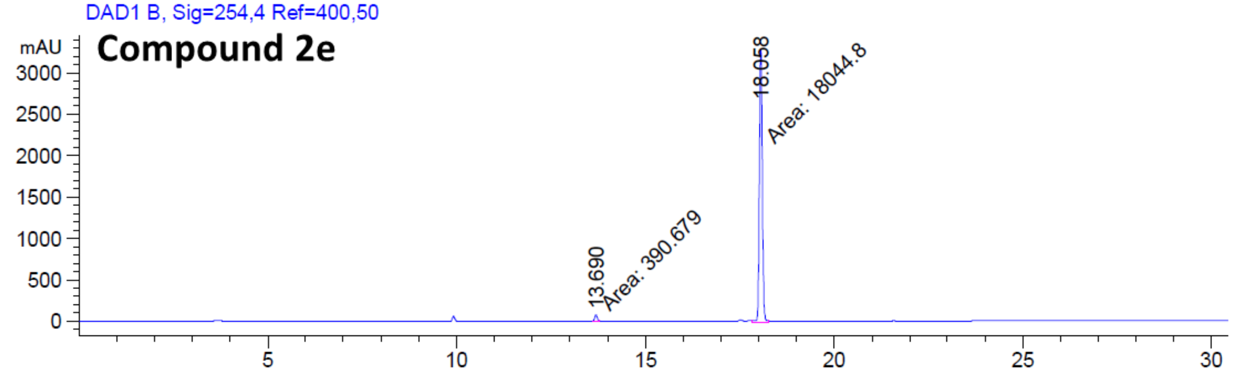

Signal 2: DAD1 B, Sig=254,4 Ref=400,50

Peak #	RetTime [min]	Type	Width [min]	Area [mAU*s]	Height [mAU]	Area %
1	13.690	MM	0.0823	390.67874	79.12749	2.1192
2	18.058	MM	0.0911	1.80448e4	3301.32495	97.8808
Totals :				1.84355e4	3380.45245	

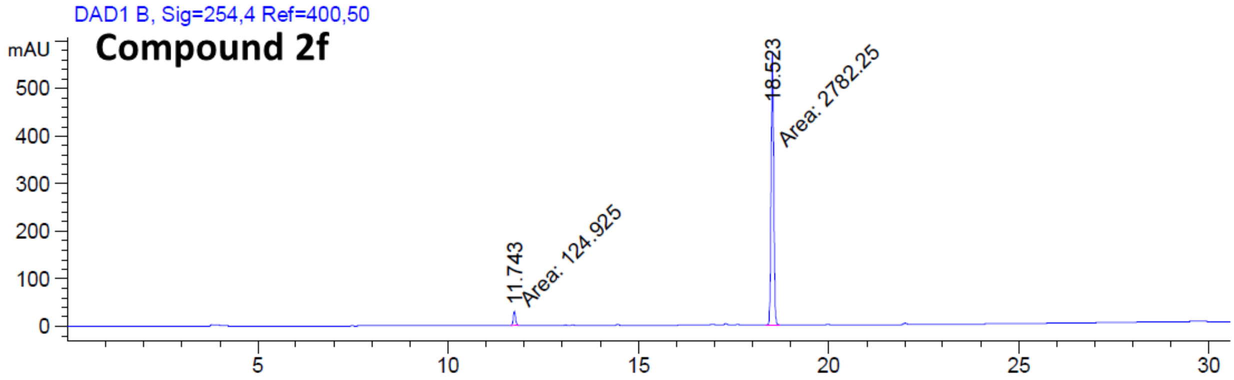

Signal 2: DAD1 B, Sig=254,4 Ref=400,50

Peak #	RetTime [min]	Type	Width [min]	Area [mAU*s]	Height [mAU]	Area %
1	11.743	MM	0.0686	124.92487	30.34530	4.2971
2	18.523	MM	0.0803	2782.25342	577.72827	95.7029
Totals :				2907.17829	608.07358	

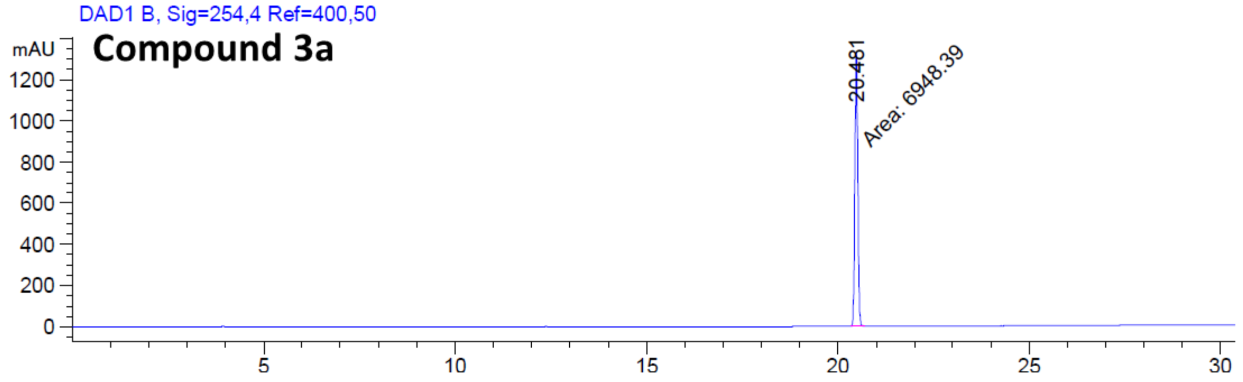

Signal 2: DAD1 B, Sig=254,4 Ref=400,50

Peak #	RetTime [min]	Type	Width [min]	Area [mAU*s]	Height [mAU]	Area %
1	20.481	MM	0.0867	6948.39404	1335.13306	100.0000

Totals : 6948.39404 1335.13306

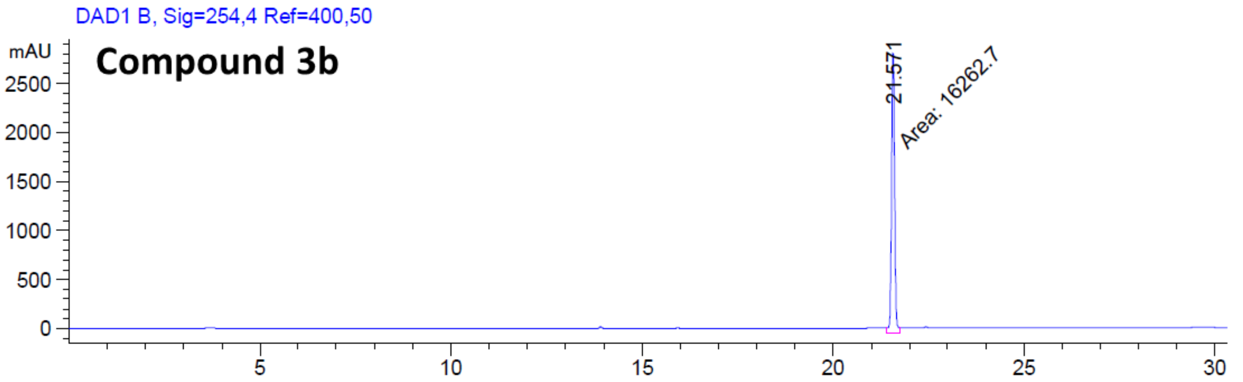

Signal 2: DAD1 B, Sig=254,4 Ref=400,50

Peak #	RetTime [min]	Type	Width [min]	Area [mAU*s]	Height [mAU]	Area %
1	21.571	MM	0.0946	1.62627e4	2866.27222	100.0000

Totals : 1.62627e4 2866.27222

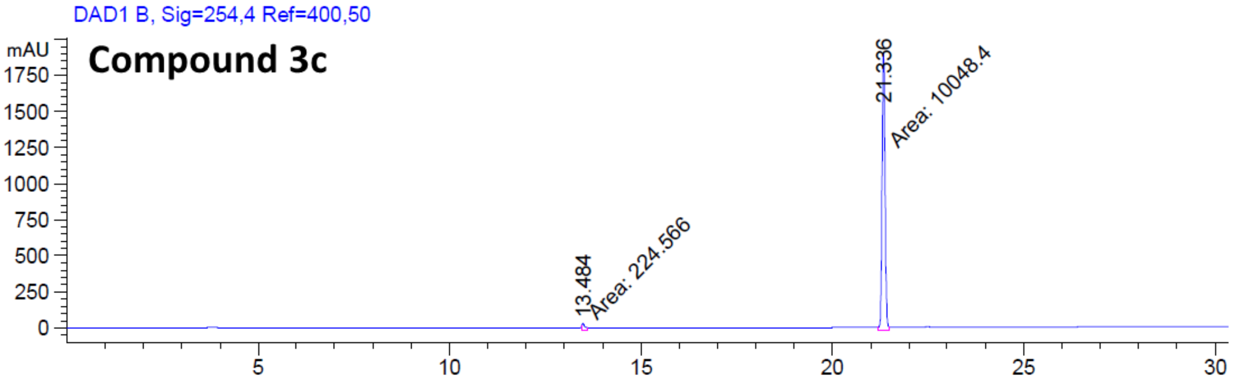

Signal 2: DAD1 B, Sig=254,4 Ref=400,50

Peak #	RetTime [min]	Type	Width [min]	Area [mAU*s]	Height [mAU]	Area %
1	13.484	MM	0.0878	224.56583	42.63567	2.1860
2	21.336	MM	0.0868	1.00484e4	1928.34753	97.8140
Totals :				1.02730e4	1970.98321	

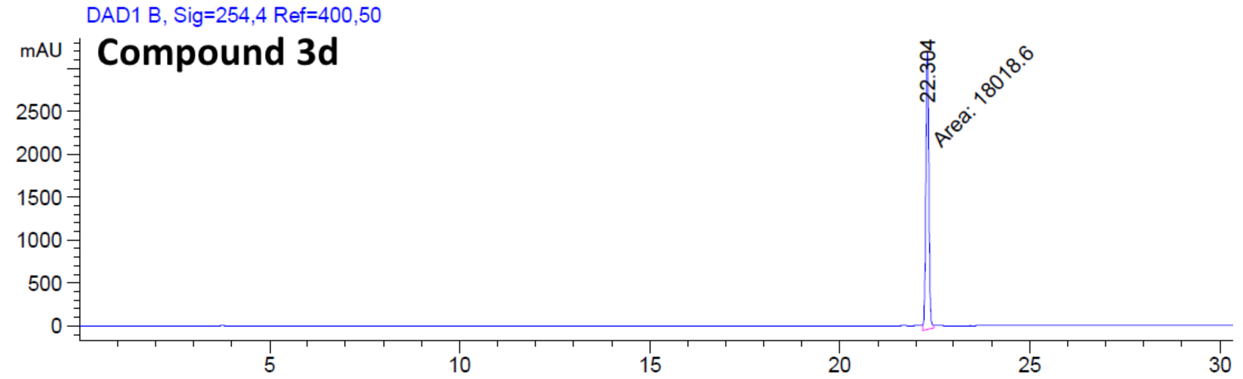

Signal 2: DAD1 B, Sig=254,4 Ref=400,50

Peak #	RetTime [min]	Type	Width [min]	Area [mAU*s]	Height [mAU]	Area %
1	22.304	MM	0.0930	1.80186e4	3230.42163	100.0000
Totals :				1.80186e4	3230.42163	

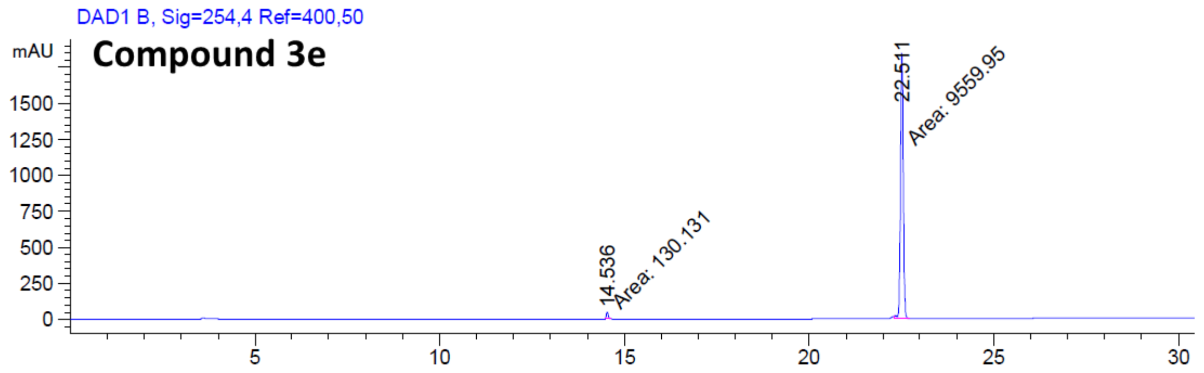

Signal 2: DAD1 B, Sig=254,4 Ref=400,50

Peak #	RetTime [min]	Type	Width [min]	Area [mAU*s]	Height [mAU]	Area %
1	14.536	MM	0.0553	130.13074	39.23249	1.3429
2	22.511	MM	0.0864	9559.95215	1844.39929	98.6571
Totals :				9690.08289	1883.63179	

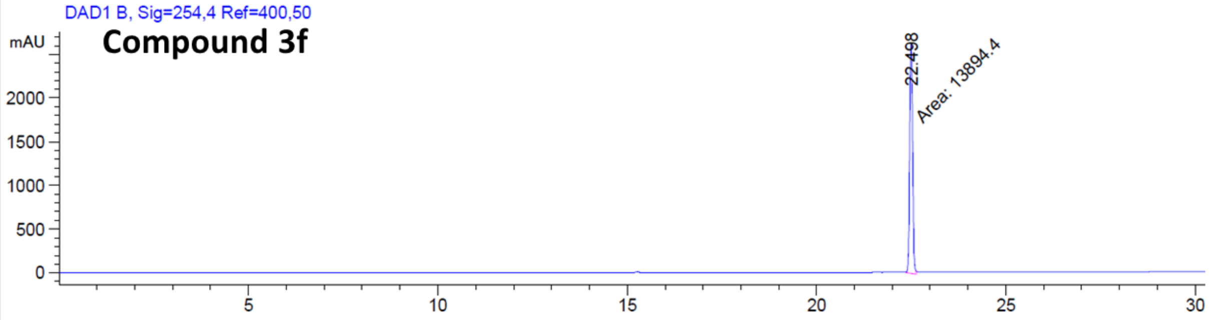

Signal 2: DAD1 B, Sig=254,4 Ref=400,50

Peak #	RetTime [min]	Type	Width [min]	Area [mAU*s]	Height [mAU]	Area %
1	22.498	MM	0.0878	1.38944e4	2637.56958	100.0000
Totals :				1.38944e4	2637.56958	

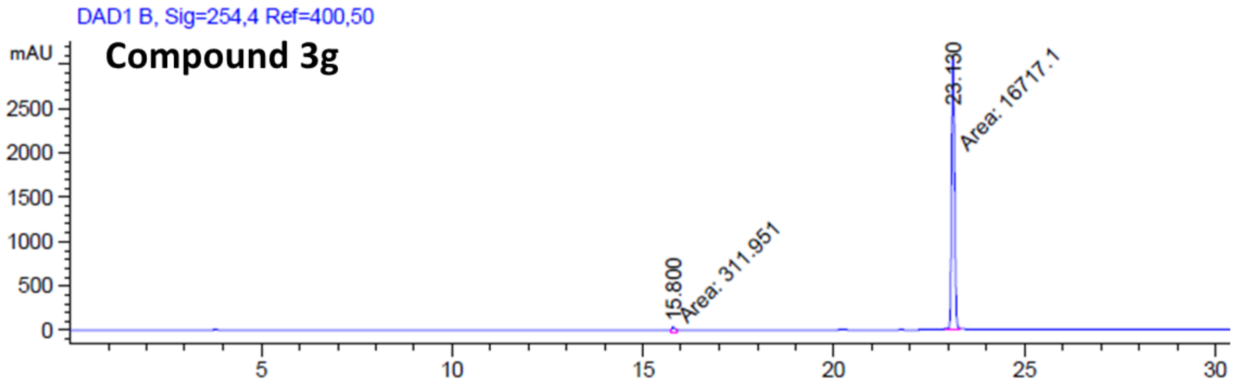

Signal 2: DAD1 B, Sig=254,4 Ref=400,50

Peak #	RetTime [min]	Type	Width [min]	Area [mAU*s]	Height [mAU]	Area %
1	15.800	MM	0.1008	311.95056	51.57749	1.8319
2	23.130	MM	0.0896	1.67171e4	3110.04883	98.1681
Totals :				1.70290e4	3161.62632	

VIII. HRMS of Compounds 2a – f and 3a – g.

Compound 2a

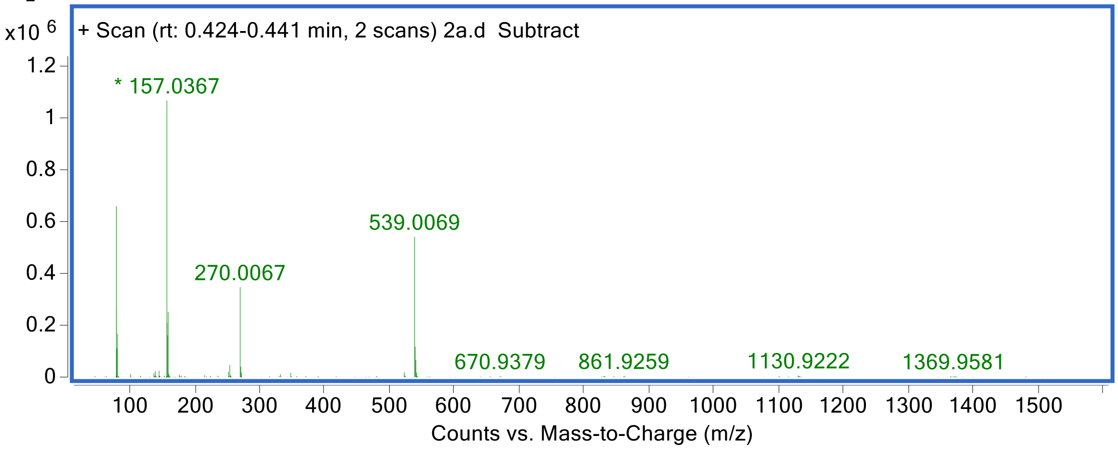

HRMS (ESI) m/z calcd. for $C_{10}H_8NO_6S^+$ (MH^+), 270.0067; found, 270.0067 (Δ = -0.06 ppm).
HRMS (ESI) m/z calcd. for $C_{20}H_{15}N_2O_{12}S_2^+$ (M_2H^+), 539.0061; found, 539.0069 (Δ = -1.5 ppm).

Compound 2b

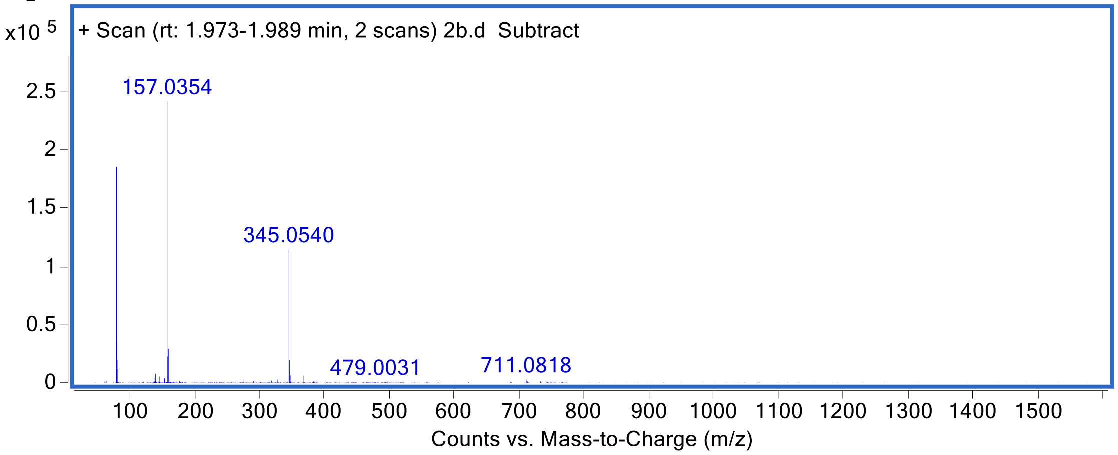

HRMS (ESI) m/z calcd. for $C_{16}H_{13}N_2O_5S^+$ (MH^+), 345.0540; found, 345.0540 (Δ = -0.09 ppm).

Compound 2c

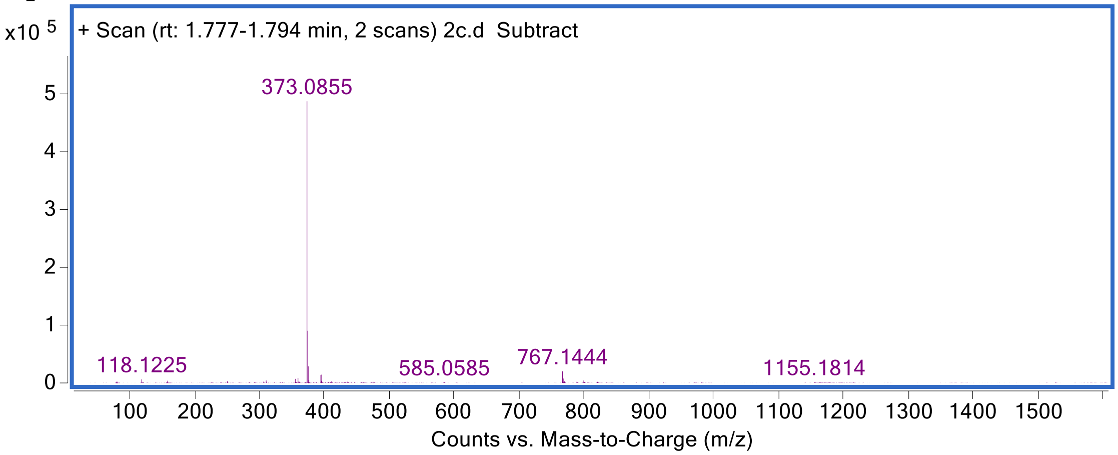

HRMS (ESI) m/z calcd. for $C_{18}H_{17}N_2O_5S^+$ (MH^+), 373.0853 (Δ = -0.62 ppm); found, 373.0855.

HRMS (ESI) m/z calcd. for $C_{36}H_{32}N_4O_{10}S_2Na^+$ (M_2Na^+), 767.1452; found, 767.1444 (Δ = 1.05 ppm).

Compound 2d

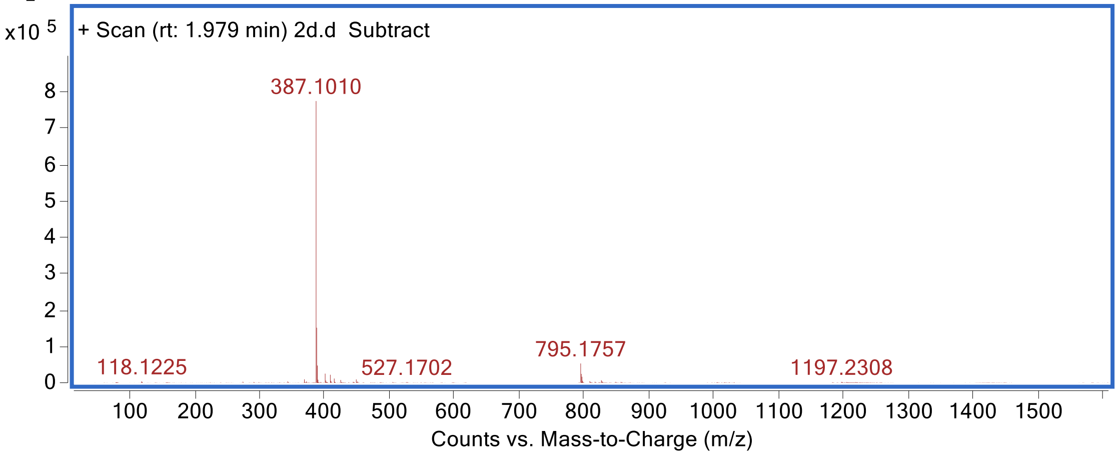

HRMS (ESI) m/z calcd. for $C_{19}H_{19}N_2O_5S^+$ (MH^+), 387.1009; found, 387.1010 (Δ = -0.21 ppm).
HRMS (ESI) m/z calcd. for $C_{38}H_{36}N_4O_{10}S_2Na^+$ (M_2Na^+), 765.1765; found, 795.1757 (Δ = 1.01 ppm).

Compound 2e

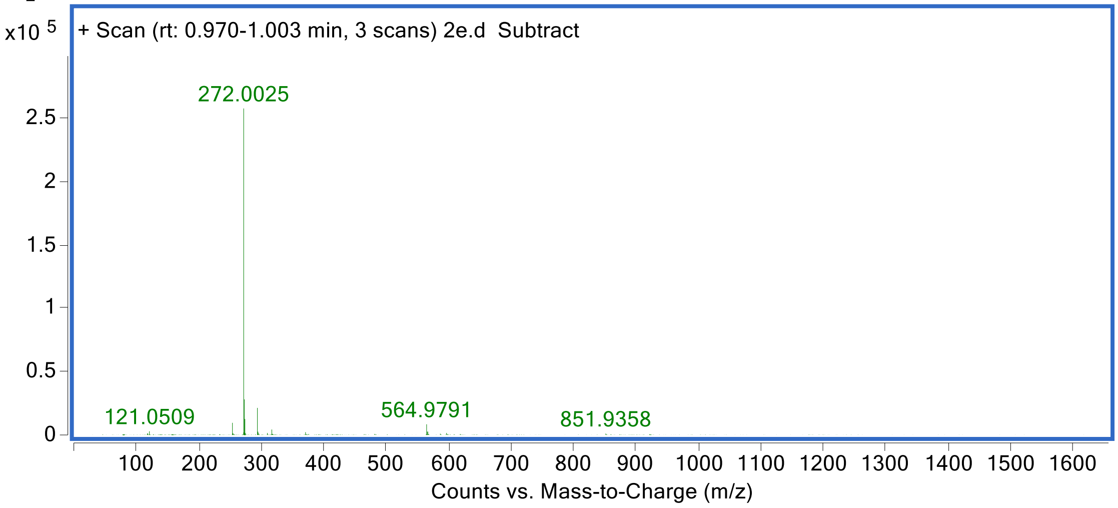

HRMS (ESI) m/z calcd. for $C_{10}H_7FNO_5S^+$ (MH^+), 272.0023; found, 272.0025 (Δ = -0.56 ppm).
HRMS (ESI) m/z calcd. for $C_{20}H_{12}F_2N_2O_{10}S_2Na^+$ (M_2Na^+), 564.9794; found, 564.9791 (Δ = 0.47 ppm).

Compound 2f

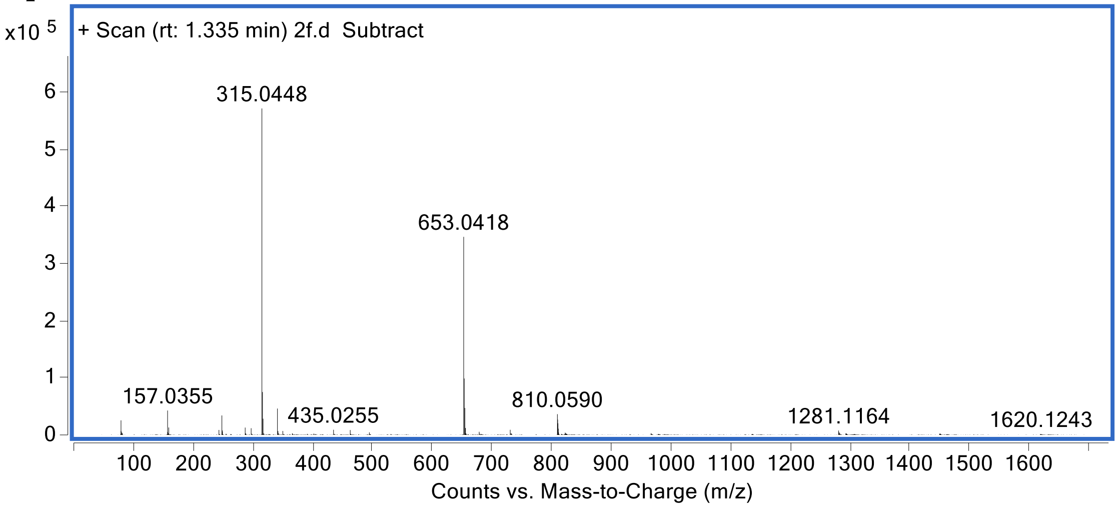

HRMS (ESI) m/z calcd. for $C_{12}H_{12}FN_2O_5S^+$ (MH^+), 315.0445; found, 315.0448 (Δ = -0.8 ppm).

Compound 3a

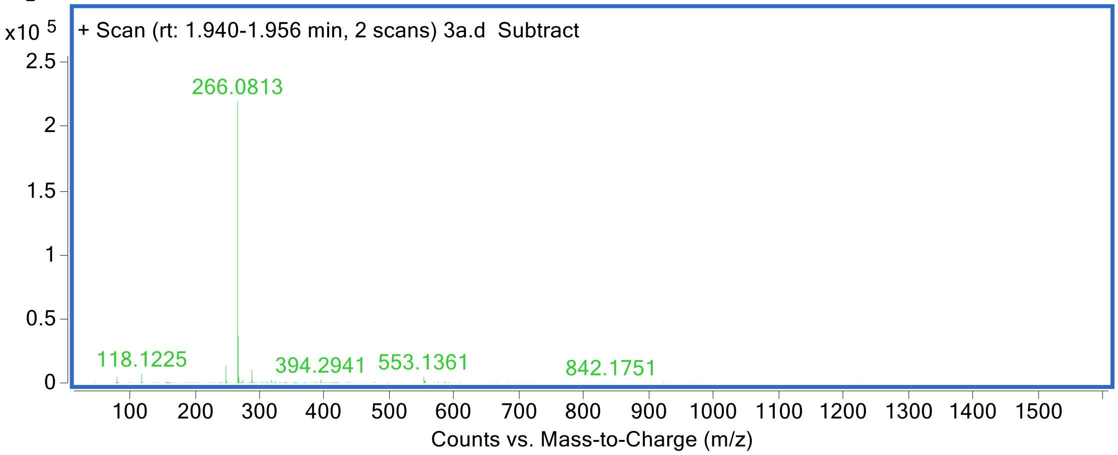

HRMS (ESI) m/z calcd. for $C_{16}H_{12}NO_3$ (MH^+), 266.0812; found, 266.0813 (Δ = -0.49 ppm).

HRMS (ESI) m/z calcd. for $C_{32}H_{22}N_2O_6Na^+$ (M_2Na^+), 553.1370; found, 553.1361 (Δ = 1.64 ppm).

Compound 3b

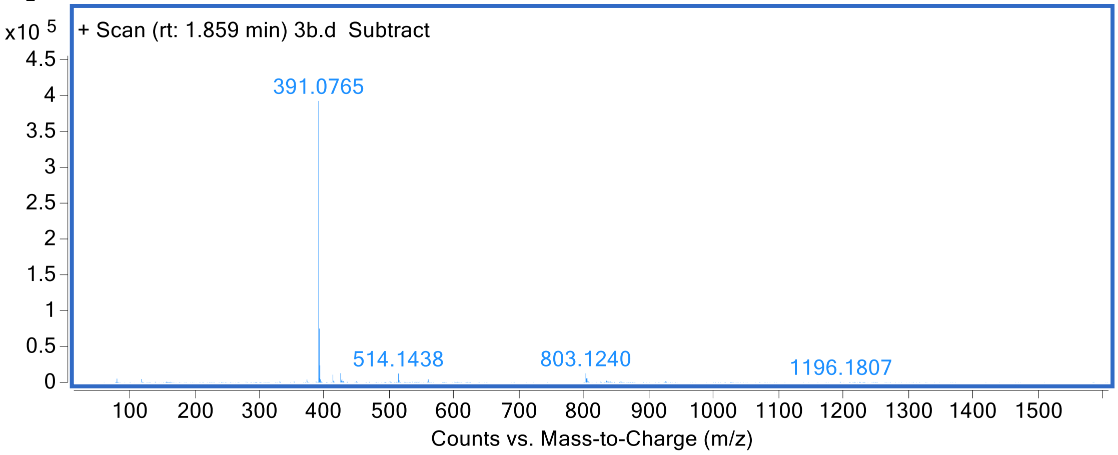

HRMS (ESI) m/z calcd. for $C_{18}H_{16}FN_2O_5S^+$ (MH^+), 391.0758; found, 391.0765 (Δ = -1.67 ppm).

Compound 3c

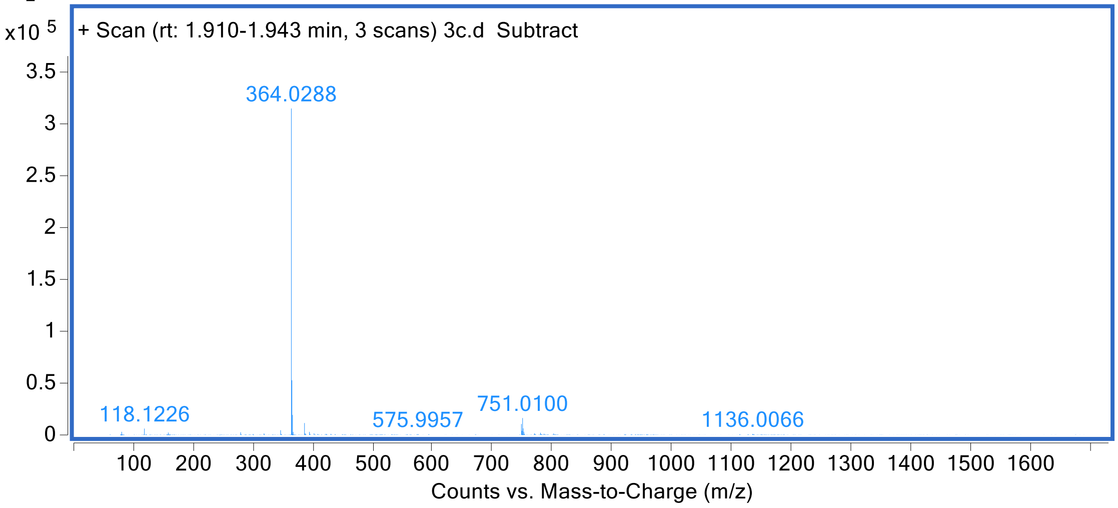

HRMS (ESI) m/z calcd. for $C_{16}H_{11}FNO_6S^+$ (MH^+), 364.0286; found, 364.0288 (Δ = -0.65 ppm).

Compound 3d

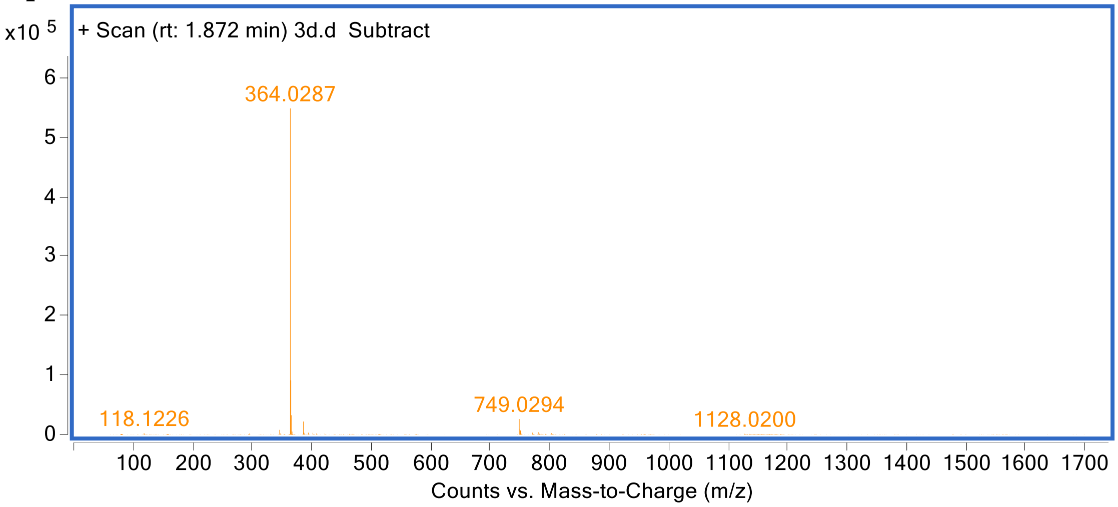

HRMS (ESI) m/z calcd. for $C_{16}H_{11}FNO_6S^+$ (MH^+), 364.0286; found, 364.0287 (Δ = -0.38 ppm).

Compound 3e

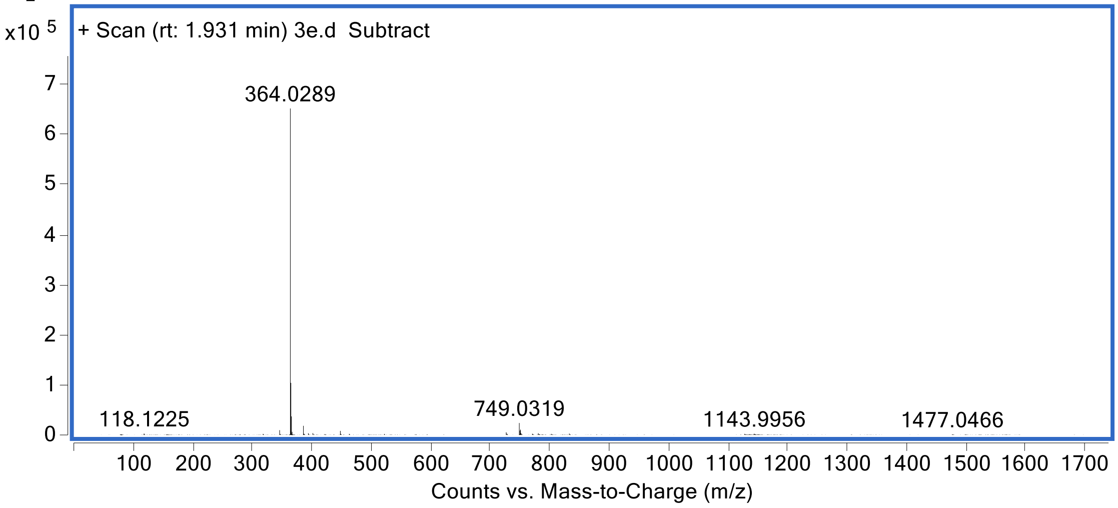

HRMS (ESI) m/z calcd. for $C_{16}H_{11}FNO_6S^+$ (MH^+), 364.0286; found, 364.0289 (Δ = -0.93 ppm).

Compound 3f

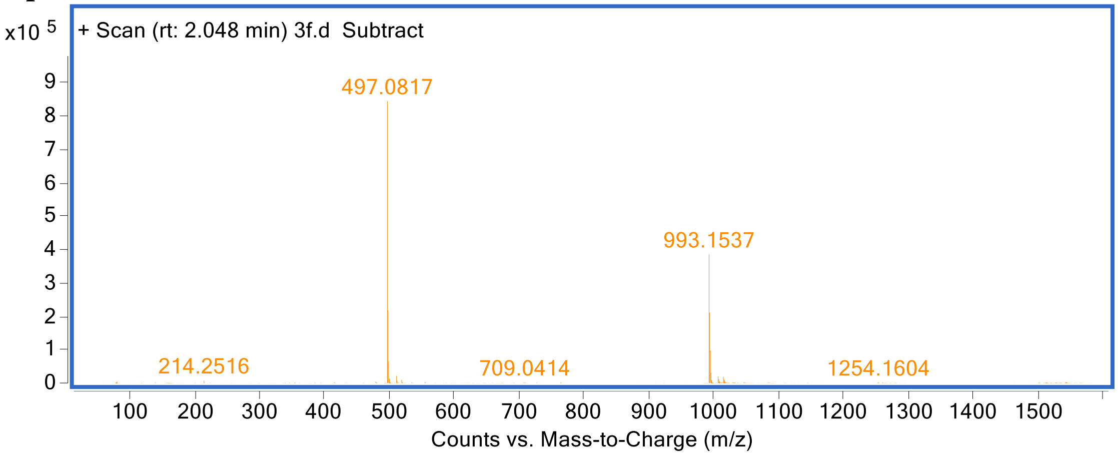

HRMS (ESI) m/z calcd. for $C_{24}H_{18}FN_2O_7S^+$ (MH^+), 497.0813; found, 497.0817 (Δ = -0.75 ppm).

Compound 3g

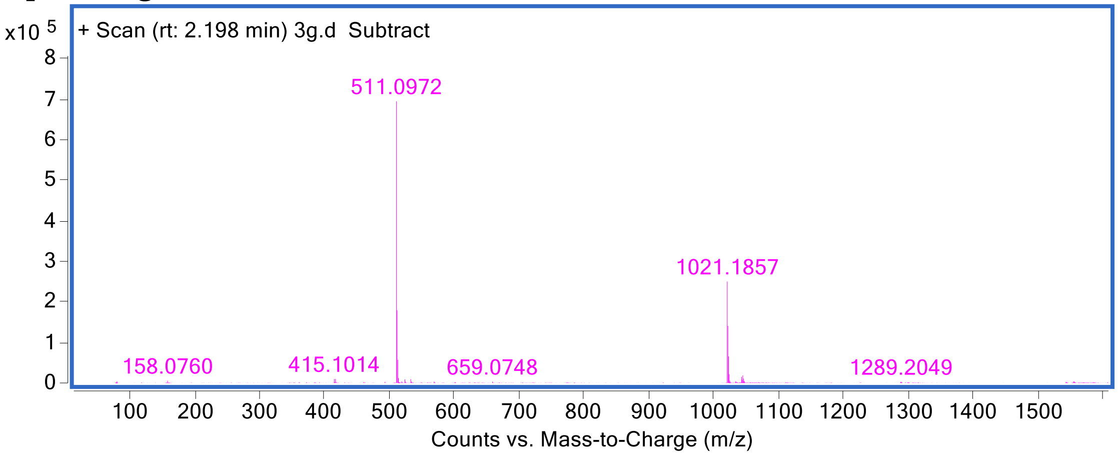

HRMS (ESI) m/z calcd. for $C_{25}H_{20}FN_2O_7S^+$ (MH^+), 511.0970; found, 511.0972 (Δ = -0.44 ppm).

SUPPLEMENTARY REFERENCES

1. Guo, T. *et al.* A new portal to SuFEx click chemistry: a stable fluorosulfuryl imidazolium salt emerging as an "F−SO_2^+" donor of unprecedented reactivity, selectivity, and scope. *Angew. Chem. Int. Ed.* **57**, 2605-2610 (2018). https://doi.org/https://doi.org/10.1002/anie.201712429
2. Lountos, G. T. *et al.* Identification of a ligand binding hot spot and structural motifs replicating aspects of tyrosyl-DNA phosphodiesterase I (TDP1) phosphoryl recognition by crystallographic fragment cocktail screening. *Nucleic Acids Res.* **47**, 10134-10150 (2019). https://doi.org/10.1093/nar/gkz515
3. Krutak, J. J., Burpitt, R. D., Moore, W. H. & Hyatt, J. A. Chemistry of ethenesulfonyl fluoride. Fluorosulfonylethylation of organic compounds. *J. Org. Chem.* **44**, 3847-3858 (1979). https://doi.org/10.1021/jo01336a022
4. Gould, R. G. & Jacobs, W. A. The synthesis of certain substituted quinolines and 5,6-benzoquinolines. *J. Am. Chem. Soc.* **61**, 2890-2895 (1939). https://doi.org/10.1021/ja01265a088
5. Peet, N. P., Baugh, L. E., Sunder, S. & Lewis, J. E. Synthesis and antiallergic activity of some quinolinones and imidazoquinolinones. *J. Med. Chem.* **28**, 298-302 (1985). https://doi.org/10.1021/jm00381a007
6. Davie, B. J. *et al.* Synthesis and pharmacological evaluation of analogues of benzyl quinolone carboxylic acid (BQCA) designed to bind irreversibly to an allosteric site of the M1 muscarinic acetylcholine receptor. *J. Med. Chem.* **57**, 5405-5418 (2014). https://doi.org/10.1021/jm500556a

7. Ludwig, J. & Lehr, M. Convenient synthesis of pyrrole‐ and indolecarboxylic acid tert‐butylesters. *Synth. Commun.* **34**, 3691-3695 (2004). https://doi.org/10.1081/SCC-200032421

8. Miyaura, N., Yamada, K. & Suzuki, A. A new stereospecific cross-coupling by the palladium-catalyzed reaction of 1-alkenylboranes with 1-alkenyl or 1-alkynyl halides. *Tetrahedron Lett.* **20**, 3437-3440 (1979). https://doi.org/https://doi.org/10.1016/S0040-4039(01)95429-2

9. Miyaura, N. & Suzuki, A. Palladium-catalyzed cross-coupling reactions of organoboron compounds. *Chem. Rev.* **95**, 2457-2483 (1995). https://doi.org/10.1021/cr00039a007

10. Ishiyama, T., Murata, M. & Miyaura, N. Palladium(0)-catalyzed cross-coupling reaction of alkoxydiboron with haloarenes: a direct procedure for arylboronic esters. *J. Org. Chem.* **60**, 7508-7510 (1995). https://doi.org/10.1021/jo00128a024

11. Higginbotham, H. F., Yi, C.-L., Monkman, A. P. & Wong, K.-T. Effects of ortho-phenyl substitution on the rISC rate of D–A type TADF molecules. *J. Phys. Chem. C* **122**, 7627-7634 (2018). https://doi.org/10.1021/acs.jpcc.8b01579

12. Ren, Y., Fan, D., Ying, H. & Li, X. Rational design of the benzothiazole-based fluorescent scaffold for tunable emission. *Tetrahedron Lett.* **60**, 1060-1065 (2019). https://doi.org/https://doi.org/10.1016/j.tetlet.2019.03.029

13. Dong, J., Krasnova, L., Finn, M. G. & Sharpless, K. B. Sulfur(VI) fluoride exchange (SuFEx): another good reaction for click chemistry. *Angew. Chem. Int. Ed.* **53**, 9430-9448 (2014). https://doi.org/https://doi.org/10.1002/anie.201309399
