# Supplementary material for: Targeted sulfur(VI) fluoride exchange-mediated covalent modification of a tyrosine residue in the catalytic pocket of tyrosyl-DNA phosphodiesterase 1
Source: Commun Chem. 2024 Sep 16;7:208. doi: 10.1038/s42004-024-01298-w (PMC11405833; doi:10.1038/s42004-024-01298-w)
Supplement: Supplementary file 2 — Description of Additional Supplementary Files [file 42004_2024_1298_MOESM2_ESM.pdf]

Description of Additional Supplementary Files

File name: Supplementary Data 1

Description: Mass spectral analysis.

File name: Supplementary Data 2

Description: Gel image blots.
