# Supplementary material for: Targeted sulfur(VI) fluoride exchange-mediated covalent modification of a tyrosine residue in the catalytic pocket of tyrosyl-DNA phosphodiesterase 1
Source: Commun Chem. 2024 Sep 16;7:208. doi: 10.1038/s42004-024-01298-w (PMC11405833; doi:10.1038/s42004-024-01298-w)
Supplement: Supplementary file 3 — Supplementary Data 1 [file 42004_2024_1298_MOESM3_ESM.pdf]

Label-free analysis of TDP1-3c												Grouped Abundances		%CV		Peptide Raw Intensities (LFQ)						Found in Sample						
Accession	Description	Gene	Annotated Sequence	Modification	#Proteins	# PSMs	Start AA	End AA	# Missed Cleavage	Theo. MH+	Xcorr	3c	DMSO	3c	DMSO	3c_1	3c_2	3c_3	DMSO_1	DMSO_2	DMSO_3	3c_1	3c_2	3c_3	DMSO_1	DMSO_2	DMSO_3	Confidence
Q9NUW8	Tyrosyl-DNA phosphodiesterase 1	TDP1	[K].MMLLLYEEGLR.[V]	2xOxidation [M1; M2]; 1xTDP1-3c [Y6]	1	2	119	129	0	1742.71105	0.72											High	High	Not Found	Not Found	Not Found	Not Found	High
Q9NUW8	Tyrosyl-DNA phosphodiesterase 1	TDP1	[K].MMLLLYEEGLRVVIHTSNLIHADWHQK.[T]	2xOxidation [M1; M2]; 1xTDP1-3c [Y6]	1	11	119	145	1	3621.69635	1.22	1028268.688		37.07		874548.391	1713876.23	1028268.69				High	High	High	Not Found	Not Found	Not Found	High
Q9NUW8	Tyrosyl-DNA phosphodiesterase 1	TDP1	[K].GNPFQFYLTR.[V]	1xTDP1-3c [Y7]	1	9	14	23	0	1585.64164	3.07	6327767.5		23.21		5537360	6327767.5	8586710.44				High	High	High	Not Found	Not Found	Not Found	High
Q9NUW8	Tyrosyl-DNA phosphodiesterase 1	TDP1	[K].QNWLHSYFHKWSAETSGR.[S]	1xTDP1-3c [Y7]	1	9	323	340	1	2577.06767	2.78	2572462.598		59.2		1646828	4018369.75					High	High	High	Not Found	Not Found	Not Found	High
Q9NUW8	Tyrosyl-DNA phosphodiesterase 1	TDP1	[K].MMLLLYEEGLRVVIHTSNLIHADWHQK.[T]	1xTDP1-3c [Y6]	1	2	119	145	1	3589.70652	0.86	166281.2796		57.79			256639.578	107736.555				Not Found	High	High	Not Found	Not Found	Not Found	High
Q9NUW8	Tyrosyl-DNA phosphodiesterase 1	TDP1	[R].SYELGVLFLPSAFGLDSFKVK.[Q]	1xTDP1-3c [Y2]	1	2	389	409	1	2660.26291	1.49											High	Not Found	High	Not Found	Not Found	Not Found	High
Q9NUW8	Tyrosyl-DNA phosphodiesterase 1	TDP1	[R].SYELGVLFLPSAFGLDSFK.[V]	1xTDP1-3c [Y2]	1	2	389	407	0	2433.09953	2.52	158894.4219		25.02		175153.234	105168.555	158894.422				High	High	Peak Found	Not Found	Not Found	Not Found	High
Q9NUW8	Tyrosyl-DNA phosphodiesterase 1	TDP1	[K].QYPPEFR.[K]	1xTDP1-3c [Y2]	1	10	67	73	0	1279.47245	1.05	1551968.75		34.46		1551968.75	1803449.44	866441.781				High	High	High	Not Found	Not Found	Not Found	High
Q9NUW8	Tyrosyl-DNA phosphodiesterase 1	TDP1	[K].TYMRPSPDFSK.[I]	1xTDP1-3c [Y2]	1	3	349	359	0	1671.64541	1.53	446650.0136		8.34		421046.656	473810.281					High	High	High	Not Found	Not Found	Not Found	High
Q9NUW8	Tyrosyl-DNA phosphodiesterase 1	TDP1	[K].TQGIWLSPLYPR.[I]	1xTDP1-3c [Y10]	1	7	146	157	0	1773.79412	3.96	4729781.375		17.16		3927703	4729781.38	5552848.75				High	High	High	High	Not Found	Not Found	High
Q9NUW8	Tyrosyl-DNA phosphodiesterase 1	TDP1	[K].TPGKSSVPLYLIYPSVENVR.[T]	1xTDP1-3c [Y10]	1	7	282	301	1	2562.22211	3.5	2755235.75		18.96		2092773.5	2755235.75	3074397.5				High	High	High	Not Found	Not Found	Not Found	High
Q9NUW8	Tyrosyl-DNA phosphodiesterase 1	TDP1	[K].DRPWIWNIPYVK.[A]	1xTDP1-3c [Y10]	1	3	438	449	0	1929.86287	2.79	461845.75				461845.75						High	High	High	Not Found	Not Found	Not Found	High
Q9NUW8	Tyrosyl-DNA phosphodiesterase 1	TDP1	[R].TSLEGYPAGGSLPYSIQTAEK.[Q]	1xTDP1-3c [Y]	1	5	302	322	0	2512.08607	3.58	8722756		7.81		9912069	8722756	8645878				High	High	High	Not Found	Not Found	Not Found	High
Q9NUW8	Tyrosyl-DNA phosphodiesterase 1	TDP1	[R].TSLEGYPAGGSLPYSIQTAEKQNWLHSYFHKWSAETSGR.[S]	1xTDP1-3c [Y]	1	9	302	340	2	4727.12083	3.13	4197994.776		55.26		2778624	6342405.5					High	High	High	Not Found	Not Found	Not Found	High
Q9NUW8	Tyrosyl-DNA phosphodiesterase 1	TDP1	[K].SSVPLYLIYPSVENVRTSLEGYPAGGSLPYSIQTAEK.[Q]	1xTDP1-3c [Y]	1	3	286	322	1	4329.0584	3.06	2341679		35.76		1326899.13	2341679	2855032.5				Peak Found	High	High	High	Not Found	Not Found	High
Q9NUW8	Tyrosyl-DNA phosphodiesterase 1	TDP1	[K].SSVPLYLIYPSVENVR.[T]	1xTDP1-3c [Y]	1	15	286	301	0	2179.00524	3.75	21181782.75		15.93		19985907.8	21181782.8	26741297				High	High	High	Not Found	Not Found	Not Found	High
Q9NUW8	Tyrosyl-DNA phosphodiesterase 1	TDP1	[K].FFAGSQEPMATFPVPYDLPPELYGSK.[D]	1xOxidation [M9]; 1xTDP1-3c [Y]	1	5	412	437	0	3247.39512	1.8											High	High	High	Not Found	Not Found	Not Found	High
Q9NUW8	Tyrosyl-DNA phosphodiesterase 1	TDP1	[K].TYMRPSPDFSK.[I]	1xOxidation [M3]; 1xTDP1-3c [Y2]	1	5	349	359	0	1687.64032	1.83	2376087		14.87		2704446.75	2376087	2002587.88				High	High	High	Not Found	Not Found	Not Found	High
Q9NUW8	Tyrosyl-DNA phosphodiesterase 1	TDP1	[K].TYMRPSPDFSKIAWFLVTSANLSK.[A]	1xOxidation [M3]; 1xTDP1-3c [Y2]	1	4	349	372	1	3118.43251	1.33	185860.2031				185860.203						High	High	High	Not Found	Not Found	Not Found	High
Q9NUW8	Tyrosyl-DNA phosphodiesterase 1	TDP1	[K].DRPWIWNIPYVKAPDTHGNMWVPS.[-]	1xOxidation [M20]; 1xTDP1-3c [Y10]	1	6	438	461	1	3238.41859	1.16											High	High	High	Not Found	Not Found	Not Found	High
Q9NUW8	Tyrosyl-DNA phosphodiesterase 1	TDP1	[K].WSAETSGRSNAMPHIKTYMRPSPDFSKIAWFLVTSANLSK.[A]	1xOxidation [M19]; 1xTDP1-3c [Y18]	1	2	333	372	3	4871.2691	0.21											Not Found	Not Found	Not Found	Not Found	High	Not Found	High
Q9NUW8	Tyrosyl-DNA phosphodiesterase 1	TDP1	[K].MMLLLYEEGLRVVIHTSNLIHADWHQK.[T]	1xOxidation [M]; 1xTDP1-3c [Y6]	1	13	119	145	1	3605.70143	0.64	418278.8281		37.3		704533.531	360117.547	418278.828				High	High	High	Not Found	Not Found	Not Found	High
Q9NUW8	Tyrosyl-DNA phosphodiesterase 1	TDP1	[K].YNSGALHIKDILSPLFGTLVSSAQFNYCFDVDWLVK.[Q]	1xCarbamidomethyl [C28]; 1xTDP1-3c [Y27]	1	7	31	66	1	4461.08826	4.05	567338.125	62793.96484	115.61		567338.125	184185.156	2563171.5			62793.9648	High	High	High	Not Found	High	Peak Found	High
Q9NUW8	Tyrosyl-DNA phosphodiesterase 1	TDP1	[R].EAKAHLHAQAKPYENISLCQAK.[L]	1xCarbamidomethyl [C19]; 1xTDP1-3c [Y13]	1	8	86	107	1	2850.29741	1.11	1085711.875		52.01		1601045.69	1085711.88	497732.625				High	High	High	Not Found	Not Found	Not Found	High
Q9NUW8	Tyrosyl-DNA phosphodiesterase 1	TDP1	[R].EAKAHLHAQAKPYENISLCQAKLDIAFGTHHTK.[M]	1xCarbamidomethyl [C19]; 1xTDP1-3c [Y13]	1	2	86	118	2	4070.92762	0.74	452006.2929		10.21			420480.313	485895.969				High	High	Peak Found	Not Found	Not Found	Not Found	High
Q9NUW8	Tyrosyl-DNA phosphodiesterase 1	TDP1	[K].AHLHAQAKPYENISLCQAK.[L]	1xCarbamidomethyl [C16]; 1xTDP1-3c [Y10]	1	42	89	107	0	2522.12274	5.43	59900886.52		7.99		59900886.5	53738261.6	62976415.7				High	High	High	Not Found	Not Found	Not Found	High
Q9NUW8	Tyrosyl-DNA phosphodiesterase 1	TDP1	[K].AHLHAQAKPYENISLCQAKLDIAFGTHHTK.[M]	1xCarbamidomethyl [C16]; 1xTDP1-3c [Y10]	1	10	89	118	1	3742.75295	4.52	6519511.563		35.2		8466891.44	6519511.56	4018998.22				High	High	High	Not Found	Not Found	Not Found	High
Q9NUW8	Tyrosyl-DNA phosphodiesterase 1	TDP1	[R].SNAMPHIKTYMRPSPDFSKIAWFLVTSANLSK.[A]	2xOxidation [M4; M11]	1	1	341	372	2	3669.85562	0.52											Not Found	High	Not Found	Not Found	Not Found	Not Found	High
Q9NUW8	Tyrosyl-DNA phosphodiesterase 1	TDP1	[K].LDIAFGTHHTKMMLLLYEEGLR.[V]	2xOxidation [M12; M13]	1	1	108	129	1	2620.32619	0.56	812489.5625				812489.563						High	Not Found	Not Found	Not Found	Not Found	Not Found	High
Q9NUW8	Tyrosyl-DNA phosphodiesterase 1	TDP1	[K].LDIAFGTHHTKMMLLLYEEGLRVVIHTSNLIHADWHQK.[T]	2xOxidation [M12; M13]	1	15	108	145	2	4499.31149	3.52	1007525.688	3709273.5	133.78	11.42	1007525.69	335457.781	7397390.38	3621955.25	3709273.5	4437540.13	High	High	High	High	High	High	High
Q9NUW8	Tyrosyl-DNA phosphodiesterase 1	TDP1	[K].MMLLLYEEGLR.[V]	2xOxidation [M1; M2]	1	140	119	129	0	1399.69599	3.53	2362755489	244669446.4	13.04	19.82	2831437994	2362755489	2214324311	244669446	180708330	269765222	High	High	High	High	High	High	High
Q9NUW8	Tyrosyl-DNA phosphodiesterase 1	TDP1	[K].MMLLLYEEGLRVVIHTSNLIHADWHQK.[T]	2xOxidation [M1; M2]	1	157	119	145	1	3278.68128	6.66	196370050.1	194327576	19.53	15	165681920	196370050	244000928	163883043	194327576	221871021	High	High	High	High	High	High	High
Q9NUW8	Tyrosyl-DNA phosphodiesterase 1	TDP1	[K].MMLLLYEEGLRVVIHTSNLIHADWHQKTQGIWLSPLYPR.[I]	2xOxidation [M1; M2]	1	15	119	157	2	4690.4425	4.47	2967045.168		66.65		2967045.17	600491.594	3668457.23				High	High	High	Not Found	Not Found	Not Found	High
Q9NUW8	Tyrosyl-DNA phosphodiesterase 1	TDP1	[K].FFAGSQEPMATFPVPYDLPPELYGSK.[D]	1xOxidation [M9]	1	128	412	437	0	2904.38006	5.59	982676738.6	483442351.4	4.55	5.98	982676739	946758122	1036157801	503880620	447375331	483442351	High	High	High	High	High	High	High
Q9NUW8	Tyrosyl-DNA phosphodiesterase 1	TDP1	[K].FFAGSQEPMATFPVPYDLPPELYGSKDRPWIWNIPYVK.[A]	1xOxidation [M9]	1	62	412	449	1	4472.21003	4.57	441668473.7	380710813.7	4.93	13.31	415917807	441668474	458946063	388493171	302503645	380710814	High	High	High	High	High	High	High
Q9NUW8	Tyrosyl-DNA phosphodiesterase 1	TDP1	[K].LLKDHASSMPNAESWPVVGQFSSVGSLGADESK.[W]	1xOxidation [M9]	1	26	231	263	1	3446.65327	7.55	13004149.88	4115400.5	14.02	34.88	13004149.9	15159065.8	11472883	5597231.16	4115400.5	2709049.67	High	High	High	High	High	High	High
Q9NUW8	Tyrosyl-DNA phosphodiesterase 1	TDP1	[K].ADLISYLMAYNAPSLK.[E]	1xOxidation [M8]	1	14	174	189	0	1785.90915	4.83	26085807	9929239	7.5	43.12	23190610	26775727	26085807	7689905.5	9929239	17276833.5	High	High	High	High	High	High	High
Q9NUW8	Tyrosyl-DNA phosphodiesterase 1	TDP1	[K].APDTHGNMWVPS.[-]	1xOxidation [M8]	1	200	450	461	0	1327.57356	1.67	5284629660	2771668013	42.65	14.73	1.0126E+10	5284629660	4953224488	2771668013	2131665733	2801175218	High	High	High	High	High	High	High
Q9NUW8	Tyrosyl-DNA phosphodiesterase 1	TDP1	[K].ADLISYLMAYNAPSLKEWIDVIHKHDLSETNVYLIGSTPGR.[F]	1xOxidation [M8]	1	7	174	214	2	4646.35994	3.06	1508239.625	1060752.875	26.74		1276892.13	2123362.25	1508239.63			1060752.88	High	High	High	High	High	High	High
Q9NUW8	Tyrosyl-DNA phosphodiesterase 1	TDP1	[K].NGTQLMIRSYELGVLFLPSAFGLDSFK.[V]	1xOxidation [M6]	1	24	381	407	1	3019.55976	7.74	12284349	9496526.125	11.79	4.13	10705289.4	12284349	13573826	9731172	9496526.13	8973177.67	High	High	High	High	High	High	High
Q9NUW8	Tyrosyl-DNA phosphodiesterase 1	TDP1	[K].DHASSMPNAESWPVVGQFSSVGSLGADESK.[W]	1xOxidation [M6]	1	252	234	263	0	3092.39018	7.38	1480064959	658903340.7	7.93	8.92	1545090457	1480064959	1321592109	759535713	648162644	658903341	High	High	High	High	High	High	High
Q9NUW8	Tyrosyl-DNA phosphodiesterase 1	TDP1	[K].NGTQLMIRSYELGVLFLPSAFGLDSFKVK.[Q]	1xOxidation [M6]	1	105	381	409	2	3246.72313	6.94	196788533	253074401.3	23.28	11.42	177879051	196788533	272790836	278562783	221331704	253074401	High	High	High	High	High	High	High
Q9NUW8	Tyrosyl-DNA phosphodiesterase 1	TDP1	[K].NGTQLMIR.[S]	1xOxidation [M6]	1	156	381	388	0	948.49313	2.38	5996355276	3224218947	6.41	6.47	6278691335	5996355276	5525459970	3369614801	2962930486	3224218947	High	High	High	High	High	High	High
Q9NUW8	Tyrosyl-DNA phosphodiesterase 1	TDP1	[R].SNAMPHIKTYMRPSPDFSKIAWFLVTSANLSK.[A]	1xOxidation [M4]	1	1	341	372	2	3653.8607	1.27											Not Found	Not Found	High	Not Found	Not Found	Not Found	High
Q9NUW8	Tyrosyl-DNA phosphodiesterase 1	TDP1	[R].SNAMPHIK.[T]	1xOxidation [M4]	1	136	341	348	0	913.45601	2.29	10206685.29	6485861.836	24.72	60.82	10206685.3	8595707.49	13847369.8	15861086.2	6485861.84	5638118.43	High	High	High	High	High	High	High
Q9NUW8	Tyrosyl-DNA phosphodiesterase 1	TDP1	[K].TYMRPSPDFSKIAWFLVTSANLSK.[A]	1xOxidation [M3]	1	127	349	372	1	2775.41745	6.27	2098356931	698159945	6.08	19.43	2215981456	2098356931	1961975048	571725206	698159945	845413496	High	High	High	High	High	High	High
Q9NUW8	Tyrosyl-DNA phosphodiesterase 1	TDP1	[K].TYMRPSPDFSK.[I]	1xOxidation [M3]	1	133	349	359	0	1344.62526	3.34	3541476688	993634392.1	9.93	9.02	3241237626	3949057919	3541476688	993634392	866303505	1032624659	High	High	High	High	High	High	High
Q9NUW8	Tyrosyl-DNA phosphodiesterase 1	TDP1	[K].ESMLTLGKESK.[T]	1xOxidation [M3]	1	18	271	281	1	1238.62968	2.48	10263116.52	2410256.094	14.73	15.78	12348206.5	9282114.64	10263116.5	1940480.55	2668677.38	2410256.09	High	High	High	High	High	High	High
Q9NUW8	Tyrosyl-DNA phosphodiesterase 1	TDP1	[K].ESMLTLGK.[E]	1xOxidation [M3]	1	263	271	278	0	894.4601	2.09	4135637581	2463527657	17.24	13.45	5445420766	4135637581	4046125106	2463527657	1988102764	2584768710	High	High	High	High	High	High	High
Q9NUW8	Tyrosyl-DNA phosphodiesterase 1	TDP1	[K].TYMRPSPDFSKIAWFLVTSANLSKAAWGALEK.[N]	1xOxidation [M3]	1	68	349	380	2	3601.85119	9.13	20325092.34	35285540.86	7.88	28.62	20325092.3	17883194.3	20752187.9	41955412	35285540.9	23083862.5	High	High	High	High	High	High	High
Q9NUW8	Tyrosyl-DNA phosphodiesterase 1	TDP1	[K].DRPWIWNIPYVKAPDTHGNMWVPS.[-]	1xOxidation [M20]	1	41	438	461	1	2895.40353	5.14	101031553.7	150756793.8	11.97	10.2	112860989	101031554	88711721	171077794	140170758	150756794	High	High	High	High	High	High	High
Q9NUW8	Tyrosyl-DNA phosphodiesterase 1	TDP1	[K].SGESPTHFKADLISYLMAYNAPSLK.[E]	1xOxidation [M17]	1	37	165	189	1	2756.35999	7.98	82120512	63663424.63	5.95	29.64	76245885	85852746	82120512	63663424.6	60565502.1	100481029	High	High	High	High	High	High	High
Q9NUW8	Tyrosyl-DNA phosphodiesterase 1	TDP1	[K].AAWGALEKNGTQLMIRSYELGVLFLPSAFGLDSFKVK.[Q]	1xOxidation [M14]	1	34	373	409	3	4073.15687	6.66	17783391.19	19732398.25	43.37	32.79	15676966	17783391.2	33361538.6	19311700.8	19732398.3	33194801.8	High	High	High	High	High	High	High
Q9NUW8	Tyrosyl-DNA phosphodiesterase 1	TDP1	[K].AAWGALEKNGTQLMIR.[S]	1xOxidation [M14]	1	27	373	388	1	1774.92686	4.53	19309532	16571151.35	5.56	16.24	18327854.2	20479837.1	19309532	15614669.7	16571151.4	21013209.7	High	High	High	High	High	High	High
Q9NUW8	Tyrosyl-DNA phosphodiesterase 1	TDP1	[K].AAWGALEKNGTQLMIRSYELGVLFLPSAFGLDSFK.[V]	1xOxidation [M14]	1	26	373	407	2	3845.99349	5.48	14373021.78	27879433.22	61.67	52.59	7437557.13	14373021.8	27345986.6	27879433.2	10339369.2	35580958.3	High	High	High	High	Peak Found	High	High
Q9NUW8	Tyrosyl-DNA phosphodiesterase 1	TDP1	[R].LKKLLKDHASSMPNAESWPVVGQFSSVGSLGADESK.[W]	1xOxidation [M12]	1	5	228	263	3	3815.92726	2.79	443510.75	185786.026		33.25	443510.75				236084.531	146203.766	High	High	Not Found	High	High	Peak Found	High
Q9NUW8	Tyrosyl-DNA phosphodiesterase 1	TDP1	[K].WSAETSGRSNAMPHIK.[T]	1xOxidation [M12]	1	1	333	348	1	1787.84934	1.33	355458.1563					355458.156					Not Found	High	Not Found	Not Found	Not Found	Not Found	High
Q9NUW8	Tyrosyl-DNA phosphodiesterase 1	TDP1	[K].QKFFAGSQEPMATFPVPYDLPPELYGSKDRPWIWNIPYVK.[A]	1xOxidation [M11]	1	15	410	449	2	4728.36357	3.71	167899527.6	115210926.5	31.77	65.4	167899528	119809371	228998360	128658085	22157476.8	115210927	High	High	High	High	High	High	High
Q9NUW8	Tyrosyl-DNA phosphodiesterase 1	TDP1	[K].QKFFAGSQEPMATFPVPYDLPPELYGSK.[D]	1xOxidation [M11]	1	27	410	437	1	3160.5336	4.34	55732916.75	44273109.5	18.56	10.9	47005360	55732916.8	68040606.8	44273109.5	39817893.8	49518470	High	High	High	High	High	High	High
Q9NUW8	Tyrosyl-DNA phosphodiesterase 1	TDP1	[-].SGEGQDIWDMLDK.[G]	1xOxidation [M10]	1	258	1	13	0	1509.6526	4.4	7959418393	3367920093	9.78	23.95	7959418393	7970722664	6687421459	4570391872	2892824721	3367920093	High	High	High	High	High	High	High
Q9NUW8	Tyrosyl-DNA phosphodiesterase 1	TDP1	[-].SGEGQDIWDMLDKGNPFQFYLTR.[V]	1xOxidation [M10]	1	47	1	23	1	2733.26134	8.32	68726816.28	93043425.19	13.92	9.61	68726816.3	63648699.7	82918361.6	108122414	93043425.2	90983996.3	High	High	High	High	High	High	High
Q9NUW8	Tyrosyl-DNA phosphodiesterase 1	TDP1	[-].SGEGQDIWDMLDKGNPFQFYLTRVSGVKPK.[Y]	1xOxidation [M10]	1	12	1	30	2	3428.69435	5.91	25366403.5	17170722.5	15.84	19.02	25366403.5	27291802.5	19905253	14816499	17170722.5	21502289.5	High	High	High	High	High	High	High
Q9NUW8	Tyrosyl-DNA phosphodiesterase 1	TDP1	[K].KLLKDHASSMPNAESWPVVGQFSSVGSLGADESK.[W]	1xOxidation [M10]	1	32	230	263	2	3574.74824	5.19	6683533	3795221	12.01	6.05	7919297.25	6322485	6683533	4198421.63	3780185	3795221	High	High	High	High	High	High	High
Q9NUW8	Tyrosyl-DNA phosphodiesterase 1	TDP1	[K].TYMRPSPDFSKIAWFLVTSANLSKAAWGALEKNGTQLMIR.[S]	1xOxidation [M]	1	4	349	388	3	4515.33156	1.97		538982.0625		50.95				538982.063	794914.125	255362.922	Not Found	Not Found	High	High	High	Peak Found	High
Q9NUW8	Tyrosyl-DNA phosphodiesterase 1	TDP1	[K].MMLLLYEEGLRVVIHTSNLIHADWHQKTQGIWLSPLYPR.[I]	1xOxidation [M]	1	8	119	157	2	4674.44759	3.24	779164.625		48.94		779164.625	772141.422	1692078.5				High	High	High	Not Found	Not Found	Not Found	High
Q9NUW8	Tyrosyl-DNA phosphodiesterase 1	TDP1	[K].LDIAFGTHHTKMMLLLYEEGLRVVIHTSNLIHADWHQK.[T]	1xOxidation [M]	1	11	108	145	2	4483.31657	3.75	318026.3438	259463.8203	45.67	79.88	318026.344	228405.813	552390.156	873697.938	230852.859	259463.82	High	Peak Found	High	High	High	High	High
Q9NUW8	Tyrosyl-DNA phosphodiesterase 1	TDP1	[K].MMLLLYEEGLRVVIHTSNLIHADWHQK.[T]	1xOxidation [M]	1	268	119	145	1	3262.68637	6.23	184915600.8	244315509.2	33.02	6.8	119301320	184915601	238582371	218187646	244315509	247548228	High	High	High	High	High	High	High
Q9NUW8	Tyrosyl-DNA phosphodiesterase 1	TDP1	[K].MMLLLYEEGLR.[V]	1xOxidation [M]	1	96	119	129	0	1383.70107	3.44	503467145.4	328519392.2	5.44	13.2	516476997	503467145	464682932	328519392	282244488	368299960	High	High	High	High	High	High	High
Q9NUW8	Tyrosyl-DNA phosphodiesterase 1	TDP1	[K].LLKDHASSMPNAESWPVVGQFSSVGSLGADESKWLCSEFK.[E]	1xCarbamidomethyl [C36]; 1xOxidation [M9]	1	1	231	270	2	4397.0853	5.54											High	Not Found	Not Found	Not Found	Not Found	Not Found	High
Q9NUW8	Tyrosyl-DNA phosphodiesterase 1	TDP1	[K].DHASSMPNAESWPVVGQFSSVGSLGADESKWLCSEFKESMLTLGK.[E]	1xCarbamidomethyl [C33]; 2xOxidation [M6; M	1	5	234	278	2	4918.26446	6.36	16840456	3338984.25	48.51		16840456	19157178	6318422.5			3338984.25	High	High	High	Not Found	Not Found	Peak Found	High
Q9NUW8	Tyrosyl-DNA phosphodiesterase 1	TDP1	[K].DHASSMPNAESWPVVGQFSSVGSLGADESKWLCSEFKESMLTLGK.[E]	1xCarbamidomethyl [C33]; 1xOxidation [M]	1	14	234	278	2	4902.26955	6.56	11280750.25	12533123.5	15.34	20.2	9853339.25	13360989.3	11280750.3	13120353	8836419	12533123.5	High	High	High	High	High	High	High
Q9NUW8	Tyrosyl-DNA phosphodiesterase 1	TDP1	[K].DHASSMPNAESWPVVGQFSSVGSLGADESKWLCSEFKESMLTLGK.[E]	1xCarbamidomethyl [C33]	1	18	234	278	2	4886.27463	8.15	11956731	15438831	35.58	38.41	7625046.56	11956731	16083020.8	26903992.3	15438831	13696366	High	High	High	High	High	High	High
Q9NUW8	Tyrosyl-DNA phosphodiesterase 1	TDP1	[K].WLCSEFKESMLTLGK.[E]	1xCarbamidomethyl [C3]; 1xOxidation [M10]	1	102	264	278	1	1844.89212	4.99	327867893.7	120576306.1	19.97	2.76	396547574	327867894	264873735	120576306	124941902	118354594	High	High	High	High	High	High	High
Q9NUW8	Tyrosyl-DNA phosphodiesterase 1	TDP1	[K].WLCSEFKESMLTLGKESK.[T]	1xCarbamidomethyl [C3]; 1xOxidation [M10]	1	23	264	281	2	2189.0617	5.99	9142854	8059501.141	23.37	36.04	9142854	7398065.5	11780245.5	5582608.19	11619169	8059501.14	High	High	High	High	High	High	High
Q9NUW8	Tyrosyl-DNA phosphodiesterase 1	TDP1	[K].WLCSEFK.[E]	1xCarbamidomethyl [C3]	1	287	264	270	0	969.44986	1.58	4459087640	4119376526	3.55	6.61	4459087640	4686101910	4377478056	4198151581	3704749310	4119376526	High	High	High	High	High	High	High
Q9NUW8	Tyrosyl-DNA phosphodiesterase 1	TDP1	[K].WLCSEFKESMLTLGK.[E]	1xCarbamidomethyl [C3]	1	37	264	278	1	1828.8972	5.64	149398306	284280702.1	16.33	28.06	113055830	154827839	149398306	410177250	241771042	284280702	High	High	High	High	High	High	High
Q9NUW8	Tyrosyl-DNA phosphodiesterase 1	TDP1	[K].WLCSEFKESMLTLGKESKTPGK.[S]	1xCarbamidomethyl [C3]	1	3	264	285	3	2556.28366	1.53	200440.0382		5.47		192827.094		208353.547				Peak Found	Not Found	High	Not Found	High	Not Found	High
Q9NUW8	Tyrosyl-DNA phosphodiesterase 1	TDP1	[K].WLCSEFKESMLTLGKESK.[T]	1xCarbamidomethyl [C3]	1	14	264	281	2	2173.06679	5.45	7767855.5	18354956.75	21.67	33.26	6087147.38	7767855.5	9454238.25	26550373	13716385.5	18354956.8	High	High	High	High	High	High	High
Q9NUW8	Tyrosyl-DNA phosphodiesterase 1	TDP1	[K].YNSGALHIKDILSPLFGTLVSSAQFNYCFDVDWLVK.[Q]	1xCarbamidomethyl [C28]	1	155	31	66	1	4118.0732	7.44	10857182.29	7951383.262	25.68	41.02	8482494.43	10857182.3	14195958.9	5101695.25	11894046	7951383.26	High	High	High	High	High	High	High
Q9NUW8	Tyrosyl-DNA phosphodiesterase 1	TDP1	[K].AHLHAQAKPYENISLCQAKLDIAFGTHHTKMMLLLYEEGLR.[V]	1xCarbamidomethyl [C16]; 2xOxidation [M31;	1	1	89	129	2	4780.41603	0.58	209614.4063	91615.02344			209614.406				91615.0234		High	Not Found	Not Found	Not Found	Peak Found	Not Found	High
Q9NUW8	Tyrosyl-DNA phosphodiesterase 1	TDP1	[K].AHLHAQAKPYENISLCQAKLDIAFGTHHTK.[M]	1xCarbamidomethyl [C16]	1	37	89	118	1	3399.73789	8.08	77251428.05	49955915.39	29.88	8.38	77251428.1	75317542.6	123963397	45419058.1	49955915.4	53734771.4	High	High	High	High	High	High	High
Q9NUW8	Tyrosyl-DNA phosphodiesterase 1	TDP1	[K].AHLHAQAKPYENISLCQAK.[L]	1xCarbamidomethyl [C16]	1	358	89	107	0	2179.10768	6.45	2765428948	3394384399	8.36	4.99	2765428948	2638633886	3097353548	3394384399	3127744141	3430617250	High	High	High	High	High	High	High
Q9NUW8	Tyrosyl-DNA phosphodiesterase 1	TDP1	[K].AAWGALEK.[N]		1	169	373	380	0	845.45158	2.23	8511332808	6864999007	4.37	6.45	8090411619	8828978081	8511332808	6864999007	6232927798	7062749188	High	High	High	High	High	High	High
Q9NUW8	Tyrosyl-DNA phosphodiesterase 1	TDP1	[K].IAWFLVTSANLSKAAWGALEKNGTQLMIR.[S]		1	1	360	388	2	3189.72413	1.2											Not Found	Not Found	Not Found	Not Found	High	Not Found	High
Q9NUW8	Tyrosyl-DNA phosphodiesterase 1	TDP1	[K].IAWFLVTSANLSKAAWGALEK.[N]		1	7	360	380	1	2276.24376	4.96	1325550	3383424.932		22.03	1325550			3958751.5	2891710.75		High	High	High	High	High	High	High
Q9NUW8	Tyrosyl-DNA phosphodiesterase 1	TDP1	[K].IAWFLVTSANLSK.[A]		1	452	360	372	0	1449.81003	3.15	1995797293	1103529656	12.01	10.2	1617287118	1995797293	2019080724	1114134260	923975745	1103529656	High	High	High	High	High	High	High
Q9NUW8	Tyrosyl-DNA phosphodiesterase 1	TDP1	[R].IADGTHKSGESPTHFK.[A]		1	1	158	173	1	1711.83982	1.06	230749.4219	34641.58594	5.87	30.77	230749.422	209927.578	234415.813	34641.5859	27687.0918	50231.6953	High	Peak Found	Peak Found	Peak Found	Peak Found	Peak Found	High
Q9NUW8	Tyrosyl-DNA phosphodiesterase 1	TDP1	[K].KPILLVHGDKR.[E]		1	17	75	85	1	1275.78957	4.15	4249414.25	2889273.598	27.04	36.36	4249414.25	6527212.5	4148724.13	2889273.6	4391707.5	2148887.5	High	High	High	High	High	High	High
Q9NUW8	Tyrosyl-DNA phosphodiesterase 1	TDP1	[K].KPILLVHGDK.[R]		1	8	75	84	0	1119.68846	2.93	353576.0938	901634.0625	19.62	22.1	284485.781	353576.094	423293.031	1039244.56	660639.063	901634.063	High	High	High	High	High	High	High
Q9NUW8	Tyrosyl-DNA phosphodiesterase 1	TDP1	[R].SNAMPHIKTYMRPSPDFSKIAWFLVTSANLSK.[A]		1	1	341	372	2	3637.86579	2.03											Not Found	Not Found	Not Found	Not Found	High	Not Found	High
Q9NUW8	Tyrosyl-DNA phosphodiesterase 1	TDP1	[K].KLLKDHASSMPNAESWPVVGQFSSVGSLGADESK.[W]		1	19	230	263	2	3558.75332	3.93	1917155.938	5634273.375	80.15	41.39	1607535.5	1917155.94	6301494	10840808.5	5634273.38	5530867.38	High	High	High	High	High	High	High
Q9NUW8	Tyrosyl-DNA phosphodiesterase 1	TDP1	[R].KKPILLVHGDKR.[E]		1	32	74	85	2	1403.88453	3.15	2219823.145	1740548.353	14.57	47.3	1976847.96	2219823.14	2632733.02	1932960.78	1740548.35	664001.475	High	High	High	High	High	High	High
Q9NUW8	Tyrosyl-DNA phosphodiesterase 1	TDP1	[R].KKPILLVHGDK.[R]		1	14	74	84	1	1247.78342	2.78	2932274.25	2142349.938	33.86	32.29	2932274.25	4074143.5	2037801.28	2142349.94	2526692	1277234.36	High	High	High	High	High	High	High
Q9NUW8	Tyrosyl-DNA phosphodiesterase 1	TDP1	[K].HDLSETNVYLIGSTPGRFQGSQK.[D]		1	6	198	220	1	2534.26339	5.04	3060395.5	1062316.223	11.38	7.84	3254780.75	3060395.5	2597007.75		1004955.81	1122950.63	High	High	Peak Found	Not Found	High	High	High
Q9NUW8	Tyrosyl-DNA phosphodiesterase 1	TDP1	[K].HDLSETNVYLIGSTPGR.[F]		1	32	198	214	0	1858.92937	6.37	115623653.1	77847391	2.63	24.37	111945434	117946550	115623653	77847391	61429454	100182609	High	High	High	High	High	High	High
Q9NUW8	Tyrosyl-DNA phosphodiesterase 1	TDP1	[K].GNPFQFYLTRVSGVKPK.[Y]		1	13	14	30	1	1938.05959	2.65	1944280.5	1487662.125	30.46	19.4	1944280.5	1464514	2693948.38	1367619.75	1487662.13	1956343.03	High	High	High	High	High	High	High
Q9NUW8	Tyrosyl-DNA phosphodiesterase 1	TDP1	[K].SSVPLYLIYPSVENVR.[T]		1	422	286	301	0	1835.99018	3.87	9725834565	10195224680	7.4	8.32	8922401059	1.0349E+10	9725834565	1.0453E+10	8922011538	1.0195E+10	High	High	High	High	High	High	High
Q9NUW8	Tyrosyl-DNA phosphodiesterase 1	TDP1	[K].GNPFQFYLTR.[V]		1	275	14	23	0	1242.62658	2.33	11437769303	10419763387	1.05	4.33	1.1438E+10	1.1332E+10	1.1572E+10	1.0691E+10	9819144305	1.042E+10	High	High	High	High	High	High	High
Q9NUW8	Tyrosyl-DNA phosphodiesterase 1	TDP1	[R].FQGSQKDNWGHFR.[L]		1	11	215	227	1	1606.75095	3.79	6415604.5	9259205.121		15.89			6415604.5	8271100		10365354	High	High	High	High	High	High	High
Q9NUW8	Tyrosyl-DNA phosphodiesterase 1	TDP1	[K].SSVPLYLIYPSVENVRTSLEGYPAGGSLPYSIQTAEK.[Q]		1	18	286	322	1	3986.04334	5.43	116855576	46898308	4.56	24.82	111952028	116855576	122622232	30683454	50638474	46898308	High	High	High	High	High	High	High
Q9NUW8	Tyrosyl-DNA phosphodiesterase 1	TDP1	[K].LDIAFGTHHTK.[M]		1	355	108	118	0	1239.64805	3.72	5740202233	5627167237	2.01	8.88	5740202233	5815879961	5589685208	5764896190	4871053255	5627167237	High	High	High	High	High	High	High
Q9NUW8	Tyrosyl-DNA phosphodiesterase 1	TDP1	[K].FFAGSQEPMATFPVPYDLPPELYGSKDRPWIWNIPYVK.[A]		1	54	412	449	1	4456.21511	4.96	250894551.4	336156567.6	38.47	44.27	115963299	250894551	258563239	674037335	327957940	336156568	High	High	High	High	High	High	High
Q9NUW8	Tyrosyl-DNA phosphodiesterase 1	TDP1	[R].SNAMPHIK.[T]		1	37	341	348	0	897.4611	2.55	16963926.61	19106700.56	105.14	101.55	16963926.6	7447101.29	67204700.7	68662872.3	19106700.6	7829397.13	High	High	High	High	High	High	High
Q9NUW8	Tyrosyl-DNA phosphodiesterase 1	TDP1	[K].SGESPTHFKADLISYLMAYNAPSLK.[E]		1	18	165	189	1	2740.36508	7.21	28897576	73613962	23.95	32.55	26099164.5	40473357	28897576	114626446	63008646	73613962	High	High	High	High	High	High	High
Q9NUW8	Tyrosyl-DNA phosphodiesterase 1	TDP1	[K].NGTQLMIRSYELGVLFLPSAFGLDSFKVK.[Q]		1	105	381	409	2	3230.72822	8.08	84131481.82	515458713.9	57.83	24.68	47204789.6	84131481.8	156191411	706234751	515458714	441006527	High	High	High	High	High	High	High
Q9NUW8	Tyrosyl-DNA phosphodiesterase 1	TDP1	[K].MMLLLYEEGLRVVIHTSNLIHADWHQKTQGIWLSPLYPR.[I]		1	5	119	157	2	4658.45267	3.72	728606.625		33.68		469757.656	728606.625	952147.188				High	High	High	Not Found	Not Found	High	High
Q9NUW8	Tyrosyl-DNA phosphodiesterase 1	TDP1	[K].NGTQLMIRSYELGVLFLPSAFGLDSFKVKQK.[F]		1	1	381	411	3	3486.88176	1.12											Not Found	Not Found	Not Found	High	Not Found	Not Found	High
Q9NUW8	Tyrosyl-DNA phosphodiesterase 1	TDP1	[K].QKFFAGSQEPMATFPVPYDLPPELYGSK.[D]		1	22	410	437	1	3144.53868	4.84	18397537.25	29150145.25	26	33.53	13862635.9	23526069.1	18397537.3	48491303.3	27316053.3	29150145.3	High	High	High	High	High	High	High
Q9NUW8	Tyrosyl-DNA phosphodiesterase 1	TDP1	[K].QKFFAGSQEPMATFPVPYDLPPELYGSKDRPWIWNIPYVK.[A]		1	33	410	449	2	4712.36865	5.64	101282068	150739430	35.72	29.03	92988504	101282068	172426127	218520182	125950253	150739430	High	High	High	High	High	High	High
Q9NUW8	Tyrosyl-DNA phosphodiesterase 1	TDP1	[K].QNWLHSYFHK.[W]		1	131	323	332	0	1359.65928	3.91	1252735021	903275541	6.25	27.69	1282986100	1252735021	1137848711	1139595809	643821989	903275541	High	High	High	High	High	High	High
Q9NUW8	Tyrosyl-DNA phosphodiesterase 1	TDP1	[K].MMLLLYEEGLRVVIHTSNLIHADWHQK.[T]		1	340	119	145	1	3246.69145	6.94	262491262.8	445489898.5	38.05	7.54	186810207	262491263	399140528	403133744	468393345	445489899	High	High	High	High	High	High	High
Q9NUW8	Tyrosyl-DNA phosphodiesterase 1	TDP1	[K].QNWLHSYFHKWSAETSGR.[S]		1	119	323	340	1	2234.05261	6.9	247441420	363292084.1	8.43	20.12	246955203	247441420	285140096	492834168	363292084	345114660	High	High	High	High	High	High	High
Q9NUW8	Tyrosyl-DNA phosphodiesterase 1	TDP1	[K].QNWLHSYFHKWSAETSGRSNAMPHIKTYMRPSPDFSK.[I]		1	4	323	359	3	4422.10837	1.4	1006455.688	2174338.125	2.36		1006455.69	1021548.13	975235.5		2174338.13		Peak Found	High	Peak Found	Not Found	High	Not Found	High
Q9NUW8	Tyrosyl-DNA phosphodiesterase 1	TDP1	[K].QYPPEFR.[K]		1	309	67	73	0	936.45739	2.07	2109954014	2647914399	40.03	32.91	2051893198	3956400748	2109954014	1580530541	2647914399	3174109169	High	High	High	High	High	High	High
Q9NUW8	Tyrosyl-DNA phosphodiesterase 1	TDP1	[K].MMLLLYEEGLR.[V]		1	73	119	129	0	1367.70616	3.01	249729614	224337191.6	14.43	22.94	199777232	265730146	249729614	326118125	224337192	222995420	High	High	High	High	High	High	High
Q9NUW8	Tyrosyl-DNA phosphodiesterase 1	TDP1	[K].QYPPEFRK.[K]		1	28	67	74	1	1064.55236	1.1	2318152	1582740.313	17.92	52.43	1949477.88	2790587.75	2318152	2794843.69	1582740.31	960295.438	High	High	High	High	High	High	High
Q9NUW8	Tyrosyl-DNA phosphodiesterase 1	TDP1	[-].SGEGQDIWDMLDK.[G]		1	55	1	13	0	1493.65768	4.1	4682693491	7003685537	20.13	25.01	3617734069	5455826984	4682693491	9407457929	5770627249	7003685537	High	High	High	High	High	High	High
Q9NUW8	Tyrosyl-DNA phosphodiesterase 1	TDP1	[K].LLKDHASSMPNAESWPVVGQFSSVGSLGADESK.[W]		1	11	231	263	1	3430.65836	5.86	4122766.25	5926001.5	10.22	48.66	3662597.5	4497899	4122766.25	10939341	5926001.5	4349871	High	High	High	High	High	High	High
Q9NUW8	Tyrosyl-DNA phosphodiesterase 1	TDP1	[-].SGEGQDIWDMLDKGNPFQFYLTR.[V]		1	31	1	23	1	2717.26643	8.33	78525396.44	290277530.9	35.09	43.48	42435224	89484442.5	78525396.4	576483884	284708224	290277531	High	High	High	High	High	High	High
Q9NUW8	Tyrosyl-DNA phosphodiesterase 1	TDP1	[R].LKKLLKDHASSMPNAESWPVVGQFSSVGSLGADESK.[W]		1	5	228	263	3	3799.93235	1.1	611371.2301	537496.4375	11.45	39.11		563727.688	663041.375	301341.125	700446.438	537496.438	Not Found	Peak Found	High	High	High	High	High
Q9NUW8	Tyrosyl-DNA phosphodiesterase 1	TDP1	[-].SGEGQDIWDMLDKGNPFQFYLTRVSGVKPK.[Y]		1	13	1	30	2	3412.69944	7.16	37836460	81981064	69.81	28.55	12949432	37836460	68255214	113207688	64429580	81981064	High	High	High	High	High	High	High
Q9NUW8	Tyrosyl-DNA phosphodiesterase 1	TDP1	[K].LDIAFGTHHTKMMLLLYEEGLRVVIHTSNLIHADWHQK.[T]		1	31	108	145	2	4467.32166	6.47	4374419.828	5958934.805	71.33	65.64	2080898.09	4374419.83	9483594.2	4158600.25	14104973.9	5958934.8	High	High	High	High	High	High	High
Q9NUW8	Tyrosyl-DNA phosphodiesterase 1	TDP1	[K].NGTQLMIRSYELGVLFLPSAFGLDSFK.[V]		1	22	381	407	1	3003.56484	7.11	6171626.5	12427490.25	47.71	18.67	2569795.63	6171626.5	7626095.47	15639921.5	12427490.3	10887198.5	High	High	High	High	High	High	High
Q9NUW8	Tyrosyl-DNA phosphodiesterase 1	TDP1	[R].SYELGVLFLPSAFGLDSFK.[V]		1	331	389	407	0	2090.08447	4.18	5816620941	4888828473	6.43	32.99	5267882513	5960801120	5816620941	4903301675	2546228549	4888828473	High	High	High	High	High	High	High
Q9NUW8	Tyrosyl-DNA phosphodiesterase 1	TDP1	[K].FFAGSQEPMATFPVPYDLPPELYGSK.[D]		1	48	412	437	0	2888.38514	4.76	150376874	184294512.1	17.77	39.41	122554254	150376874	175680030	307161705	146853834	184294512	High	High	High	High	High	High	High
Q9NUW8	Tyrosyl-DNA phosphodiesterase 1	TDP1	[R].TSLEGYPAGGSLPYSIQTAEKQNWLHSYFHKWSAETSGR.[S]		1	54	302	340	2	4384.10577	6.06	759276100.5	878452386.1	22.58	32.62	564540317	759276101	897253996	1489132664	878452386	877480774	High	High	High	High	High	High	High
Q9NUW8	Tyrosyl-DNA phosphodiesterase 1	TDP1	[R].SYELGVLFLPSAFGLDSFKVK.[Q]		1	182	389	409	1	2317.24785	5.32	1898214650	4821700075	10.74	30.6	1687362735	1898214650	2094074232	5997145926	3159333961	4821700075	High	High	High	High	High	High	High
Q9NUW8	Tyrosyl-DNA phosphodiesterase 1	TDP1	[K].DHASSMPNAESWPVVGQFSSVGSLGADESK.[W]		1	155	234	263	0	3076.39527	6.1	539077353.7	641819320	18.59	14.62	387889382	553579756	539077354	641819320	625610071	808407843	High	High	High	High	High	High	High
Q9NUW8	Tyrosyl-DNA phosphodiesterase 1	TDP1	[K].TYMRPSPDFSKIAWFLVTSANLSK.[A]		1	136	349	372	1	2759.42253	7.88	1203124436	1385663772	40.27	24.56	560759590	1203124436	1342675366	1992903169	1287572883	1385663772	High	High	High	High	High	High	High
Q9NUW8	Tyrosyl-DNA phosphodiesterase 1	TDP1	[K].APDTHGNMWVPS.[-]		1	65	450	461	0	1311.57865	0.49	2293220562	1980398470	29.89	19.17	1291614531	2330215054	2293220562	2582966797	1807484616	1980398470	High	High	High	High	High	High	High
Q9NUW8	Tyrosyl-DNA phosphodiesterase 1	TDP1	[K].TYMRPSPDFSKIAWFLVTSANLSKAAWGALEK.[N]		1	46	349	380	2	3585.85627	9.57	8562359.75	54416714	22.45	49.85	8562359.75	8142255.3	12062601.3	92638431.2	54416714	33475597	High	High	High	High	High	High	High
Q9NUW8	Tyrosyl-DNA phosphodiesterase 1	TDP1	[K].ADLISYLMAYNAPSLKEWIDVIHKHDLSETNVYLIGSTPGR.[F]		1	2	174	214	2	4630.36502	1.38											Not Found	High	Not Found	High	Not Found	Not Found	High
Q9NUW8	Tyrosyl-DNA phosphodiesterase 1	TDP1	[R].VVIHTSNLIHADWHQK.[T]		1	327	130	145	0	1898.00314	5.7	1135425070	596626937.4	23.99	17.93	1570400717	1135425070	1003648312	639347484	447638560	596626937	High	High	High	High	High	High	High
Q9NUW8	Tyrosyl-DNA phosphodiesterase 1	TDP1	[K].ADLISYLMAYNAPSLK.[E]		1	15	174	189	0	1769.91423	3.99	16786943.75	17117886.5	22.07	35.2	12452342.4	19579407.4	16786943.8	26513999	13524530.5	17117886.5	High	High	High	High	High	High	High

Q9NUW8	Tyrosyl-DNA phosphodiesterase 1	TDP1	[R].VVIHTSNLIHADWHQKTQGIWLSPLYPR.[I]		1	19	130	157	1	3309.76436	6.25	9996644.25	8162197.906	37.18	19.04	7454779.19	9996644.25	15433690.9	8162197.91	6791359.75	9942769.03	High	High	High	High	High	High	High
Q9NUW8	Tyrosyl-DNA phosphodiesterase 1	TDP1	[K].AAWGALEKNGTQLMIRSYELGVLFLPSAFGLDSFKVK.[Q]		1	36	373	409	3	4057.16195	8.27	16436562.25	65653242.53	39.85	49.23	11106405.3	16436562.3	24924642.8	101110934	65653242.5	34961864	High	High	High	High	High	High	High
Q9NUW8	Tyrosyl-DNA phosphodiesterase 1	TDP1	[K].WSAETSGR.[S]		1	92	333	340	0	893.41117	1.77	271703524.8	101817801.6	97.15	112.48	271703525	22700773.3	600356418	338938261	101817802	10921208.8	High	High	High	High	High	High	High
Q9NUW8	Tyrosyl-DNA phosphodiesterase 1	TDP1	[K].AAWGALEKNGTQLMIRSYELGVLFLPSAFGLDSFK.[V]		1	31	373	407	2	3829.99858	6.83	13308499.66	34381517.94	33.2	37.81	13308499.7	12400794.7	21956448.8	53224277	34381517.9	25325882.7	High	High	High	High	High	High	High
Q9NUW8	Tyrosyl-DNA phosphodiesterase 1	TDP1	[K].YNSGALHIK.[D]		1	13	31	39	0	1002.53671	2.8	14068863	5964560	36	15.52	9313411	19623444	14068863	5964560	4973826	6808114.5	High	High	High	High	High	High	High
Q9NUW8	Tyrosyl-DNA phosphodiesterase 1	TDP1	[K].AAWGALEKNGTQLMIR.[S]		1	12	373	388	1	1758.93195	5.69	10137265	15059890	14.33	28.95	8137992.25	10137265	10807564	24048299.3	14962336	15059890	High	High	High	High	High	High	High
Q9NUW8	Tyrosyl-DNA phosphodiesterase 1	TDP1	[R].TSLEGYPAGGSLPYSIQTAEKQNWLHSYFHK.[W]		1	24	302	332	1	3509.71244	8.71	178147512	259985671	11.6	21.5	152172604	191935518	178147512	332022094	217254571	259985671	High	High	High	High	High	High	High
Q9NUW8	Tyrosyl-DNA phosphodiesterase 1	TDP1	[R].TSLEGYPAGGSLPYSIQTAEK.[Q]		1	214	302	322	0	2169.071	4.87	6112950063	4339080134	4.47	13.38	5748318349	6275849797	6112950063	5086305502	3911068330	4339080134	High	High	High	High	High	High	High
Q9NUW8	Tyrosyl-DNA phosphodiesterase 1	TDP1	[K].TYMRPSPDFSK.[I]		1	65	349	359	0	1328.63035	2.43	460053400.3	284755113.5	28.79	23.33	276755125	460053400	498757450	386635331	248646800	284755113	High	High	High	High	High	High	High
Q9NUW8	Tyrosyl-DNA phosphodiesterase 1	TDP1	[K].DRPWIWNIPYVK.[A]		1	66	438	449	0	1586.84781	4.3	3080444950	2266532311	9.56	30.06	2714615423	3285953921	3080444950	2891138597	1548479172	2266532311	High	High	High	High	High	High	High
Q9NUW8	Tyrosyl-DNA phosphodiesterase 1	TDP1	[K].EWIDVIHKHDLSETNVYLIGSTPGRFQGSQK.[D]		1	21	190	220	2	3554.80266	8.87	30224330	40632585.5	14.14	26.8	31059318	23738804	30224330	40632585.5	24926819.8	42500098.5	High	High	High	High	High	High	High
Q9NUW8	Tyrosyl-DNA phosphodiesterase 1	TDP1	[K].EWIDVIHKHDLSETNVYLIGSTPGR.[F]		1	21	190	214	1	2879.46863	7.45	46512622.75	48604200	9.62	32.49	40318403	46512622.8	48696916.5	48604200	28264897	56096865.3	High	High	High	High	High	High	High
Q9NUW8	Tyrosyl-DNA phosphodiesterase 1	TDP1	[K].EWIDVIHK.[H]		1	7	190	197	0	1039.55711	2.51	14377316	5669810.5	17.21	41.84	14377316	17805696	12757457	5669810.5	4753310.5	10106817	High	High	High	High	High	High	High
Q9NUW8	Tyrosyl-DNA phosphodiesterase 1	TDP1	[R].SYELGVLFLPSAFGLDSFKVKQK.[F]		1	17	389	411	2	2573.40139	5.66	27496726	53160851	11.97	16.92	26317480	27496726	32807624	44690212	53160851	62796559	High	High	High	High	High	High	High
Q9NUW8	Tyrosyl-DNA phosphodiesterase 1	TDP1	[K].ESMLTLGKESK.[T]		1	12	271	281	1	1222.63476	2.86	7198333	3215093	45.94	37.83	3111789.5	7198333	8772841.13	3581539.25	1593589.25	3215093	High	High	High	High	High	High	High
Q9NUW8	Tyrosyl-DNA phosphodiesterase 1	TDP1	[K].TPGKSSVPLYLIYPSVENVR.[T]		1	14	282	301	1	2219.20704	4.22	41535652	13045781	4.7	7.5	41410496	41535652	44939024	13045781	11696275	13548664	High	High	High	High	High	High	High
Q9NUW8	Tyrosyl-DNA phosphodiesterase 1	TDP1	[K].ESMLTLGK.[E]		1	127	271	278	0	878.46518	1.28	3156929510	4467307233	11.75	15.56	2624917787	3299511597	3156929510	5635369883	4251139949	4467307233	High	High	High	High	High	High	High
Q9NUW8	Tyrosyl-DNA phosphodiesterase 1	TDP1	[K].ESKTPGKSSVPLYLIYPSVENVRTSLEGYPAGGSLPYSIQTAEK.[Q]		1	2	279	322	3	4713.42979	1.02		1121930.25								1121930.25	High	Not Found	Not Found	Not Found	Not Found	High	High
Q9NUW8	Tyrosyl-DNA phosphodiesterase 1	TDP1	[K].ESKTPGKSSVPLYLIYPSVENVR.[T]		1	6	279	301	2	2563.37663	3.66	1125448.125		47.8		2159108.5	1125448.13	908896.5				High	High	High	Not Found	High	High	High
Q9NUW8	Tyrosyl-DNA phosphodiesterase 1	TDP1	[K].DNWGHFR.[L]		1	87	221	227	0	931.41692	2.44	1659079668	1494933239	5.5	11.37	1673280154	1659079668	1512877905	1637837192	1302738386	1494933239	High	High	High	High	High	High	High
Q9NUW8	Tyrosyl-DNA phosphodiesterase 1	TDP1	[K].NGTQLMIR.[S]		1	48	381	388	0	932.49821	2.65	1447694797	1631680938	34.36	16.48	789418501	1447694797	1632521621	2060400397	1631680938	1517647492	High	High	High	High	High	High	High
Q9NUW8	Tyrosyl-DNA phosphodiesterase 1	TDP1	[K].TPGKSSVPLYLIYPSVENVRTSLEGYPAGGSLPYSIQTAEK.[Q]		1	8	282	322	2	4369.26021	5.53	644827.1875				644827.188						High	High	High	High	Not Found	High	High
Q9NUW8	Tyrosyl-DNA phosphodiesterase 1	TDP1	[K].TQGIWLSPLYPR.[I]		1	396	146	157	0	1430.77906	3.46	9716680764	10106430131	6.6	4.57	8756334110	9716680764	9927686076	1.0673E+10	9750639563	1.0106E+10	High	High	High	High	High	High	High
Q9NUW8	Tyrosyl-DNA phosphodiesterase 1	TDP1	[K].DRPWIWNIPYVKAPDTHGNMWVPS.[-]		1	16	438	461	1	2879.40861	4.72	9860687	104990818	44.63	53.17	19170804.2	8963279	9860687	223569920	88046332	104990818	High	High	High	High	High	High	High
Q9NUW8	Tyrosyl-DNA phosphodiesterase 1	TDP1	[K].TQGIWLSPLYPRIADGTHK.[S]		1	6	146	164	1	2153.1502	3.85	2472302	466835.705	68.12	50.43	2472302	3449203.5	557299.813	677857.25	321506.594		Peak Found	High	High	High	High	Not Found	High

Annotated Sequence	Modifications	Grouped Abundance	Sum Grouped Abundance: No modification	Sum Grouped Abundance: Y modified	%modified	normalized %modified	Y residue
[K].QNWLHSYFHKWSAETSGRSNAMPHIKTYMRPSPDFSK.[I]		1006455.688	7332905363	3008597.22	0.00041012	0.82585885	Y497
[K].TYMRPSPDFSKIAWFLVTSANLSK.[A]		1203124436					
[K].TYMRPSPDFSKIAWFLVTSANLSKAAWGALEK.[N]		8562359.75					
[K].TYMRPSPDFSK.[I]		460053400.3					
[K].TYMRPSPDFSKIAWFLVTSANLSK.[A]	1xOxidation [M3]	2098356931					
[K].TYMRPSPDFSK.[I]	1xOxidation [M3]	3541476688					
[K].TYMRPSPDFSKIAWFLVTSANLSKAAWGALEK.[N]	1xOxidation [M3]	20325092.34					
[K].TYMRPSPDFSK.[I]	1xOxidation [M3]; 1xTDP1-3c [Y2]	2376087					
[K].TYMRPSPDFSKIAWFLVTSANLSK.[A]	1xOxidation [M3]; 1xTDP1-3c [Y2]	185860.2031					
[K].TYMRPSPDFSK.[I]	1xTDP1-3c [Y2]	446650.0136					
[K].GNPFQFYLTRVSGVKPK.[Y]		1944280.5	11650168659	6327767.5	0.00054285	1.0931468	Y167
[K].GNPFQFYLTR.[V]		11437769303					
[-].SGEGQDIWDMLDKGNPFQFYLTR.[V]		78525396.44					
[-].SGEGQDIWDMLDKGNPFQFYLTRVSGVKPK.[Y]		37836460					
[-].SGEGQDIWDMLDKGNPFQFYLTR.[V]	1xOxidation [M10]	68726816.28					
[-].SGEGQDIWDMLDKGNPFQFYLTRVSGVKPK.[Y]	1xOxidation [M10]	25366403.5					
[K].GNPFQFYLTR.[V]	1xTDP1-3c [Y7]	6327767.5					
[K].SSVPLYLIYPSVENVR.[T]		9725834565	9885996068	26278697.5	0.00265113	5.33858931	Y438
[K].SSVPLYLIYPSVENVRTSLEGYPAGGSLPYSIQTAEK.[Q]		116855576					
[K].TPGKSSVPLYLIYPSVENVR.[T]		41535652					
[K].ESKTPGKSSVPLYLIYPSVENVR.[T]		1125448.125					
[K].TPGKSSVPLYLIYPSVENVRTSLEGYPAGGSLPYSIQTAEK.[Q]		644827.1875					
[K].SSVPLYLIYPSVENVRTSLEGYPAGGSLPYSIQTAEK.[Q]	1xTDP1-3c [Y]	2341679					
[K].SSVPLYLIYPSVENVR.[T]	1xTDP1-3c [Y]	21181782.75					
[K].TPGKSSVPLYLIYPSVENVR.[T]	1xTDP1-3c [Y10]	2755235.75					
[K].NGTQLMIRSYELGVLFLPSAFGLDSFKVK.[Q]		84131481.82	8103609782	158894.422	1.9607E-05	0.03948368	Y537
[K].NGTQLMIRSYELGVLFLPSAFGLDSFK.[V]		6171626.5					
[R].SYELGVLFLPSAFGLDSFK.[V]		5816620941					
[R].SYELGVLFLPSAFGLDSFKVK.[Q]		1898214650					
[K].AAWGALEKNGTQLMIRSYELGVLFLPSAFGLDSFKVK.[Q]		16436562.25					
[K].AAWGALEKNGTQLMIRSYELGVLFLPSAFGLDSFK.[V]		13308499.66					
[R].SYELGVLFLPSAFGLDSFKVKQK.[F]		27496726					
[K].AAWGALEKNGTQLMIRSYELGVLFLPSAFGLDSFKVK.[Q]	1xOxidation [M14]	17783391.19					
[K].AAWGALEKNGTQLMIRSYELGVLFLPSAFGLDSFK.[V]	1xOxidation [M14]	14373021.78					
[K].NGTQLMIRSYELGVLFLPSAFGLDSFK.[V]	1xOxidation [M6]	12284349					
[K].NGTQLMIRSYELGVLFLPSAFGLDSFKVK.[Q]	1xOxidation [M6]	196788533					
[R].SYELGVLFLPSAFGLDSFK.[V]	1xTDP1-3c [Y2]	158894.4219					
[K].MMLLLYEEGLRVVIHTSNLIHADWHQKTQGIWLSPLYPR.[I]		728606.625	3770926054	1612828.8	0.00042752	0.86089573	Y271
[K].MMLLLYEEGLRVVIHTSNLIHADWHQK.[T]		262491262.8					
[K].MMLLLYEEGLR.[V]		249729614					
[K].LDIAFGTHHTKMMLLLYEEGLRVVIHTSNLIHADWHQK.[T]		4374419.828					
[K].AHLHAQAKPYENISLCQAKLDIAFGTHHTKMMLLLYEEGLR.[V]	1xCarbamidomethyl [C16]; 2xOxidation [M31; M32]	209614.4063					
[K].MMLLLYEEGLRVVIHTSNLIHADWHQKTQGIWLSPLYPR.[I]	1xOxidation [M]	779164.625					
[K].LDIAFGTHHTKMMLLLYEEGLRVVIHTSNLIHADWHQK.[T]	1xOxidation [M]	318026.3438					
[K].MMLLLYEEGLRVVIHTSNLIHADWHQK.[T]	1xOxidation [M]	184915600.8					
[K].MMLLLYEEGLR.[V]	1xOxidation [M]	503467145.4					
[K].MMLLLYEEGLR.[V]	2xOxidation [M1; M2]	2362755489					
[K].MMLLLYEEGLRVVIHTSNLIHADWHQK.[T]	2xOxidation [M1; M2]	196370050.1					
[K].MMLLLYEEGLRVVIHTSNLIHADWHQKTQGIWLSPLYPR.[I]	2xOxidation [M1; M2]	2967045.168					
[K].LDIAFGTHHTKMMLLLYEEGLR.[V]	2xOxidation [M12; M13]	812489.5625					
[K].LDIAFGTHHTKMMLLLYEEGLRVVIHTSNLIHADWHQK.[T]	2xOxidation [M12; M13]	1007525.688					
[K].MMLLLYEEGLRVVIHTSNLIHADWHQK.[T]	1xOxidation [M]; 1xTDP1-3c [Y6]	418278.8281					
[K].MMLLLYEEGLRVVIHTSNLIHADWHQK.[T]	1xTDP1-3c [Y6]	166281.2796					
[K].MMLLLYEEGLRVVIHTSNLIHADWHQK.[T]	2xOxidation [M1; M2]; 1xTDP1-3c [Y6]	1028268.688					
[K].QNWLHSYFHKWSAETSGRSNAMPHIKTYMRPSPDFSK.[I]		1006455.688	2438606509	6770457.37	0.00276868	5.57529947	Y476
[K].QNWLHSYFHK.[W]		1252735021					
[K].QNWLHSYFHKWSAETSGR.[S]		247441420					
[R].TSLEGYPAGGSLPYSIQTAEKQNWLHSYFHKWSAETSGR.[S]		759276100.5					
[R].TSLEGYPAGGSLPYSIQTAEKQNWLHSYFHK.[W]		178147512					
[R].TSLEGYPAGGSLPYSIQTAEKQNWLHSYFHKWSAETSGR.[S]	1xTDP1-3c [Y]	4197994.776					
[K].QNWLHSYFHKWSAETSGR.[S]	1xTDP1-3c [Y7]	2572462.598					
[K].QYPPEFR.[K]		2109954014	2112272166	1551968.75	0.0007342	1.47846187	Y215
[K].QYPPEFRK.[K]		2318152					
[K].QYPPEFR.[K]	1xTDP1-3c [Y2]	1551968.75					
[K].MMLLLYEEGLRVVIHTSNLIHADWHQKTQGIWLSPLYPR.[I]		728606.625	9733624527	4729781.38	0.00048569	0.97802849	Y302
[K].MMLLLYEEGLRVVIHTSNLIHADWHQKTQGIWLSPLYPR.[I]	1xOxidation [M]	779164.625					
[K].MMLLLYEEGLRVVIHTSNLIHADWHQKTQGIWLSPLYPR.[I]	2xOxidation [M1; M2]	2967045.168					
[R].VVIHTSNLIHADWHQKTQGIWLSPLYPR.[I]		9996644.25					
[K].TQGIWLSPLYPR.[I]		9716680764					
[K].TQGIWLSPLYPRIADGTHK.[S]		2472302					
[K].TQGIWLSPLYPR.[I]	1xTDP1-3c [Y10]	4729781.375					
[K].YNSGALHIKDILSPLFGTLVSSAQFNYCFDVDWLVK.[Q]	1xCarbamidomethyl [C28]	10857182.29	10857182.29	567338.125	0.04965969	100	Y204
[K].YNSGALHIKDILSPLFGTLVSSAQFNYCFDVDWLVK.[Q]	1xCarbamidomethyl [C28]; 1xTDP1-3c [Y27]	567338.125					
[K].SSVPLYLIYPSVENVRTSLEGYPAGGSLPYSIQTAEK.[Q]		116855576	6230450466	11064435	0.00177272	3.56972893	Y454
[K].TPGKSSVPLYLIYPSVENVRTSLEGYPAGGSLPYSIQTAEK.[Q]		644827.1875					
[R].TSLEGYPAGGSLPYSIQTAEK.[Q]		6112950063					
[K].SSVPLYLIYPSVENVRTSLEGYPAGGSLPYSIQTAEK.[Q]	1xTDP1-3c [Y]	2341679					
[R].TSLEGYPAGGSLPYSIQTAEK.[Q]	1xTDP1-3c [Y]	8722756					
[K].FFAGSQEPMATFPVPYDLPPELYGSKDRPWIWNIPYVK.[A]		250894551.4	4153081811	461845.75	0.00011119	0.22391036	Y594
[K].QKFFAGSQEPMATFPVPYDLPPELYGSKDRPWIWNIPYVK.[A]		101282068					
[K].QKFFAGSQEPMATFPVPYDLPPELYGSKDRPWIWNIPYVK.[A]	1xOxidation [M11]	167899527.6					
[K].FFAGSQEPMATFPVPYDLPPELYGSKDRPWIWNIPYVK.[A]	1xOxidation [M9]	441668473.7					
[K].DRPWIWNIPYVK.[A]		3080444950					
[K].DRPWIWNIPYVKAPDTHGNMWVPS.[-]		9860687					
[K].DRPWIWNIPYVKAPDTHGNMWVPS.[-]	1xOxidation [M20]	101031553.7					
[K].DRPWIWNIPYVK.[A]	1xTDP1-3c [Y10]	461845.75					
[K].AHLHAQAKPYENISLCQAKLDIAFGTHHTKMMLLLYEEGLR.[V]	1xCarbamidomethyl [C16]; 2xOxidation [M31; M32]	209614.4063	2842889991	67958116.2	0.0233465	47.0129818	Y245
[K].AHLHAQAKPYENISLCQAKLDIAFGTHHTK.[M]	1xCarbamidomethyl [C16]	77251428.05					
[K].AHLHAQAKPYENISLCQAK.[L]	1xCarbamidomethyl [C16]	2765428948					
[K].AHLHAQAKPYENISLCQAK.[L]	1xCarbamidomethyl [C16]; 1xTDP1-3c [Y10]	59900886.52					
[K].AHLHAQAKPYENISLCQAKLDIAFGTHHTK.[M]	1xCarbamidomethyl [C16]; 1xTDP1-3c [Y10]	6519511.563					
[R].EAKAHLHAQAKPYENISLCQAK.[L]	1xCarbamidomethyl [C19]; 1xTDP1-3c [Y13]	1085711.875					
[R].EAKAHLHAQAKPYENISLCQAKLDIAFGTHHTK.[M]	1xCarbamidomethyl [C19]; 1xTDP1-3c [Y13]	452006.2929					
