# Supplementary figures and images for: Targeted sulfur(VI) fluoride exchange-mediated covalent modification of a tyrosine residue in the catalytic pocket of tyrosyl-DNA phosphodiesterase 1
Source: Commun Chem. 2024 Sep 16;7:208. doi: 10.1038/s42004-024-01298-w (PMC11405833; doi:10.1038/s42004-024-01298-w)

Supplementary Data 2. Gel image blots

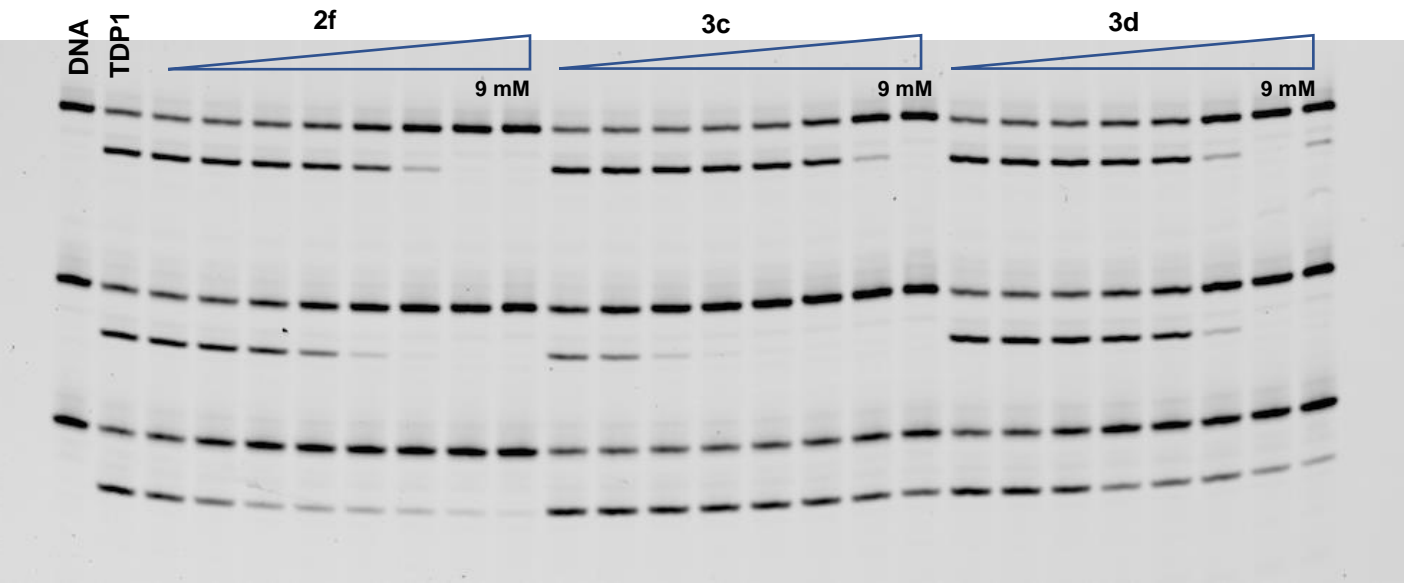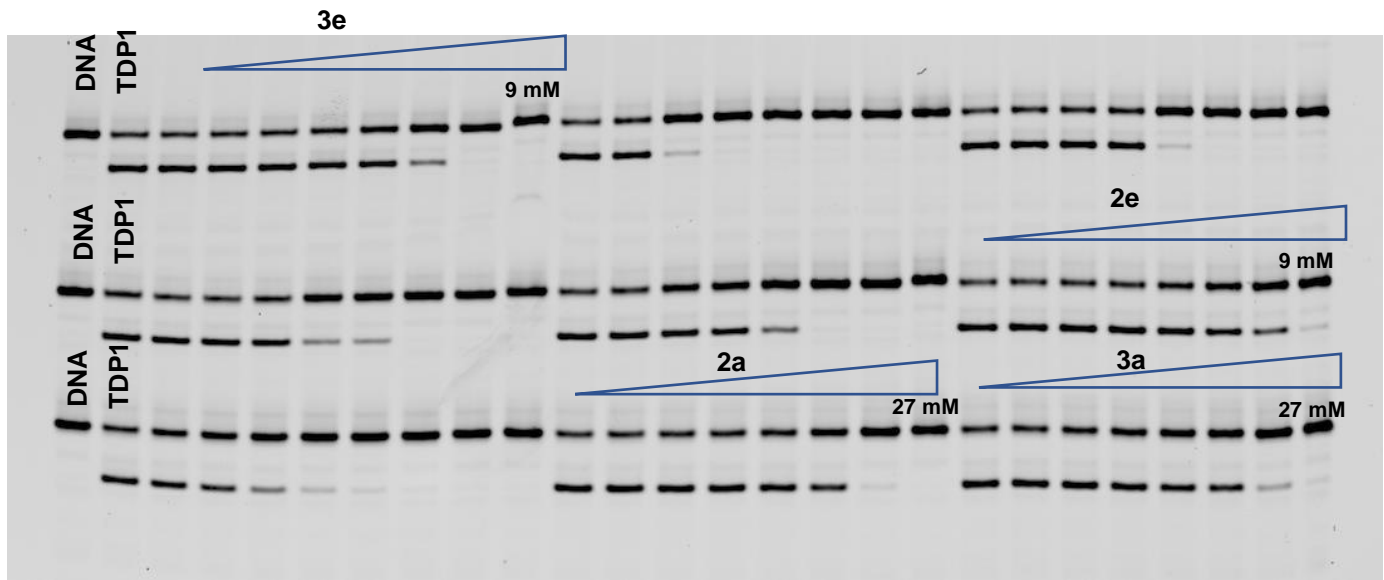

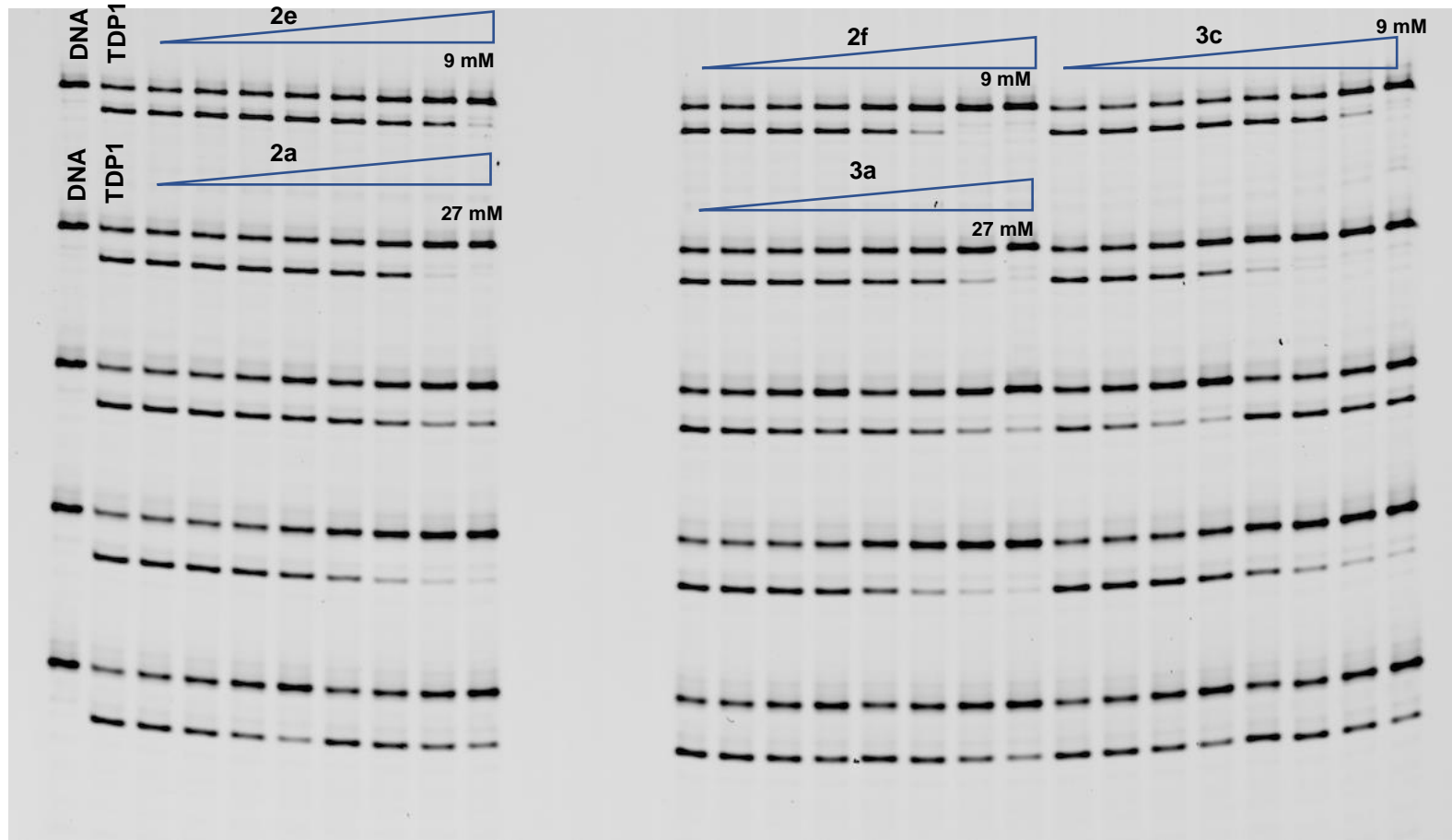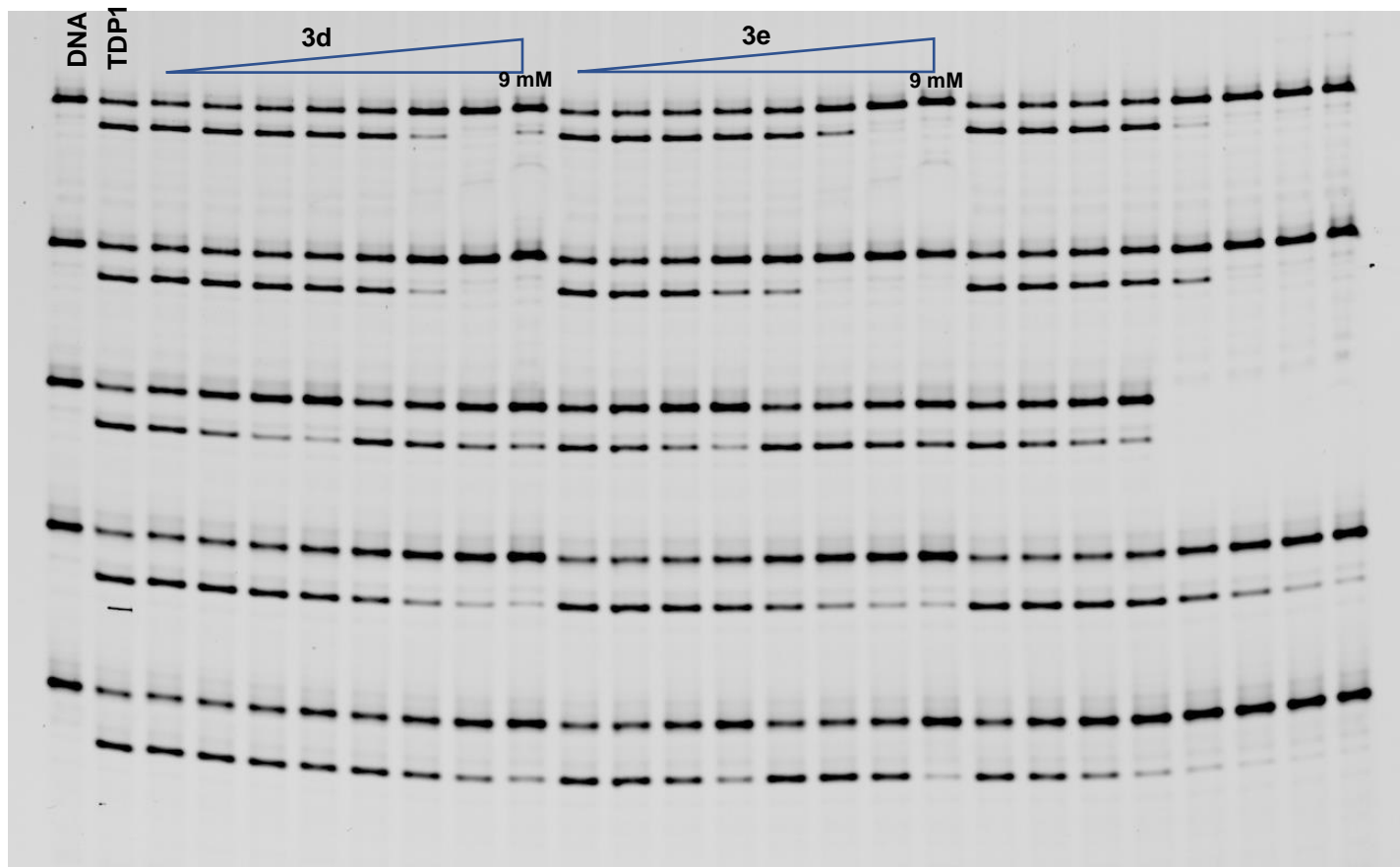

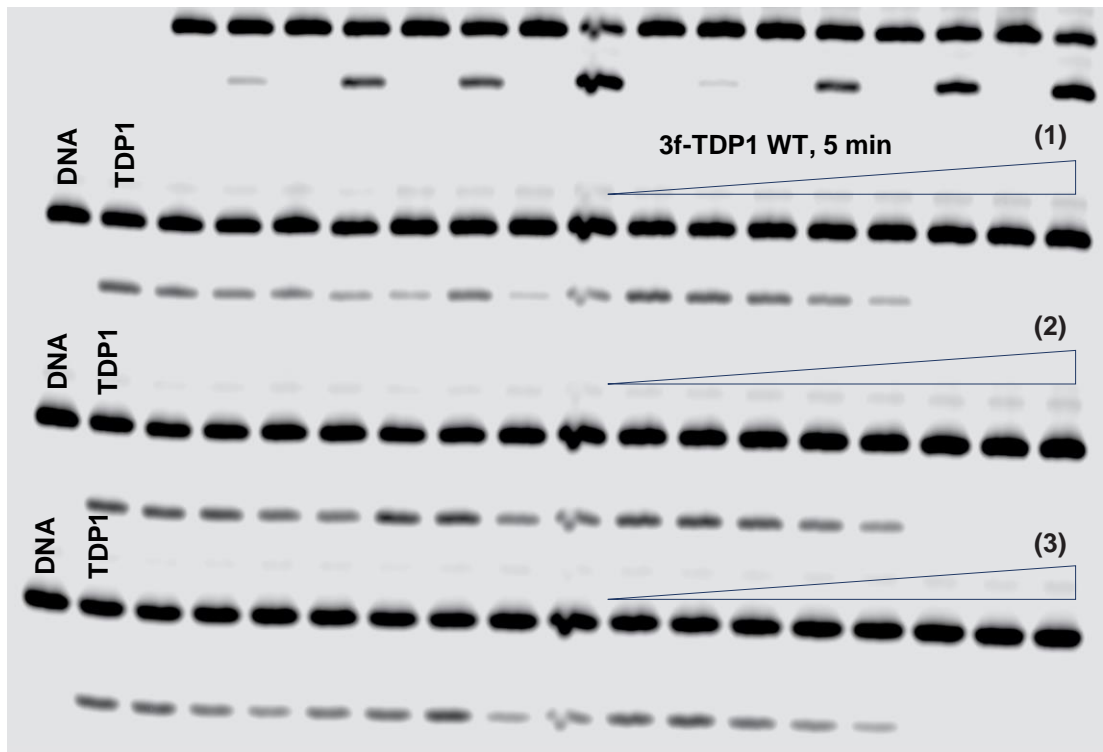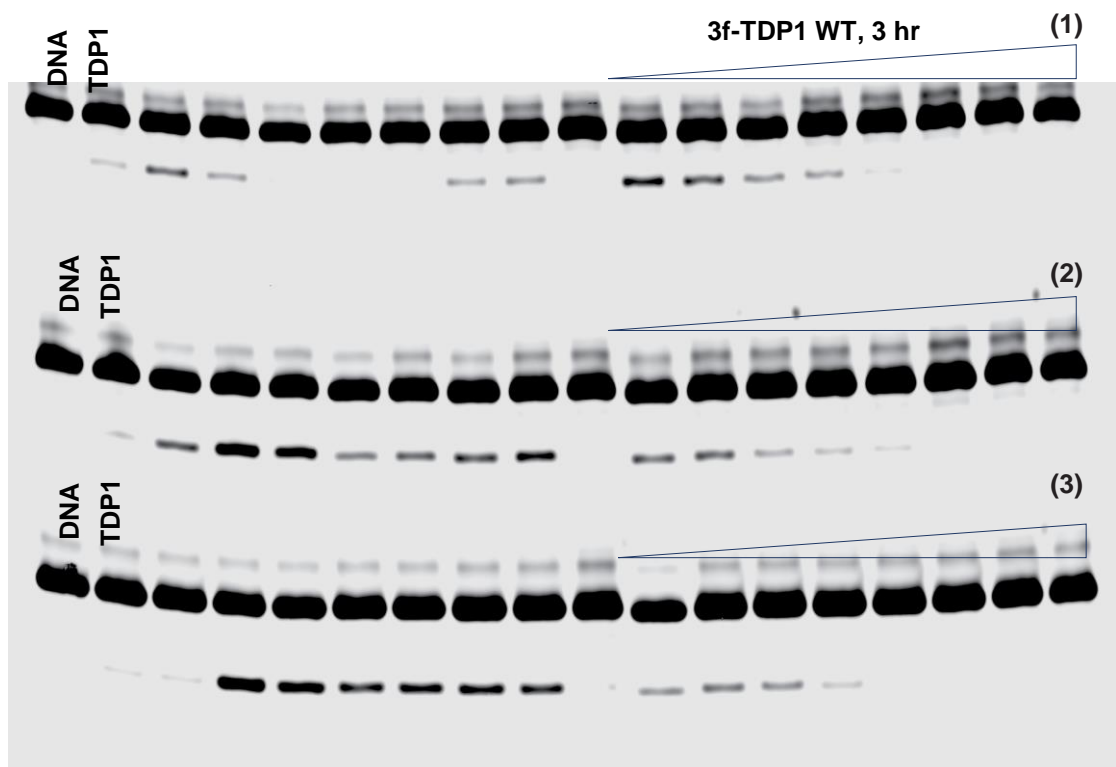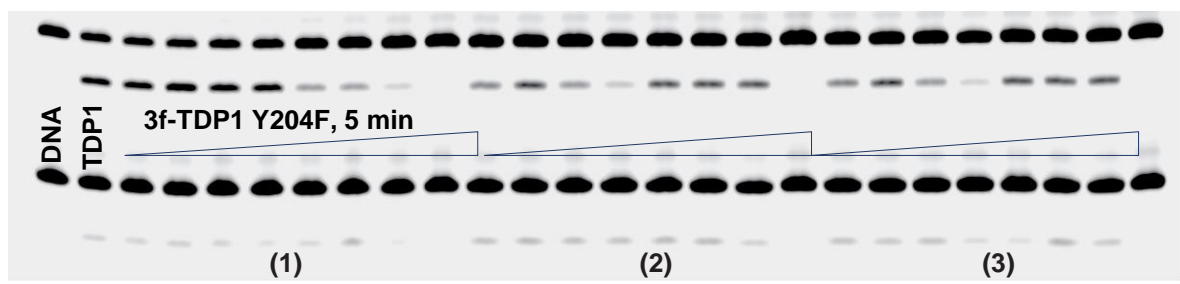

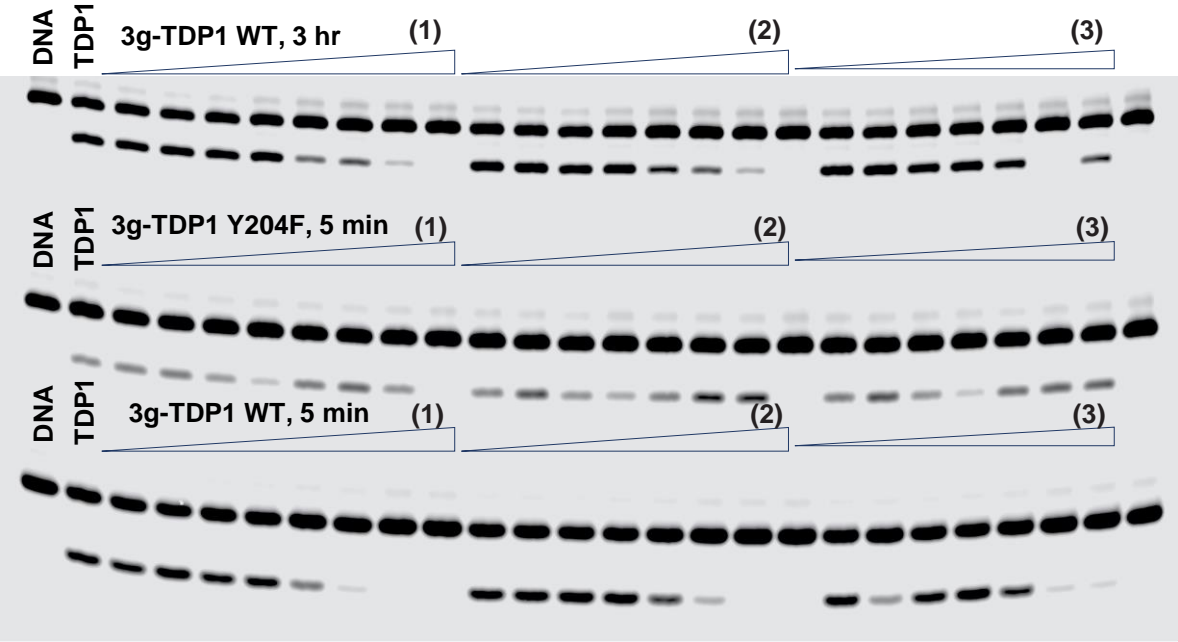

Supplement: Supplementary file 4 — Supplementary Data 2 [file 42004_2024_1298_MOESM4_ESM.pdf]
